# Dual gold/photoredox-catalyzed C(sp)–H arylation of terminal alkynes with diazonium salts†
†Electronic supplementary information (ESI) available: General experimental details, full optimization table, kinetic profile, experimental procedures and characterization data of products, copies of NMR spectra. See DOI: 10.1039/c5sc02583d


**DOI:** 10.1039/c5sc02583d

**Published:** 2015-10-08

**Authors:** Adrian Tlahuext-Aca, Matthew N. Hopkinson, Basudev Sahoo, Frank Glorius

**Affiliations:** a Organisch-Chemisches Institut , Westfälische Wilhelms-Universität Münster , Corrensstraße 40 , 48149 Münster , Germany . Email: glorius@uni-muenster.de; b NRW Graduate School of Chemistry , Westfälische Wilhelms-Universität Münster , Wilhelm-Klemm-Strasse 10 , 48149 Münster , Germany

## Abstract

The arylation of alkyl and aromatic terminal alkynes by a dual gold/photoredox catalytic system is described. Using aryldiazonium salts as readily available aryl sources, a range of diversely-functionalized arylalkynes could be synthesized under mild, base-free reaction conditions using visible light from simple household sources or even sunlight. This process, which exhibits a broad scope and functional group tolerance, expands the range of transformations amenable to dual gold/photoredox catalysis to those involving C–H bond functionalization and demonstrates the potential of this concept to access Au^I^/Au^III^ redox chemistry under mild, redox-neutral conditions.

## Abstract 2

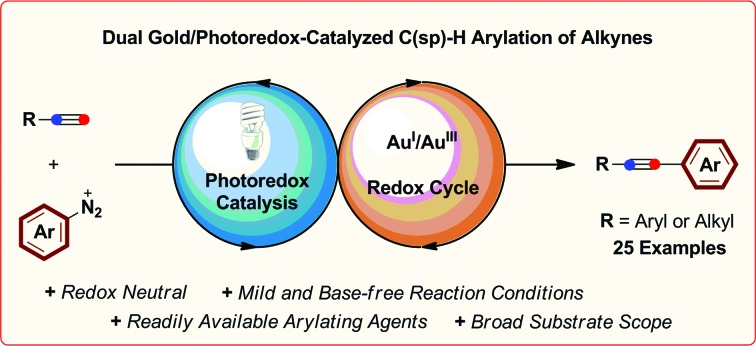
The arylation of alkyl and aromatic terminal alkynes by a dual gold/photoredox catalytic system is described.

## Introduction

**Scheme 1:**
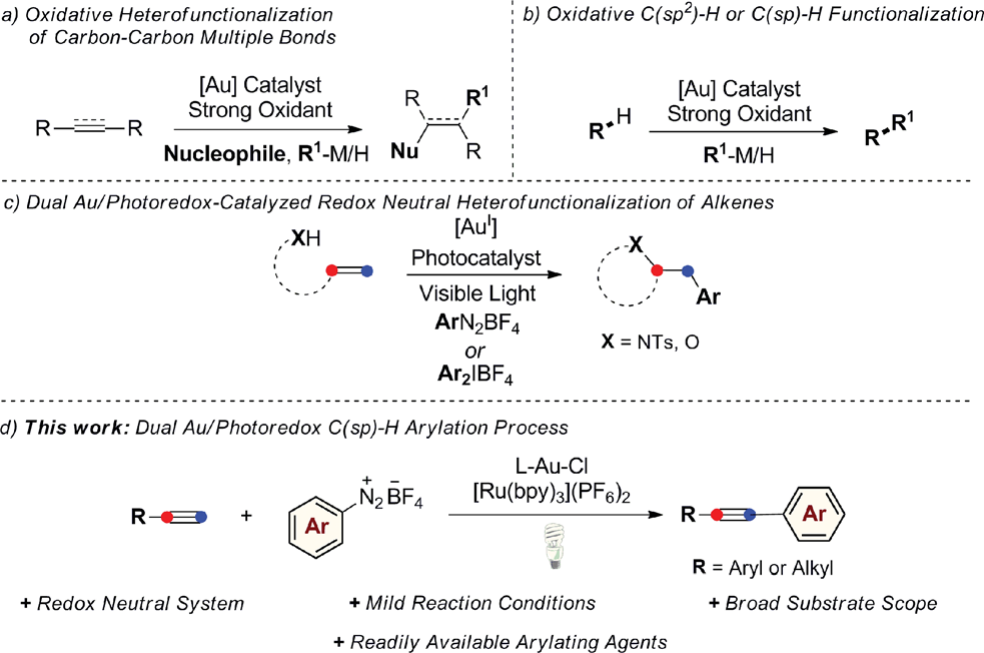
Gold-catalyzed C–C and C–X bond-forming coupling reactions proposed to proceed *via* Au^I^/Au^III^ redox cycles.

Homogeneous gold catalysis has received significant attention in the last two decades. Due to their carbophilic π-acidity, Au^I^ and Au^III^ complexes catalyze the addition of a variety of oxygen-, nitrogen-, carbon-, and sulfur-based nucleophiles to unsaturated molecules, such as alkynes, allenes and alkenes, giving rapid and efficient access to complex molecular architectures.^1^ A common feature regarding the nature of the catalytically active Au species is that it does not easily undergo changes in oxidation state during the course of these reactions. However, there has been significant recent interest in developing Au^I^/Au^III^ catalytic processes with the aim of expanding the repertoire of gold-mediated processes, mimicking conventional M^*n*^/M^*n*+2^ redox cycles of the type commonly invoked in catalysis by other late transition metals.^2^ Nevertheless, in contrast to its isoelectronic counterpart Pd^0^, the oxidation of Au^I^ generally requires the use of strong oxidative conditions due to the high redox potential of the Au^I^/Au^III^ redox couple (*E*_0_ = 1.41 V).^3^ A common strategy to access catalytically active Au^III^ species *in situ* from Au^I^ complexes has been to use strong external oxidants, such as Selectfluor®, *t*BuOOH, or hypervalent iodine reagents. Applying this concept, a range of oxidative homo- and heterocoupling reactions have been developed over the last few years where the oxidative coupling event follows a conventional gold-mediated nucleophilic addition reaction to a C–C multiple bond, circumventing protodeauration and giving efficient access to difunctionalized products (Scheme 1a). This strategy has also been successfully applied in a selection of coupling reactions of unfunctionalized arenes and alkynes, exploiting the well-established ability of gold to activate C(sp^2^)–H or C(sp)–H bonds (Scheme 1b).^4^

Despite the success of these approaches, the use of strong external oxidants, typically in super-stoichiometric amounts, inevitably reduces their attractiveness with regards to functional group tolerance and cost, while most examples are also conducted at high reaction temperatures. With the goal of developing milder gold-catalyzed coupling reactions that do not require external oxidants, our group recently described the use of a dual gold/photoredox catalysis strategy to access Au^I^/Au^III^ redox cycles.^5^,^6^ Using this concept, we have developed intramolecular aminoarylation and both intra-5*a* and intermolecular5*b* oxyarylation reactions of alkenes to afford arylated heterocycles and β-aryl ethers employing aryldiazonium or diaryliodonium salts as general arylating agents (Scheme 1c). This dual catalytic system, which has since been further exploited by the Toste group in an impressive arylative ring expansion7*a* and C–P bond-forming cross-coupling,7*b* operates under mild and overall redox neutral conditions and uses abundant visible light as an energy source. Building on these studies, we sought to expand the scope of dual gold/photoredox catalysis onto new classes of transformations, exploiting different aspects of the rich chemistry of gold. In this regard, we turned our attention to the development of novel visible light-mediated cross-coupling reactions involving gold-catalyzed C–H bond activation and identified the well-established ability of gold(i) to activate the C(sp)–H bond of terminal alkynes as a promising avenue of investigation. Arylation of the resulting alkynylgold complexes would deliver cross-coupled arylalkyne products in a dual gold and photoredox-catalyzed analogue of the widely-employed Sonogashira–Hagihara reaction.^8^,^9^ As demonstrated below, this process benefits from mild conditions (room temperature, household light bulb) and broad functional group tolerance while making use of readily-available aryldiazonium salts as the arene coupling partner. Furthermore, by exploiting a non-conventional stepwise oxidation process of Au^I^ complexes into active Au^III^ species by means of organic radicals generated under photoredox catalysis, we have developed a very fast arylating system that proceeds under base-free conditions.^10^

## Results and discussion

In a preliminary experiment, *p*-tolylacetylene (**1a**) was reacted in the presence of benzenediazonium tetrafluoroborate (**2a**) under visible light irradiation from a simple household desk lamp fitted with a 23 W fluorescent light bulb (CFL). Upon treatment with 10 and 2.5 mol% of Ph_3_PAuCl and [Ru(bpy)_3_](PF_6_)_2_, respectively, in degassed methanol for 2 h at room temperature, we were pleased to observe that the cross-coupled product **3aa** was formed in 46% yield (entry 1). Following a short screen, (see the ESI† for details of additional experiments), DMF was identified as the optimum solvent for this transformation (entry 2). A range of different Au^I^ catalysts bearing various ligands were then evaluated (entries 3–7). Interestingly, the gold(i) chloride complex possessing the comparatively electron-rich phosphine ((*p*-MeO)C_6_H_4_)_3_P led to the highest yield of **3aa** (83%), while the trialkylphosphine Cy_3_P was also tolerated (entry 4). This trend might be related to the stabilization of the Au^III^ center by these strongly donating ligands during the Au^I^/Au^III^ redox cycle. The combination of the Au^I^ complex ((*p*-MeO)C_6_H_4_)_3_PAuCl and the photoredox catalyst [Ru(bpy)_3_](PF_6_)_2_ turned out to be both a highly selective and active C(sp)–H arylating system, as **3aa** could be isolated in 80% yield using very low loadings of [Ru(bpy)_3_](PF_6_)_2_ (0.5 mol%) after a reaction time of only 1 hour and without the formation of the diyne homocoupling product of **1a** (entry 8).^11^ Furthermore, this system does not require the addition of an external base and proceeds under acidic conditions. Interestingly, the reaction also operated efficiently using visible light from a variety of different sources. Under both blue LED (5 W, *λ*_max_ = 465 nm) and even sunlight irradiation, the formation of the cross coupled product **3aa** was observed in good yields (entries 9–10). In addition, the dual gold/photoredox C(sp)–H arylation process showed a remarkable robustness towards oxygen, with **3aa** being obtained in 60% yield under the same conditions (10 mol% ((*p*-MeO)C_6_H_4_)_3_PAuCl and 0.5 mol% [Ru(bpy)_3_](PF_6_)_2_) under an atmosphere of air (entry 11). Upon increasing the loading of [Ru(bpy)_3_](PF_6_)_2_ to 2.5 mol%, the yield of **3aa** recovered to 82% yield, indicating that careful exclusion of oxygen is not required to obtain high yields when a slightly increased loading of the photocatalyst is employed (entry 12). In contrast to our previous studies on alkene oxyarylation, attempts to switch the aryl coupling partner to diaryliodonium salts or use less expensive organic dyes were not successful for this transformation and the combination of aryldiazonium salts and [Ru(bpy)_3_](PF_6_)_2_ was employed for further experiments (entries 13–15).

**Scheme 2:**
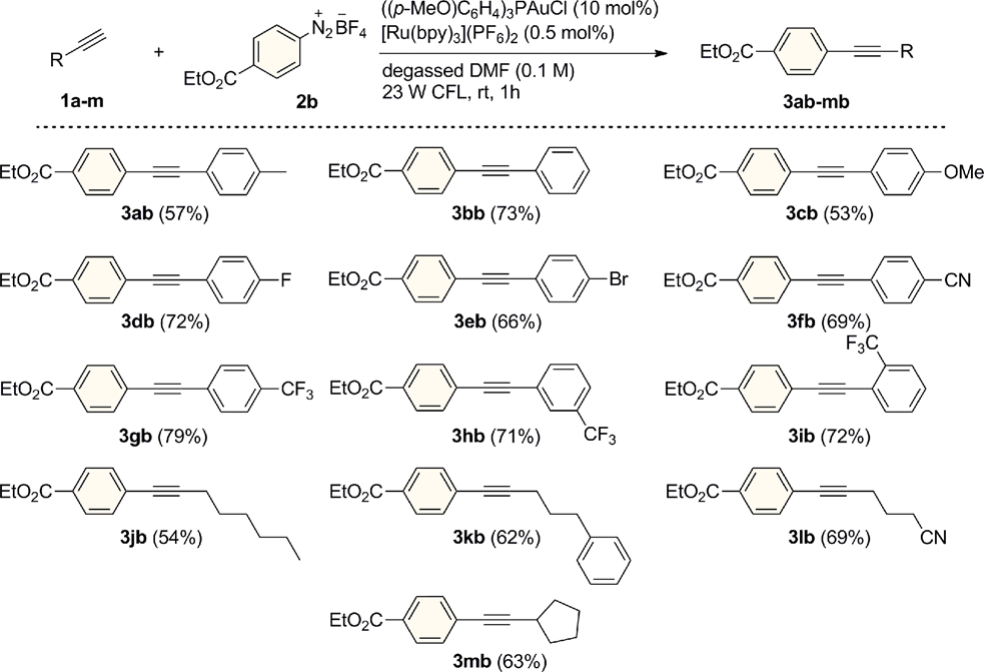
Dual Au/photoredox-catalyzed arylation of different alkynes. General conditions: alkyne (**1a–m**, 0.30 mmol), **2b** (1.2 mmol), [Ru(bpy)_3_](PF_6_)_2_ (0.5 mol%), ((*p*-MeO)C_6_H_4_)_3_PAuCl (10 mol%) and degassed DMF (3.0 mL). Isolated yields.

We next evaluated the reaction scope studying a variety of alkyl and aromatic terminal alkynes using 4-(ethoxycarbonyl)benzenediazonium tetrafluoroborate (**2b**) as the arylating reagent (Scheme 2). With arylalkyne substrates, the reaction was found to tolerate electron-donating and withdrawing substituents on the aryl ring and the corresponding coupled products were isolated in good yields (53–79%) after just 1 h of visible light irradiation. Moreover, as exemplified for the trifluoromethyl-substituted compounds **3gb**, **3hb** and **3ib**, the reaction proceeded with substituents at each of the *para*- *meta*- and *ortho*-positions with seemingly comparable efficiency. Similar good results were obtained for a range of alkyl-substituted alkynes. Derivatives **3jb–mb** were prepared in 54–69% yields with both primary and secondary alkyl groups being tolerated. In all cases, no diyne compounds resulting from homocoupling of the aryl- or alkylalkynes were observed during the reactions.

**Scheme 3:**
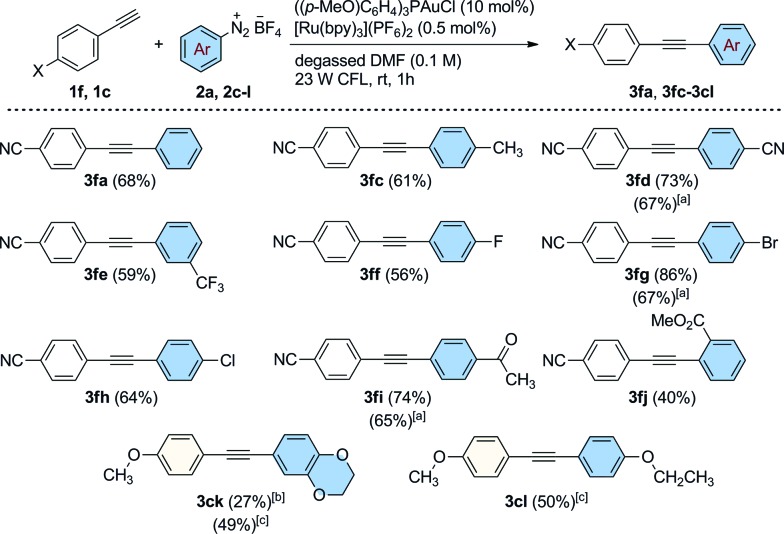
Dual Au/photoredox-catalyzed arylation of **1f** and **1c** with substituted benzenediazonium salts. General conditions: alkyne (0.30 mmol), **2a**, **2c–l** (1.2 mmol), [Ru(bpy)_3_](PF_6_)_2_ (0.5 mol%), ((*p-*MeO)C_6_H_4_)_3_PAuCl (10 mol%) and degassed DMF (3.0 mL). [a] Under air using 2.5 mol% of [Ru(bpy)_3_](PF_6_)_2_ in 1 h. [b] [Ru(bpy)_3_](PF_6_)_2_ (2.5 mol%), 16 h of reaction. [c] [Ru(bpy)_3_](PF_6_)_2_ (2.5 mol%), 5 h under 5 W blue LEDs irradiation. Isolated yields.

Our attention turned next to an investigation of the scope of the reaction towards a range of substituted aryldiazonium salts (**2a**, **2c–l**) using 4-ethynylbenzonitrile **1f** as a standard alkyne coupling partner (Scheme 3). We were pleased to find that aryl sources bearing a variety of electronically diverse functional groups reacted successfully, delivering the corresponding cross-coupled products **3fa**, **3fc–fj** in good yields up to 86%. At this point, the efficiency of the reaction when performed using electron rich groups at both the aromatic alkyne and the diazonium salt was tested by reacting 4-ethynylanisole (**1c**) with 2,3-dihydrobenzo[*b*][1,4]dioxine-6-diazonium tetrafluoroborate (**2k**) under the optimized reaction conditions (Scheme 3). We observed only poor conversions of **1c** unless higher loadings of [Ru(bpy)_3_](PF_6_)_2_ (2.5 mol%) and longer irradiation times (16 h) were used. Under these conditions, the coupled product **3ck** was isolated in 27% yield. Interestingly, the catalytic activity of this system was increased using blue LEDs (5 W, *λ*_max_ = 465 nm) as the light source. After 5 h of irradiation, **3ck** was formed in an improved 49% isolated yield. This new set of reaction conditions was also successfully applied for the synthesis of the coupled product **3cl** (50% isolated yield). The efficiency of the reaction was evaluated for some substrates under an atmosphere of air using a slightly higher loading of the photocatalyst [Ru(bpy)_3_](PF_6_)_2_ (2.5 mol%, conditions from Table 1, entry 12). Interestingly, under these conditions, the arylated products **3fd**, **3fg** and **3fi** were still obtained in good yields (65–67%), demonstrating that efficient catalytic activity can be maintained without rigorous extrusion of oxygen. Sterically-demanding aryldiazonium salts, however, are seemingly less well tolerated under these conditions with the *ortho*-substituted 2-(methoxycarbonyl)benzenediazonium tetrafluoroborate reacting sluggishly to afford the coupled product **3fj** in a moderate yield of 40%.

**Table 1:** Reaction optimization^*a*^

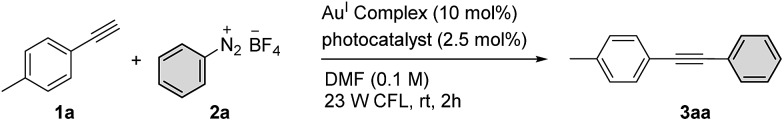			
Entry	Au(i) complex	Photocatalyst	Yield^*b*^ (%)
1^*c*^	Ph_3_PAuCl	[Ru(bpy)_3_](PF_6_)_2_	46
2	Ph_3_PAuCl	[Ru(bpy)_3_](PF_6_)_2_	78
3	((*p-*MeO)C_6_H_4_)_3_PAuCl	[Ru(bpy)_3_](PF_6_)_2_	83
4	Cy_3_PAuCl	[Ru(bpy)_3_](PF_6_)_2_	72
5	XPhosAuCl	[Ru(bpy)_3_](PF_6_)_2_	—
6	(PhO)_3_PAuCl	[Ru(bpy)_3_](PF_6_)_2_	—
7	IPrAuCl	[Ru(bpy)_3_](PF_6_)_2_	—
8^*d*^	((*p-*MeO)C_6_H_4_)_3_PAuCl	[Ru(bpy)_3_](PF_6_)_2_	83 (80)
9^*e*^	((*p-*MeO)C_6_H_4_)_3_PAuCl	[Ru(bpy)_3_](PF_6_)_2_	80
10^*f*^	((*p-*MeO)C_6_H_4_)_3_PAuCl	[Ru(bpy)_3_](PF_6_)_2_	71
11^*g*^	((*p-*MeO)C_6_H_4_)_3_PAuCl	[Ru(bpy)_3_](PF_6_)_2_	60
12^*h*^	((*p-*MeO)C_6_H_4_)_3_PAuCl	[Ru(bpy)_3_](PF_6_)_2_	82
13^*i*^	((*p-*MeO)C_6_H_4_)_3_PAuCl	[Ir(ppy)_2_(dtbbpy)]PF_6_	Trace
14	((*p-*MeO)C_6_H_4_)_3_PAuCl	Eosin Y	—
15	((*p-*MeO)C_6_H_4_)_3_PAuCl	Fluorescein	—

^*a*^General conditions: **1a** (0.10 mmol), **2a** (0.40 mmol), photocatalyst (2.5 mol%), gold complex (10 mol%) and degassed DMF (1.0 mL).

^*b*^NMR yields using CH_2_Br_2_ as internal standard. Isolated yield in parentheses.

^*c*^Degassed methanol (1.0 mL).

^*d*^[Ru(bpy)_3_](PF_6_)_2_ (0.5 mol%), 1 h.

^*e*^[Ru(bpy)_3_](PF_6_)_2_ (0.5 mol%), 5 W blue LEDs, 1 h.

^*f*^[Ru(bpy)_3_](PF_6_)_2_ (0.5 mol%), sunlight, 8 h.

^*g*^[Ru(bpy)_3_](PF_6_)_2_ (0.5 mol%), air, 1 h.

^*h*^[Ru(bpy)_3_](PF_6_)_2_ (2.5 mol%), air, 1 h.

^*i*^With Ph_2_IBF_4_ (0.40 mmol), [Ir(ppy)_2_(dtbbpy)]PF_6_ (5 mol%). See the ESI for further details. ppy = 2-phenylpyridine, dtbbpy = 4,4′-di-*tert*-butyl-2,2′-bipyridine, XPhos = 2-dicyclohexylphosphino-2′,4′,6′-triisopropylbiphenyl, IPr = 1,3-bis(2,6-diisopropylphenyl)-imidazol-2-ylidene.

**Scheme 4:**
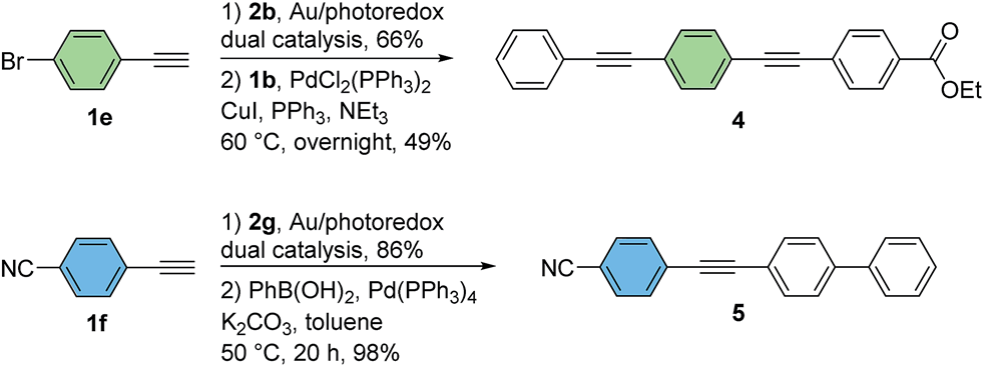
Further manipulation of cross-coupled products (see the ESI† for experimental details).

In addition to its base-free and mild nature, an important feature of the dual gold/photoredox catalytic system is its compatibility with halogenated substrates. In contrast to palladium(0) or many other low-valent late transition metals, gold(i) does not generally undergo conventional oxidative addition to aryl halides under homogeneous conditions.^3^,9d,f,g As such, the brominated diarylalkynes **3eb** and **3fg**, obtained from a bromoarylalkyne and a bromo-substituted aryldiazonium salt respectively, could be isolated in good yields using this method without competitive cleavage of the C–Br bond. As demonstrated by representative Sonogashira–Hagihara and Suzuki–Miyaura coupling reactions in Scheme 4, these substrates can be readily functionalized further using palladium catalysis.^12^ Overall, this two-step protocol is complementary to stepwise palladium-catalyzed coupling sequences, making use of readily-available aryldiazonium salts rather than dihalogenated aryl iodide precursors (Scheme 4).

**Scheme 5:**
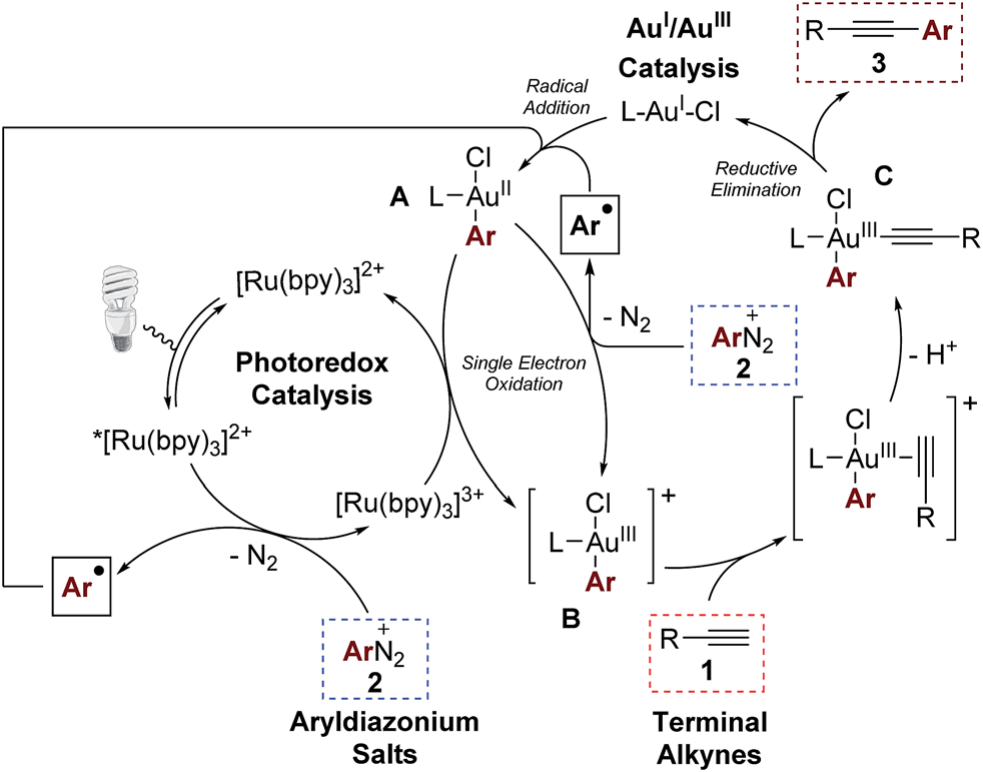
Mechanistic proposal.

In accordance with previous studies on dual Au/photoredox catalysis,^5^,^7^ we envisage a mechanism involving a photoredox-induced homogeneous Au^I^/Au^III^ redox cycle (Scheme 5).^13^ Upon irradiation with visible light, photoexcitation of the [Ru(bpy)_3_]^2+^ photocatalyst takes place to generate the excited form *[Ru(bpy)_3_]^2+^ which then undergoes single electron transfer (SET) with one equivalent of the aryldiazonium salt to deliver an aryl radical and the oxidized species [Ru(bpy)_3_]^3+^.^14^ The photo-generated aryl radical then adds to the Au^I^ catalyst to give the Au^II^ species **A**.^15^ Interestingly, quantum yield measurements at 450 nm by chemical actinometry gave a value of 3.6, reflecting the very important contribution of radical chain processes in this dual catalytic system.^16^ Therefore, we propose that a second SET process between the open-shell species **A** with another equivalent of the aryldiazonium salt mainly contributes to the formation of the electrophilic cationic Au^III^ intermediate **B**. Nevertheless, regeneration of the oxidized [Ru(bpy)_3_]^3+^ to the [Ru(bpy)_3_]^2+^ photoredox catalyst is still feasible by reaction with **A**. The cationic intermediate **B** is expected to be an excellent π-Lewis acid and coordinates the alkyne substrate, activating it towards the formation of the σ-bonded alkynyl-Au^III^ complex **C** upon deprotonation. Intermediate **C** finally undergoes reductive elimination,^17^ regenerating the Au^I^ catalyst and delivering the cross-coupled product.

## Conclusions

In conclusion, we have developed an efficient dual gold/photoredox catalysis methodology for the arylation of terminal alkynes using readily-available aryldiazonium salts. This overall redox neutral cross-coupling process shows broad functional group tolerance, operates at room temperature and is mediated by abundant visible light from a household light bulb or even sunlight. In addition, the base-free nature of the reaction and tolerance of halogenated substrates may be beneficial in the design of cross-coupling sequences. This reaction represents the first application of dual gold/photoredox catalysis for the activation of C–H bonds and further demonstrates the potential of this methodology to access Au^I^/Au^III^ redox cycles under mild conditions without the need for external oxidants.

## Supplementary Material

Supplementary information
