# Supplementary material for: Dual gold/photoredox-catalyzed C(sp)–H arylation of terminal alkynes with diazonium salts
Source: Chem Sci. 2015 Oct 8;7(1):89–93. doi: 10.1039/c5sc02583d (PMC5950757; doi:10.1039/c5sc02583d)
Supplement: Supplementary file 1 [file SC-007-C5SC02583D-s001.pdf]

Electronic Supplementary Material (ESI) for Chemical Science.
This journal is © The Royal Society of Chemistry 2015

Supporting Information

Dual Gold/Photoredox-Catalyzed C(sp)-H Arylation of Terminal Alkynes with Diazonium Salts

Adrian Tlahuext Aca, Matthew N. Hopkinson, Basudev Sahoo and Frank Glorius

Westfälische Wilhelms-Universität Münster, NRW Graduate School of Chemistry, Organisch-Chemisches Institut, Corrensstrasse 40, 48149 Münster, Germany

glorius@uni-muenster.de

Contents

1. General Information
2. Optimization and Control Reactions
    2.1 Optimization Table
    2.2 Control Reactions
3. Scope and Limitations Studies
    3.1 General Procedure for the Cross-Coupling Reactions
    3.2 Characterization Data of Cross-Coupled Products
4. Further Manipulations of Brominated Diarylalkynes
5. Kinetic Profile
6. Quantum Yield Measurements
7. References
8. NMR Spectra of Cross-Coupled Products

1. General Information

Unless otherwise noted, all reactions were carried out under an atmosphere of argon in flame-dried glassware. The solvents used were purified by distillation over standard drying agents and were stored over molecular sieves or transferred under argon. Visible light from compact fluorescent light bulbs (CFL) was provided by a standard household desk lamp purchased from Massive fitted with a 23 W fluorescent light bulb, which was situated around 10 cm from the reaction vessel. Blue LEDs (5 W, λ = 465 nm) purchased from Kruse Lighting Solutions (HPB8b-48K5BF/WPCB) were used for blue light irradiation. A custom made "light box" was used with 6 blue LEDs arranged around the reaction vessels at a distance of around 5 cm (see Figure S1 below). A fan attached to the apparatus was used to maintain the temperature inside the "box" at no more than 9 °C above room temperature. The reaction was also performed under sunlight on the roof of the institute (see Figure S2 below).

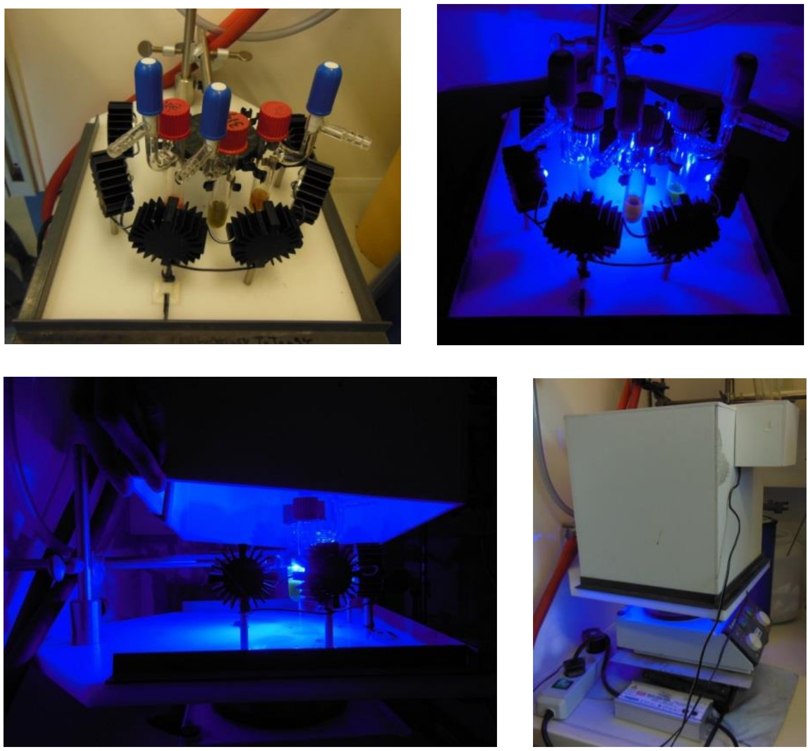

Figure S1 Photographs of the custom-made "light box" used for reactions conducted under blue LED irradiation.

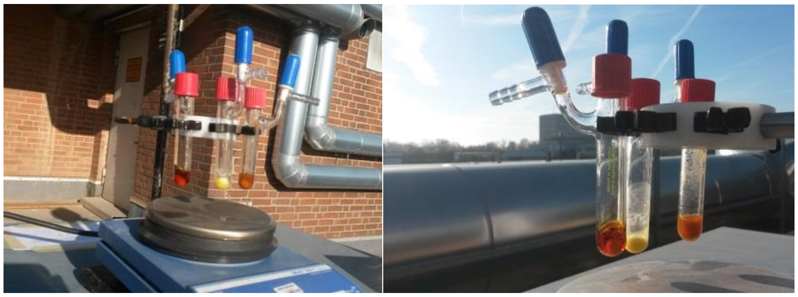

Figure S2 Photographs of the set up for reactions conducted under sunlight.

Aryldiazonium salts **2a-l** were prepared according the procedure of Hanson.[1] Alkyne substrates were commercially available (**1a-m**) and used as received. $[Ru(bpy)_3]_2(PF_6)_2$ (bpy = 2,2'-bipyridine)[2] and $[Ir(ppy)_2(dtbbpy)](PF_6)$ (ppy = 2-phenylpyridine, dtbbpy = 4,4'-di-*tert*-butyl-2,2'-bipyridine)[3] were prepared according to literature procedures. The gold(I) chloride complexes $((p\text{-MeO})C_6H_4)_3PAuCl$, $(C_6F_5)_3PAuCl$, XPhosAuCl (XPhos =2-dicyclohexylphosphino-2',4',6'-triisopropylbiphenyl), $(PhO)_3PAuCl$ and Cy_3PAuCl were prepared by reacting an equimolar ratio of the appropriate phosphine with (tht)AuCl (tht = tetrahydrothiophene) in dry dichloromethane in a method analogous to that of Hashmi and co-workers.[4] IPrAuCl (IPr = 1,3-bis(2,6-diisopropylphenyl)-imidazol-2-ylidene) was prepared according to the procedure of Nolan.[5] $[Ph_3PAu]NTf_2$ was prepared by reacting Ph_3PAuCl with an equimolar amount of $AgNTf_2$ in dichloromethane in a procedure analogous to that of Gagosz and co-workers.[6] All other gold catalysts and photoredox catalysts were commercially available and used as received.

Flash chromatography was performed on Merck silica gel (40-63 mesh) using standard techniques.

NMR-spectra were recorded on a Bruker ARX-300, AV-300, AV-400 MHz or on a Varian Associated, Varian 600 unity plus spectrometer. Chemicals shifts (δ) are quoted in ppm downfield of tetramethylsilane. The residual solvent signals were used as references for ^{1}H and ^{13}C NMR spectra ($CDCl_3$: δ_H = 7.26 ppm, δ_C = 77.13 ppm). ^{19}F NMR spectra are not calibrated by an internal reference. Coupling constants (*J*) are quoted in Hz.

GC-MS spectra were recorded on an Agilent Technologies 7890A GC-system with an Agilent 5975C VL MSD or an Agilent 5975 inert Mass Selective Detector (EI) and a HP-5MS column (0.25 mm x 30 m, film: 0.25 µm). The major signals are quoted in *m*/*z* with the relative intensity in parentheses. The method indicated as ‘50_40’ starts with the injection temperature T_0 (50 °C); after holding this temperature for 3 min, the column is heated by 40 °C/min to temperature T_1 (290 °C or 320 °C) and this temperature is held for an additional time t. ESI mass spectra were recorded on a Bruker Daltonics MicroTof spectrometer. Infrared spectra were recorded on a Varian Associates FT-IR 3100 Excalibur or on a Shimadzu FTIR 8400S spectrometer. The wave numbers (ν) of recorded IR-signals are quoted in cm^{-1}.

2. Optimization and Control Reactions

2.1 Optimization Table[a]

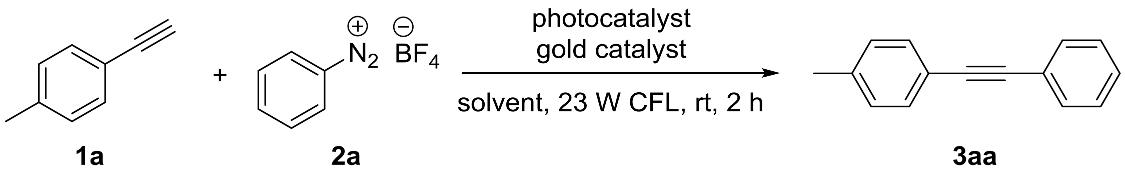

Entry	Photocatalyst (mol%)	Metal Complex (mol%)	Solvent	Yield[b] of 3aa
**1**	$[Ru(bpy)_3](PF_6)_2$ (2.5)	Ph_3PAuCl (10)	MeOH	46
**2**	$[Ru(bpy)_3](PF_6)_2$ (2.5)	Ph_3PAuCl (10)	EtOH	7
**3**	$[Ru(bpy)_3](PF_6)_2$ (2.5)	Ph_3PAuCl (10)	^{i}PrOH	-
**4**	$[Ru(bpy)_3](PF_6)_2$ (2.5)	Ph_3PAuCl (10)	EtOAc	-
**5**	$[Ru(bpy)_3](PF_6)_2$ (2.5)	Ph_3PAuCl (10)	Me_2CO	35
**6**	$[Ru(bpy)_3](PF_6)_2$ (2.5)	Ph_3PAuCl (10)	CH_2Cl_2	-
**7**	$[Ru(bpy)_3](PF_6)_2$ (2.5)	Ph_3PAuCl (10)	Toluene	-
**8**	$[Ru(bpy)_3](PF_6)_2$ (2.5)	Ph_3PAuCl (10)	1,4-Dioxane	-
**9**	$[Ru(bpy)_3](PF_6)_2$ (2.5)	Ph_3PAuCl (10)	DMSO	50
**10**	$[Ru(bpy)_3](PF_6)_2$ (2.5)	Ph_3PAuCl (10)	DMF	78
**11**	$[Ru(bpy)_3](PF_6)_2$ (2.5)	$((p\text{-MeO})C_6H_4)_3PAuCl$ (10)	DMF	83
**12**	$[Ru(bpy)_3](PF_6)_2$ (2.5)	XPhosAuCl (10)	DMF	-
**13**	$[Ru(bpy)_3](PF_6)_2$ (2.5)	Cy_3PAuCl (10)	DMF	72
**14**	$[Ru(bpy)_3](PF_6)_2$ (2.5)	$(C_6F_5)_3PAuCl$ (10)	DMF	77
**15**	$[Ru(bpy)_3](PF_6)_2$ (2.5)	(tht)AuCl (10)	DMF	70
**16**	$[Ru(bpy)_3](PF_6)_2$ (2.5)	$(PhO)_3PAuCl$ (10)	DMF	-
**17**	$[Ru(bpy)_3](PF_6)_2$ (2.5)	$[Ph_3PAu]NTf_2$ (10)	DMF	77
**18**	$[Ru(bpy)_3](PF_6)_2$ (2.5)	IPrAuCl (10)	DMF	-
**19**	$[Ru(bpy)_3](PF_6)_2$ (2.5)	AuCl (10)	DMF	68
**20**	$[Ru(bpy)_3](PF_6)_2$ (2.5)	$AuCl_3$ (10)	DMF	27
**21**	$[Ru(bpy)_3](PF_6)_2$ (2.5)	$((p\text{-MeO})C_6H_4)_3PAuCl$ (5)	DMF	61
**22**[c]	$[Ru(bpy)_3](PF_6)_2$ (2.5)	$((p\text{-MeO})C_6H_4)_3PAuCl$ (10)	DMF	76
**23**[d]	$[Ru(bpy)_3](PF_6)_2$ (0.5)	$((p\text{-MeO})C_6H_4)_3PAuCl$ (10)	DMF	83 (80)
**24**[e]	$[Ru(bpy)_3](PF_6)_2$ (0.5)	$((p\text{-MeO})C_6H_4)_3PAuCl$ (10)	DMF	80
**25**[f]	$[Ru(bpy)_3](PF_6)_2$ (0.5)	$((p\text{-MeO})C_6H_4)_3PAuCl$ (10)	DMF	71
**26**[g]	$[Ru(bpy)_3](PF_6)_2$ (0.5)	$((p\text{-MeO})C_6H_4)_3PAuCl$ (10)	DMF	60
**27**[g]	$[Ru(bpy)_3](PF_6)_2$ (2.5)	$((p\text{-MeO})C_6H_4)_3PAuCl$ (10)	DMF	82
**28**[d]	$[Ir(ppy)_2(dtbbpy)](PF_6)_2$ (0.5)	$((p\text{-MeO})C_6H_4)_3PAuCl$ (10)	DMF	trace
**29**[d]	Eosin Y (0.5)	$((p\text{-MeO})C_6H_4)_3PAuCl$ (10)	DMF	trace
**30**[d]	Rose Bengal (0.5)	$((p\text{-MeO})C_6H_4)_3PAuCl$ (10)	DMF	-
**31**[d]	Rhodamine B (0.5)	$((p\text{-MeO})C_6H_4)_3PAuCl$ (10)	DMF	-
**32**[d]	Fluorescein (0.5)	$((p\text{-MeO})C_6H_4)_3PAuCl$ (10)	DMF	trace
**33**	$[Ru(bpy)_3]Cl_2\cdot H_2O$ (0.5)	$((p\text{-MeO})C_6H_4)_3PAuCl$ (10)	DMF	80

[a] General conditions: Alkyne **1a** (0.1 mmol), diazonium salt **2a** (0.4 mmol), photocatalyst, gold catalyst and the solvent (1 ml) were added to a flame-dried Schlenk flask in the absence of light. The mixture was degassed with three freeze-pump-thaw cycles, flushed with argon, sealed and stirred at room temperature under visible light irradiation (23

W CFL) for 2 h. [b] NMR yields using CH_2Br_2 as internal standard. [c] 2 equivalents of **2a**. [d] 1 h reaction time. [e] Blue LEDs, 1 h. [f] Sunlight, 8 h. [g] Under Air, 1 h. Isolated yield in parentheses. bpy = 2,2′-bipyridine, ppy = 2-phenylpyridine, dtbbpy = 4,4'-di-*tert*-butyl-2,2'-bipyridine, XPhos = 2-dicyclohexylphosphino-2',4',6'-triisopropylbiphenyl, Cy = cyclohexyl, tht = tetrahydrothiophene, IPr = 1,3-bis(2,6-diisopropylphenyl)-imidazol-2-ylidene, DMSO = dimethylsulfoxide, DMF = *N*,*N*-dimethylformamide. The structures of Eosin Y, Rose Bengal, Rhodamine B and Fluorescein are shown below.

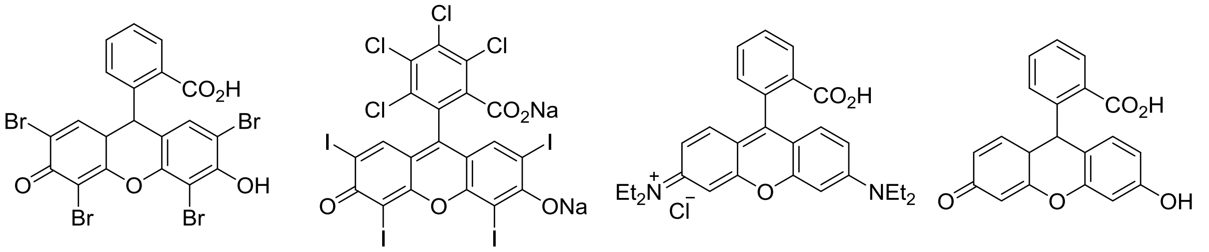

Eosin Y   Rose Bengal   Rhodamine B   Fluorescein

2.2 Control Reactions

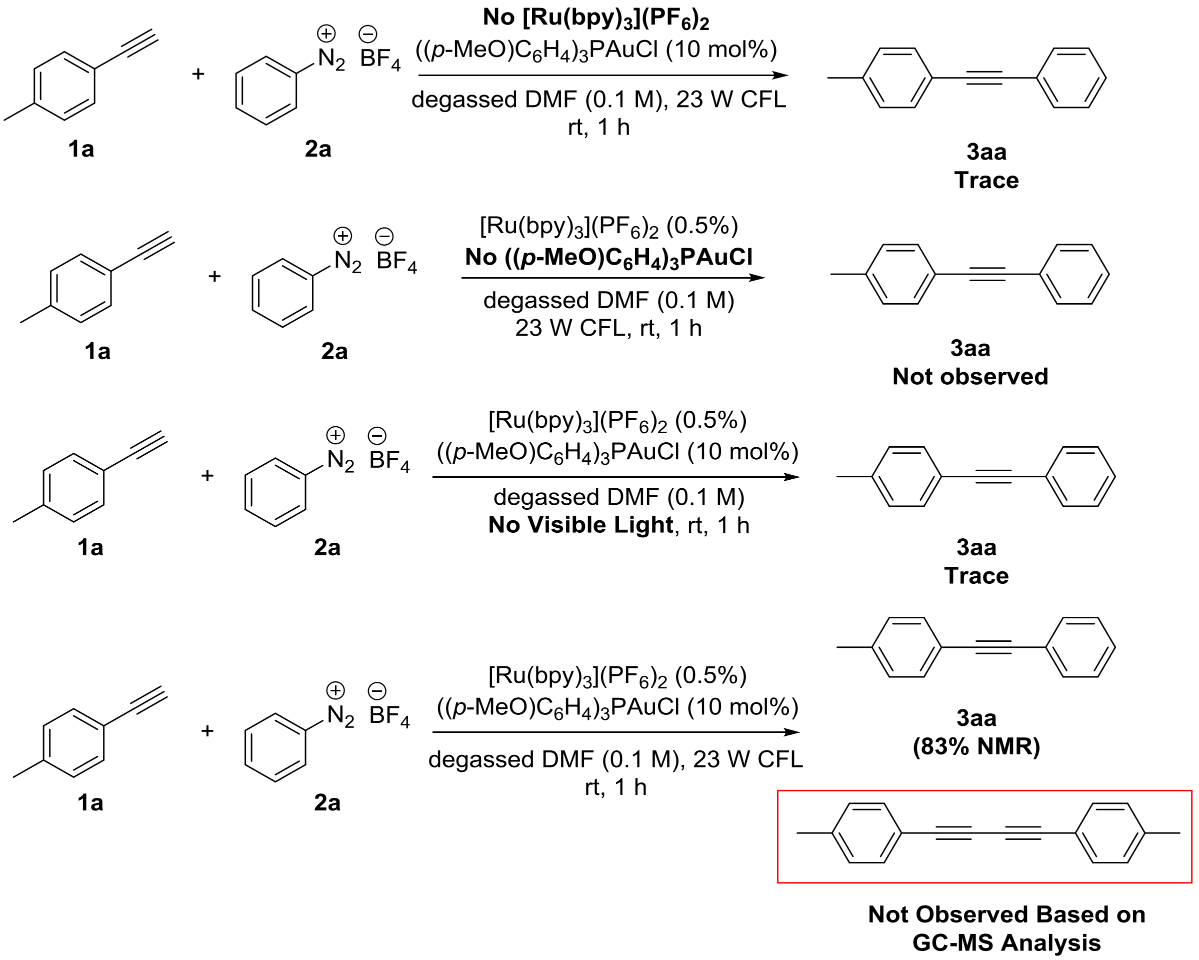

Detection of homocoupling product derived from **1a** by GC-MS analysis.[7]

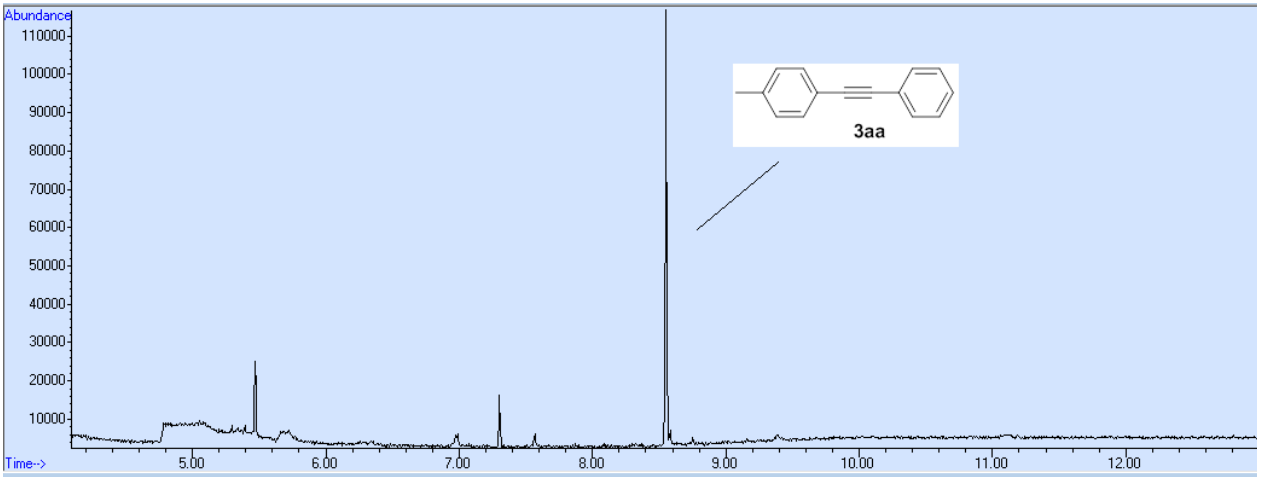

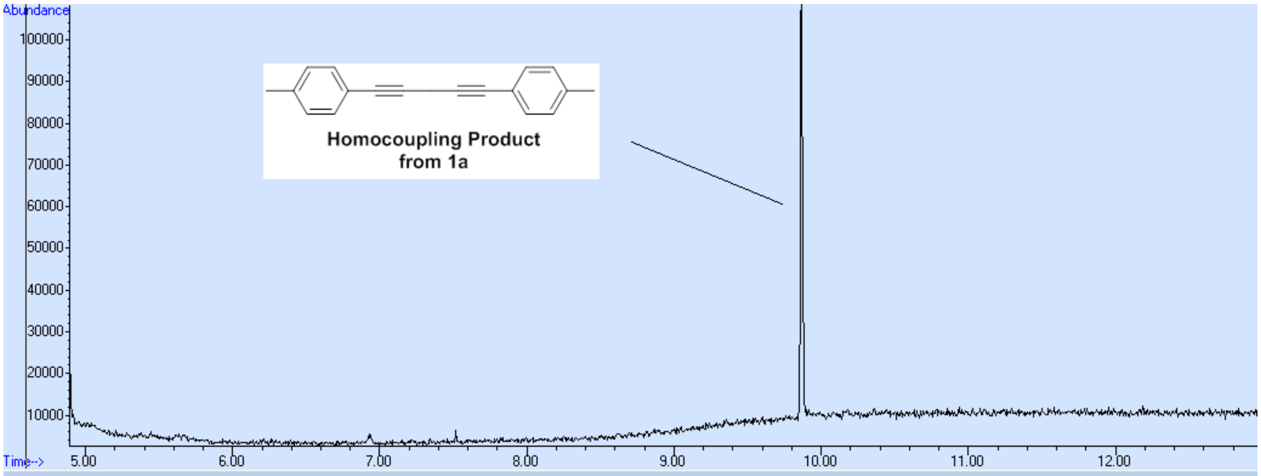

3. Scope and Limitations Studies

3.1 General Procedure for the Cross-Coupling Reactions

$[Ru(bpy)_3](PF_6)_2$ (1.2 mg, 1.5 μmol, 0.5 mol%), $((p\text{-MeO})C_6H_4)_3PAuCl$ (17.4 mg, 29.7 μmol, 10 mol%), the aryldiazonium salt **2** (1.2 mmol, 4.0 equiv) and the alkyne substrate **1** (0.3 mmol, 1.0 equiv) were added to a flame-dried Schlenk flask containing a stirring bar. In the absence of light, anhydrous DMF (3.0 mL, 0.10 M) was added and the mixture was degassed using three freeze-pump-thaw cycles. The flask was then flushed with argon, sealed and the mixture was stirred under irradiation from a desk lamp fitted with a 23 W fluorescent light bulb (CFL). After 1 h, the mixture was quenched with a saturated solution of $NaHCO_3$ (3 mL) and distilled water (2 mL) was added. The crude reaction mixture was then extracted with ethyl acetate (4 × 5 mL) and the combined organic fractions were dried over anhydrous sodium sulfate, filtered and concentrated under vacuum. The crude products **3** were purified by column chromatography over silica gel.

3.2 Characterization Data of Cross-Coupled Products

1-Methyl-4-(phenylethynyl)benzene (3aa)[8]

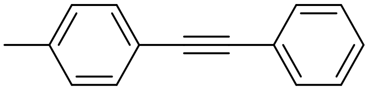

3aa

Prepared from 4-ethynyltoluene (**1a**) and phenyldiazonium tetrafluoroborate (**2a**) on a 0.30 mmol scale. Purified by column chromatography on silica gel (eluent = Pentane). White solid (46 mg, 0.24 mmol, 80%).

^{1}H NMR (300 MHz, $CDCl_3$): δ (ppm): 2.38 (s, 3H), 7.15-7.18 (m, 2H), 7.32-7.37 (m, 3H), 7.43-7.46 (m, 2H), 7.52-7.55 (m, 2H). **^{13}C NMR (75.5 MHz, $CDCl_3$):** δ (ppm): 21.8 (CH_3), 89.0 (C_q), 89.8 (C_q), 120.5 (C_q), 123.7 (C_q), 128.4 (CH), 128.6 (CH), 129.4 (CH), 131.83 (CH), 131.88 (CH), 138.72 (C_q). **R_f (pentane):** 0.45. **GC-MS: t_R (50_40):** 8.5 min. **EI-MS:** *m/z* (%): 193 (15), 192 (100), 191 (47), 190 (11), 189 (22), 165 (14), 115 (6), 94 (6). **IR (ATR):** ν (cm^{-1}): 2361, 2340, 1735, 1717, 1699, 1652, 1559, 1541, 1508, 1458, 817, 755, 689, 669, 566, 540, 529.

Ethyl 4-(*p*-tolylethynyl)benzoate (3ab)[8]

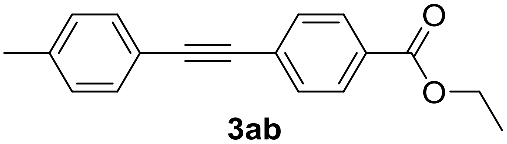

3ab

Prepared from *p*-tolylacetylene (**1a**) and 4-(ethoxycarbonyl)benzenediazonium tetrafluoroborate (**2b**) on a 0.30 mmol scale. Purified by column chromatography on silica gel (eluent = pentane to pentane:EtOAc, 50:1). Isolated as a mixture with ethyl benzoate side-product, which was subsequently removed by drying under high vacuum (< 1 mbar) overnight. White solid (45 mg, 0.17 mmol, 57%).

^{1}H NMR (300 MHz, $CDCl_3$): δ (ppm): 1.40 (t, $^3J_{H,H}$ = 7.1 Hz, 3H), 3.84 (s, 3H), 4.38 (q, $^3J_{H,H}$ = 7.1 Hz, 2H), 6.89 (dm, $^3J_{H,H}$ = 8.8 Hz, 2H), 7.49 (dm, $^3J_{H,H}$ = 8.8 Hz, 2H), 7.56 (dm, $^3J_{H,H}$ = 8.6 Hz, 2H), 8.01 (dm, $^3J_{H,H}$ = 8.6 Hz, 2H). **^{13}C NMR (75.5 MHz, $CDCl_3$):** δ (ppm): 14.4 (CH_3), 21.7 (CH_3), 61.2 (CH_2), 88.2 (C_q), 92.7 (C_q), 119.7 (C_q), 128.2 (C_q), 129.3 (CH), 129.6 (CH), 129.7 (C_q), 131.5 (CH), 131.7 (CH), 139.1 (C_q), 166.2 (C_q). **R_f (pentane:EtOAc 20:1):** 0.57. **GC-MS: t_R (50_40):** 10.0 min. **EI-MS:** *m/z* (%): 265 (19), 264 (100), 236 (25), 220 (16), 219 (81), 191 (15), 190 (15), 189 (40), 176 (13), 165 (8), 163 (7), 96 (5), 95 (8), 82 (6), 43 (6). **HR-MS (ESI):** m/z calculated for $[C_{18}H_{16}O_2Na]^+$ ($[M+Na]^+$): 287.1043, measured: 287.1041. **IR (ATR):** ν (cm^{-1}): 3031, 3001, 2957, 2919, 2854, 2218, 1700, 1599, 1557, 1517, 1446, 1405, 1366, 1306, 1269, 1171, 1138, 1102, 1015, 860, 821, 769, 696, 645, 600, 541.

Ethyl 4-(phenylethynyl)benzoate (3bb)[9]

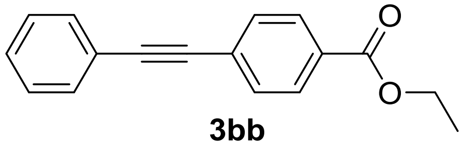

3bb

Prepared from phenylacetylene (**1b**) and 4-(ethoxycarbonyl)benzenediazonium tetrafluoroborate (**2b**) on a 0.30 mmol scale. Purified by column chromatography on silica gel (eluent = pentane to pentane:EtOAc, 50:1). Isolated as a mixture with ethyl benzoate side-product, which was subsequently removed by drying under high vacuum (< 1 mbar) overnight. White solid (55 mg, 0.22 mmol, 73%).

^{1}H NMR (300 MHz, $CDCl_3$): δ (ppm): 1.41 (t, $^3J_{H,H}$ = 7.1 Hz, 3H), 4.39 (q, $^3J_{H,H}$ = 7.1 Hz, 2H), 7.32−7.42 (m, 3H), 7.51−7.64 (m, 4H), 8.03 (dm, $^3J_{H,H}$ = 8.6 Hz, 2H). **^{13}C NMR (75.5 MHz, $CDCl_3$):** δ (ppm): 14.5 (CH_3), 61.3 (CH_2), 88.8 (C_q), 92.4 (C_q), 122.8 (C_q), 128.0 (C_q), 128.6 (CH), 128.9 (CH), 129.6 (CH), 129.9 (C_q), 131.6 (CH), 131.8 (CH), 166.2 (C_q). **R_f (pentane:EtOAc 20:1):** 0.57. **GC-MS: t_R (50_40):** 9.6 min. **EI-MS:** *m/z* (%): 251 (16), 250 (86), 223 (5), 222 (33), 206 (19), 205 (100), 178 (6), 177 (20), 176 (54), 151 (21), 147 (5), 126 (5), 103 (7), 88 (16), 75 (5). **HR-MS (ESI):** m/z calculated for $[C_{17}H_{14}O_2Na]^+$ ($[M+Na]^+$): 273.0886, measured: 273.0884. **IR (ATR):** ν (cm^{-1}): 3068, 2995, 2941, 2216, 1698, 1633, 1605, 1551, 1484, 1442, 1406, 1368, 1308, 1273, 1174, 1141, 1125, 1102, 1072, 1020, 919, 860, 772, 757, 691, 602, 585.

Ethyl 4-((4-methoxyphenyl)ethynyl)benzoate (3cb)[10]

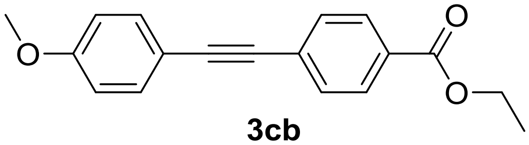

3cb

Prepared from 4-ethynylanisole (**1c**) and 4-(ethoxycarbonyl)benzenediazonium tetrafluoroborate (**2b**) on a 0.30 mmol scale. Purified by column chromatography on silica gel (eluent = pentane:EtOAc, 20:1). White solid (43 mg, 0.15 mmol, 51%).

^{1}H NMR (300 MHz, $CDCl_3$): δ (ppm): 1.40 (t, $^3J_{H,H}$ = 7.1 Hz, 3H), 2.38 (s, 3H), 4.39 (q, $^3J_{H,H}$ = 7.1 Hz, 2H), 7.19 (dm, $^3J_{H,H}$ = 8.0 Hz, 2H), 7.44 (dm, $^3J_{H,H}$ = 8.0 Hz, 2H), 7.58 (dm, $^3J_{H,H}$ = 8.6 Hz, 2H), 8.02 (dm, $^3J_{H,H}$ = 8.6 Hz, 2H). **^{13}C NMR (75.5 MHz, $CDCl_3$):** δ (ppm): 14.4 (CH_3), 55.5 (CH_3), 61.2 (CH_2), 87.7 (C_q), 92.6 (C_q), 114.2 (CH), 114.9 (C_q), 128.4 (C_q), 129.6 (CH), 131.4 (CH), 133.4 (CH), 160.1 (C_q), 166.3 (C_q); *Note: One quaternary carbon peak is not resolved due to overlapping*. **R_f (pentane:EtOAc 20:1):** 0.37. **GC-MS: t_R (50_40):** 10.5 mi. **EI-MS:** *m/z* (%): 281 (20), 280 (100), 265 (6), 252 (23), 237 (7), 235 (38), 209 (7), 207 (7), 176 (6), 164 (21), 163 (23) 118 (6). **HR-MS (ESI):** m/z calculated for $[C_{18}H_{16}O_3Na]^+$ ($[M+Na]^+$): 303.0992, measured: 303.1007. **IR (ATR):** ν (cm^{-1}): 3044, 2846, 2213, 1703, 1598, 1568, 1516, 1502, 1469, 1404, 1365, 1308, 1274, 1246, 1173, 1138, 1124, 1106, 1022, 877, 829, 765, 732, 693, 645, 610, 595, 564, 540.

Ethyl 4-((4-fluorophenyl)ethynyl)benzoate (3db)

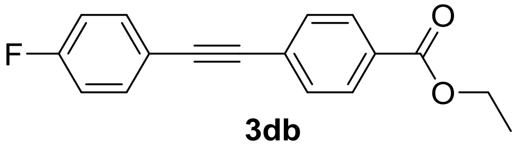

3db

Prepared from 1-ethynyl-4-fluorobenzene (**1d**) and 4-(ethoxycarbonyl)benzenediazonium tetrafluoroborate (**2b**) on a 0.30 mmol scale. Purified by column chromatography on silica gel (eluent = pentane to pentane:EtOAc, 100:1). Isolated as a mixture with ethyl benzoate side-product, which was subsequently removed by drying under high vacuum (< 1 mbar) overnight. White solid (58 mg, 0.22 mmol, 72%).

^{1}H NMR (400 MHz, $CDCl_3$): δ (ppm): 1.40 (t, $^{3}J_{H,H}$ = 7.1 Hz, 3H), 4.39 (q, $^{3}J_{H,H}$ = 7.1 Hz, 2H), 7.06 (ddm, $^{3}J_{H,H}$ = 8.6 Hz, $^{3}J_{H,F}$ = 8.6 Hz, 2H), 7.48 – 7.59 (m, 4H), 8.03 (dm, $^{3}J_{H,H}$ = 8.6 Hz, 2H). **^{13}C NMR (101 MHz, $CDCl_3$):** δ (ppm): 14.5 (CH_3), 61.3 (CH_2), 88.5 (C_q), 91.3 (C_q), 115.9 (d, $^{2}J_{C,F}$ = 22 Hz, CH), 119.0 (d, $^{4}J_{C,F}$ = 4 Hz, C_q), 127.8 (C_q), 129.6 (CH), 130.0 (C_q), 131.5 (CH), 133.8 (d, $^{3}J_{C,F}$ = 8 Hz, CH), 163.9 (*d*, $^{1}J_{C,F}$ = 250 Hz, C_q), 166.2 (C_q). **^{19}F NMR (282 MHz, $CDCl_3$):** δ (ppm): −110.1 (tt, $^{3}J_{H,F}$ = 8.6 Hz, $^{4}J_{H,F}$ = 5.4 Hz). **R_f (pentane:EtOAc 20:1):** 0.55. **GC-MS: t_R (50_40):** 9.5 min. **EI-MS:** *m/z* (%): 269 (16), 268 (88), 240 (26), 224 (18), 223 (100), 195 (17), 194 (37), 175 (11), 169 (8), 168 (5), 112 (6), 97 (7). **HR-MS (ESI):** m/z calculated for $[C_{17}H_{13}FO_2Na]^+$ ($[M+Na]^+$): 291.0792, measured: 291.0793. **IR (ATR):** ν (cm^{-1}): 3064, 2995, 2933, 2221, 1900, 1705, 1597, 1563, 1515, 1502, 1478, 1452, 1406, 1368, 1208, 1273, 1233, 1174, 1141, 1121, 1097, 1016, 856, 833, 795, 766, 690, 581.

Ethyl 4-((4-bromophenyl)ethynyl)benzoate (3eb)[11]

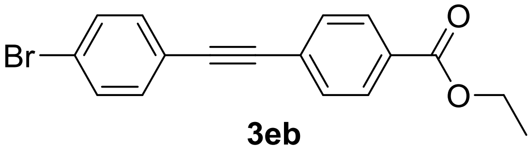

3eb

Prepared from 1-bromo-4-ethynylbenzene (**1e**) and 4-(ethoxycarbonyl)benzenediazonium tetrafluoroborate (**2b**) on a 0.30 mmol scale. Purified by column chromatography on silica gel (eluent = pentane to pentane:EtOAc, 50:1). Isolated as a mixture with ethyl benzoate side-product, which was subsequently removed by drying under high vacuum (< 1 mbar) overnight. White solid (65 mg, 0.20 mmol, 66%).

^{1}H NMR (300 MHz, $CDCl_3$): δ (ppm): 1.40 (t, $^{3}J_{H,H}$ = 7.1 Hz, 3H), 4.39 (q, $^{3}J_{H,H}$ = 7.1 Hz, 2H), 7.40 (dm, $^{3}J_{H,H}$ = 8.5 Hz, 2H), 7.49 (dm, $^{3}J_{H,H}$ = 8.5 Hz, 2H), 7.57 (dm, $^{3}J_{H,H}$ = 8.5 Hz, 2H), 8.02 (dm, $^{3}J_{H,H}$ = 8.5 Hz, 2H). **^{13}C NMR (75.5 MHz, $CDCl_3$):** δ (ppm): 14.5 (CH_3), 61.3 (CH_2), 89.9 (C_q), 91.2 (C_q), 121.8 (C_q), 123.2 (C_q), 127.6 (C_q), 129.6 (CH), 130.2 (C_q), 131.6 (CH), 131.8 (CH), 133.2 (CH), 166.1 (C_q). **R_f (pentane:EtOAc 20:1):** 0.61. **GC-MS: t_R (50_40):** 10.5 min. **EI-MS:** *m/z* (%): 331 (17), 330 (96), 329 (23), 328 (100), 303 (5), 302 (30), 301 (6), 300 (32), 286 (17), 285 (87), 284 (18), 283 (89), 257 (9), 255 (9), 207 (5), 205 (6), 204 (6), 177 (19), 176 (100), 175 (17), 174 (12), 151 (7), 150 (25), 149 (9), 143 (7), 141 (7), 128 (5), 127 (7), 126 (8), 125 (5), 99 (8), 98 (5), 88 (17), 87 (8), 75 (11), 74 (9), 51 (6). **HR-MS (ESI):** m/z calculated for $[C_{17}H_{13}{}^{79}BrO_2Na]^+$ ($[M+Na]^+$): 350.9991, measured: 350.9993; m/z calculated for $[C_{17}H_{13}{}^{81}BrO_2Na]^+$ ($[M+Na]^+$): 352.9971,

measured: 352.9973. **IR (ATR):** ν (cm^{-1}): 3002, 2851, 2216, 1907, 1712, 1653, 1604, 1582, 1561, 1509, 1480, 1404, 1389, 1368, 1307, 1268, 1162, 1138, 1097, 1068, 1005, 859, 821, 783, 766, 728, 694, 637, 620, 591, 567, 544.

Ethyl 4-((4-cyanophenyl)ethynyl)benzoate (3fb)[8]

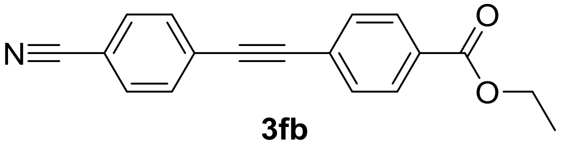

3fb

Prepared from 1-cyano-4-ethynylbenzene (**1f**) and 4-(ethoxycarbonyl)benzenediazonium tetrafluoroborate (**2b**) on a 0.30 mmol scale. Purified by column chromatography on silica gel (eluent = pentane:EtOAc, 20:1). Orange solid (57 mg, 0.21 mmol, 69%).

^{1}H NMR (300 MHz, CDCl$_3$): δ (ppm): 1.40 (t, $^3J_{H,H}$ = 7.1 Hz, 3H), 4.39 (q, $^3J_{H,H}$ = 7.1 Hz, 2H), 7.55 – 7.68 (m, 6H), 8.05 (dm, $^3J_{H,H}$ = 8.5 Hz, 2H). **^{13}C NMR (75.5 MHz, CDCl$_3$):** δ (ppm): 14.4 (CH_3), 61.4 (CH_2), 90.3 (C_q), 92.9 (C_q), 112.1 (C_q), 118.5 (C_q), 126.8 (C_q), 127.7 (C_q), 129.7 (CH), 130.7 (C_q), 131.8 (CH), 132.2 (CH), 132.3 (CH), 166.0 (C_q). **R_f (pentane:EtOAc 20:1):** 0.27. **GC-MS: t_R (50_40):** 10.6 min. **EI-MS:** *m/z* (%): 276 (13), 275 (69), 248 (7), 247 (41), 231 (24), 230 (100), 230 (5), 203 (5), 202 (19), 201 (36), 176 (16), 175 (22), 174 (5), 151 (6), 149 (6), 101 (7), 87 (5). **HR-MS (ESI):** m/z calculated for $[C_{18}H_{13}NO_2Na]^+$ ($[M+Na]^+$): 298.0838, measured: 298.0841. **IR (ATR):** ν (cm^{-1}): 3046, 2991, 2908, 2226, 1936, 1705, 1605, 1561, 1515, 1496, 1477, 1405, 1365, 1307, 1273, 1173, 1139, 1122, 1097, 1020, 875, 859, 839, 767, 693, 641, 606, 596, 556, 528.

Ethyl 4-((4-(trifluoromethyl)phenyl)ethynyl)benzoate (3gb)[12]

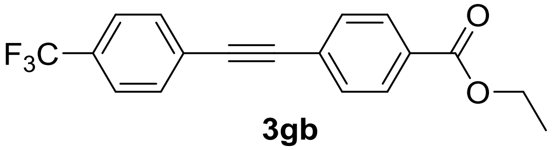

3gb

Prepared from 1-ethynyl-4-(trifluoromethyl)benzene (**1g**) and 4-(ethoxycarbonyl)benzenediazonium tetrafluoroborate (**2b**) on a 0.30 mmol scale. Purified by column chromatography on silica gel (eluent = pentane to pentane:EtOAc, 50:1). Isolated as a mixture with ethyl benzoate side-product, which was subsequently removed by drying under high vacuum (< 1 mbar) overnight. White solid (75 mg, 0.24 mmol, 79%).

^{1}H NMR (300 MHz, CDCl$_3$): δ (ppm): 1.40 (t, $^3J_{H,H}$ = 7.1 Hz, 3H), 4.39 (q, $^3J_{H,H}$ = 7.1 Hz, 2H), 7.57 – 7.68 (m, 6H), 8.05 (dm, $^3J_{H,H}$ = 8.5 Hz, 2H). **^{13}C NMR (75.5 MHz, CDCl$_3$):** δ (ppm): 14.4 (CH_3), 61.3 (CH_2), 90.7 (C_q), 91.0 (C_q), 124.0 (q, $^1J_{C,F}$ = 272 Hz, C_q), 125.5 (q, $^3J_{C,F}$ = 4 Hz, CH), 126.7 (q, $^5J_{C,F}$ = 1 Hz, C_q), 127.2

(C_q), 129.8 (CH), 130.5 (q, $^2J_{C,F}$ = 33 Hz, C_q), 130.5 (C_q), 131.7 (CH), 132.1 (CH), 166.1 (C_q). **^{19}F NMR (282 MHz, $CDCl_3$):** δ (ppm): −62.9 (s). **R_f (pentane:EtOAc 20:1):** 0.57. **GC-MS: t_R (50_40):** 9.4 min. **EI-MS:** *m/z* (%): 319 (13), 318 (61), 299 (5), 290 (28), 274 (18), 273 (100), 245 (11), 225 (17), 176 (23), 175 (5), 136 (5). **HR-MS (ESI):** m/z calculated for $[C_{18}H_{13}F_3O_2Na]^+$ ($[M+Na]^+$): 341.0760, measured: 341.0755. **IR (ATR):** ν (cm^{-1}): 2999, 2936, 2288, 1934, 1715, 1611, 1562, 1465, 1404, 1369, 1319, 1271, 1165, 1121, 1102, 1063, 1013, 857, 840, 767, 711, 695, 641, 616, 597, 514.

Ethyl 4-((3-(trifluoromethyl)phenyl)ethynyl)benzoate (3hb)

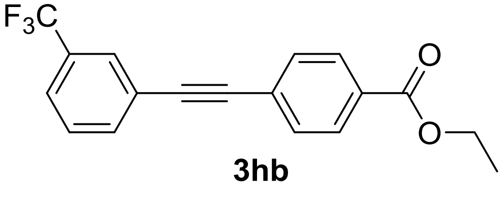

3hb

Prepared from 1-ethynyl-3-(trifluoromethyl)benzene (**1h**) and 4-(ethoxycarbonyl)benzenediazonium tetrafluoroborate (**2b**) on a 0.30 mmol scale. Purified by column chromatography on silica gel (eluent = pentane to pentane:EtOAc, 50:1). Isolated as a mixture with ethyl benzoate side-product, which was subsequently removed by drying under high vacuum (< 1 mbar) overnight. White solid (68 mg, 0.21 mmol, 71%).

^{1}H NMR (400 MHz, $CDCl_3$): δ (ppm): 1.41 (t, $^3J_{H,H}$ = 7.1 Hz, 3H), 4.39 (q, $^3J_{H,H}$ = 7.1 Hz, 2H), 7.49 (tm, $^3J_{H,H}$ = 7.8 Hz, 1H), 7.57 – 7.63 (m, 3H), 7.71 (dm, $^3J_{H,H}$ = 7.8 Hz, 1H), 7.81 (s, 1H), 8.04 (dm, $^3J_{H,H}$ = 8.5 Hz, 2H). **^{13}C NMR (101 MHz, $CDCl_3$):** δ (ppm): 14.5 (CH_3), 61.4 (CH_2), 90.2 (C_q), 90.6 (C_q), 123.8 (q, $^1J_{C,F}$ = 272 Hz, C_q), 123.9 (C_q), 125.4 (q, $^3J_{C,F}$ = 4 Hz, CH), 127.2 (C_q), 128.7 (q, $^3J_{C,F}$ = 4 Hz, CH), 129.1 (CH), 129.7 (CH), 130.5 (C_q), 131.2 (q, $^2J_{C,F}$ = 33 Hz, C_q), 131.7 (CH), 134.9 (q, $^5J_{C,F}$ = 1 Hz, CH), 166.1 (C_q). **^{19}F NMR (282 MHz, $CDCl_3$):** δ (ppm): −63.0 (s). **R_f (pentane:EtOAc 20:1):** 0.59. **GC-MS: t_R (50_40):** 9.4 min. **EI-MS:** *m/z* (%): 319 (12), 318 (61), 290 (31), 274 (21), 273 (100), 245 (13), 225 (19), 176 (14), 136 (5). **HR-MS (ESI):** m/z calculated for $[C_{18}H_{13}F_3O_2Na]^+$ ($[M+Na]^+$): 341.0760, measured: 341.0773. **IR (ATR):** ν (cm^{-1}): 3069, 2990, 2945, 2135, 1711, 1604, 1561, 1508, 1477, 1428, 1404, 1369, 1336, 1271, 1164, 1130, 1102, 1070, 1021, 912, 891, 873, 856, 805, 768, 735, 716, 693, 660, 630, 591, 574, 560, 536.

Ethyl 4-((2-(trifluoromethyl)phenyl)ethynyl)benzoate (3ib)[13]

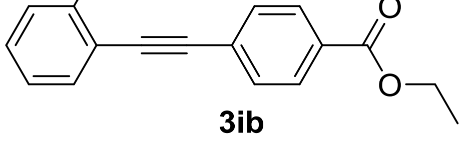

3ib

Prepared from 1-ethynyl-2-(trifluoromethyl)benzene (**1i**) and 4-(ethoxycarbonyl)benzenediazonium tetrafluoroborate (**2b**) on a 0.30 mmol scale. Purified by column chromatography on silica gel (eluent = pentane to pentane:EtOAc, 20:1). White solid (69 mg, 0.22 mmol, 72%).

^{1}H NMR (300 MHz, CDCl$_3$): δ (ppm): 1.41 (t, $^3J_{H,H}$ = 7.1 Hz, 3H), 4.39 (q, $^3J_{H,H}$ = 7.1 Hz, 2H), 7.44 (tm, $^3J_{H,H}$ = 7.6 Hz, 1H), 7.53 (tm, $^3J_{H,H}$ = 7.5 Hz, 1H), 7.60 (dm, $^3J_{H,H}$ = 8.6 Hz, 2H), 7.66−7.72 (m, 2H), 8.04 (dm, $^3J_{H,H}$ = 8.6 Hz, 2H). **^{13}C NMR (75.5 MHz, CDCl$_3$):** δ (ppm): 14.4 (CH$_3$), 61.3 (CH$_2$), 88.1 (C$_q$), 94.1 (C$_q$), 121.1 (q, $^3J_{C,F}$ = 2 Hz, C$_q$), 123.7 (q, $^1J_{C,F}$ = 273 Hz, C$_q$), 126.1 (q, $^3J_{C,F}$ = 5 Hz, CH), 127.3 (C$_q$), 128.6 (CH), 129.6 (CH), 130.5 (C$_q$), 131.6 (CH), 131.7 (CH), 131.8 (q, $^2J_{C,F}$ = 31 Hz, C$_q$), 134.0 (CH), 166.1 (C$_q$). **^{19}F NMR (282 MHz, CDCl$_3$):** δ (ppm): −62.3 (s). **R$_f$ (pentane:EtOAc 20:1):** 0.43. **GC-MS: t$_R$ (50_40):** 9.4 min. **EI-MS:** *m/z* (%): 319 (13), 318 (64), 299 (5), 291 (5), 290 (32), 274 (20), 273 (100), 246 (5), 245 (27), 243 (7), 226 (5), 225 (26), 219 (5), 214 (6), 199 (5), 175 (6), 122 (5). **HR-MS (ESI):** m/z calculated for [$C_{18}H_{13}F_3O_2Na$]$^+$ ([M+Na]$^+$): 341.0760, measured: 341.0765. **IR (ATR):** ν (cm^{-1}): 2984, 2935, 2911, 1716, 1607, 1574, 1513, 1482, 1451, 1405, 1369, 1307, 1291, 1268, 1174, 1122, 1103, 1052, 1028, 961, 922, 858, 840, 765, 719, 691, 652, 614, 594.

Ethyl 4-(oct-1-yn-1-yl)benzoate (3jb)[14]

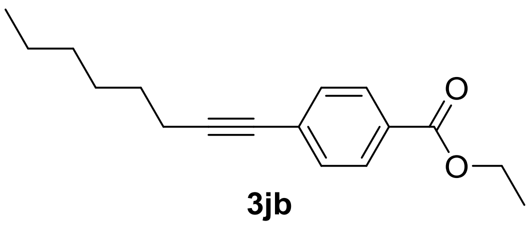

3jb

Prepared from 1-octyne (**1j**) and 4-(ethoxycarbonyl)benzenediazonium tetrafluoroborate (**2b**) on a 0.30 mmol scale. Purified by column chromatography on silica gel (eluent = pentane to pentane:EtOAc, 100:1). Isolated as a mixture with ethyl benzoate side-product, which was subsequently removed by drying under high vacuum (< 1 mbar) overnight. Colorless oil (42 mg, 0.16 mmol, 54%).

^{1}H NMR (300 MHz, CDCl$_3$): δ (ppm): 0.90 (tm, $^3J_{H,H}$ = 6.9 Hz, 3H), 1.23 – 1.52 (m, 9H), 1.55 – 1.68 (m, 2H), 2.42 (t, $^3J_{H,H}$ = 7.0 Hz, 2H), 4.36 (q, $^3J_{H,H}$ = 7.1 Hz, 2H), 7.44 (dm, $^3J_{H,H}$ = 8.5 Hz, 2H), 7.95 (dm, $^3J_{H,H}$ = 8.5 Hz, 2H). **^{13}C NMR (75.5 MHz, CDCl$_3$):** δ (ppm): 14.2 (CH$_3$), 14.4 (CH$_3$), 19.7 (CH$_2$), 22.7 (CH$_2$), 28.7 (CH$_2$), 28.8 (CH$_2$), 31.5 (CH$_2$), 61.2 (CH$_2$), 80.3 (C$_q$), 94.1 (C$_q$), 128.9 (C$_q$), 129.3 (C$_q$), 129.5 (CH), 131.5 (CH), 166.3 (C$_q$). **R$_f$ (pentane:EtOAc 20:1):** 0.76. **GC-MS: t$_R$ (50_40):** 9.2 min. **EI-MS:** *m/z* (%): 259 (6), 258 (30), 229 (16), 216 (21), 215 (31), 214 (8), 213 (43), 189 (5), 187 (26), 185 (9), 173 (5), 169 (7), 163 (11), 161 (6), 160 (7), 159 (27), 157 (12), 156 (9), 155 (17), 146 (9), 145 (11), 144 (9), 143 (57), 142 (28), 141 (22), 131 (21), 130 (15), 129 (100), 128 (51), 127 (19), 126 (5), 119 (5), 118 (6), 117 (52), 116 (11), 115 (48), 114 (34), 113 (14), 102 (7), 101 (8), 95 (9), 91 (15), 89 (7), 88 (11), 79 (5), 77 (13), 75 (6), 67 (6), 63 (10), 55 (7), 51 (5), 43 (21), 41 (22), 39 (9). **HR-MS (ESI):** m/z calculated for [$C_{17}H_{22}O_2Na$]$^+$ ([M+Na]$^+$): 281.1512, measured: 281.1516. **IR (ATR):** ν (cm^{-1}): 2931, 2858, 2227, 1717, 1607, 1505, 1464, 1405, 1367, 1306, 1269, 1174, 1104, 1021, 857, 769, 726, 697, 630, 555, 535, 525.

Ethyl 4-(5-phenylpent-1-yn-1-yl)benzoate (3kb)

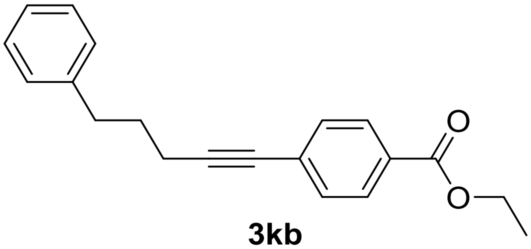

3kb

Prepared from pent-4-yn-1-ylbenzene (**1k**) and 4-(ethoxycarbonyl)benzenediazonium tetrafluoroborate (**2b**) on a 0.30 mmol scale. Purified by column chromatography on silica gel (eluent = pentane to pentane:EtOAc, 50:1). Isolated as a mixture with ethyl benzoate side-product, which was subsequently removed by drying under high vacuum (< 1 mbar) overnight. Colorless oil (54 mg, 0.18 mmol, 62%).

^{1}H NMR (300 MHz, $CDCl_3$): δ (ppm): 1.40 (t, $^3J_{H,H}$ = 7.1 Hz, 3H), 1.89 – 2.01 (m, 2H), 2.46 (t, $^3J_{H,H}$ = 7.0 Hz, 2H), 2.80 (dd, $^3J_{H,H}$ = 7.7, 7.3 Hz, 2H), 4.38 (q, $^3J_{H,H}$ = 7.1 Hz, 2H), 7.17 – 7.26 (m, 3H), 7.27 – 7.35 (m, 2H), 7.47 (dm, $^3J_{H,H}$ = 8.5 Hz, 2H), 7.98 (dm, $^3J_{H,H}$ = 8.5 Hz, 2H). **^{13}C NMR (75.5 MHz, $CDCl_3$):** δ (ppm): 14.5 (CH_3), 19.0 (CH_2), 30.2 (CH_2), 35.0 (CH_2), 61.2 (CH_2), 80.8 (C_q), 93.4 (C_q), 126.1 (CH), 128.5 (CH), 128.7 (CH), 128.8 (C_q), 129.4 (C_q), 129.5 (CH), 131.6 (CH), 141.6 (C_q), 166.3 (C_q). **R_f (pentane:EtOAc 20:1):** 0.63. **GC-MS: t_R (50_40):** 10.5 min. **EI-MS:** *m/z* (%): 293 (7), 292 (47), 291 (7), 263 (11), 247 (35), 220 (11), 219 (68), 218 (7), 214 (13), 204 (19), 203 (14), 191 (28), 178 (7), 155 (7), 143 (21), 142 (13), 141 (12), 129 (67), 128 (32), 127 (16), 117 (6), 115 (27), 114 (13), 105 (10), 105 (8), 104 (8), 104 (27), 103 (21), 92 (24), 91 (100), 89 (7), 88 (5), 79 (13), 78 (18), 77 (29), 65 (12), 63 (9), 51 (12), 43 (5). **HR-MS (ESI):** m/z calculated for $[C_{20}H_{20}O_2Na]^+$ ($[M+Na]^+$): 315.1356, measured: 315.1345. **IR (ATR):** ν (cm^{-1}): 3028, 2929, 2833, 2247, 1714, 1650, 1606, 1496, 1456, 1428, 1344, 1268, 1173, 1102, 1026, 989, 912, 854, 746, 698, 631, 586, 567, 550, 515.

Ethyl 4-(5-cyanopent-1-yn-1-yl)benzoate (3lb)

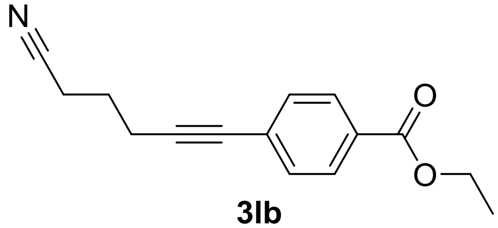

3lb

Prepared from hex-5-ynenitrile (**1l**) and 4-(ethoxycarbonyl)benzenediazonium tetrafluoroborate (**2b**) on a 0.30 mmol scale. Purified by column chromatography on silica gel (eluent = pentane:EtOAc, 10:1 to 5:1). Orange oil (50 mg, 0.21 mmol, 69%).

^{1}H NMR (300 MHz, $CDCl_3$): δ (ppm): 1.38 (t, $^3J_{H,H}$ = 7.1 Hz, 3H), 1.97 (pent, $^3J_{H,H}$ = 7.0 Hz, 2H), 2.56 (t, $^3J_{H,H}$ = 7.1 Hz, 2H), 2.62 (t, $^3J_{H,H}$ = 6.8 Hz, 2H), 4.36 (q, $^3J_{H,H}$ = 7.2 Hz, 2H), 7.44 (dm, $^3J_{H,H}$ = 8.5 Hz, 2H),

7.96 (dm, $^3J_{H,H}$ = 8.5 Hz, 2H). **^{13}C NMR (75.5 MHz, CDCl$_3$):** δ (ppm): 14.4 (CH$_3$), 16.4 (CH$_2$), 18.7 (CH$_2$), 24.6 (CH$_2$), 61.2 (CH$_2$), 81.9 (C$_q$), 90.2 (C$_q$), 119.2 (C$_q$), 127.9 (C$_q$), 129.5 (CH), 129.8 (C$_q$), 131.6 (CH), 166.1 (C$_q$). **R$_f$ (pentane:EtOAc 5:1):** 0.24. **GC-MS: t$_R$ (50_40):** 9.5 min. **EI-MS:** *m/z* (%): 242 (6), 241 (34), 197 (15), 196 (100), 187 (5), 168 (21), 167 (23), 159 (6), 155 (6), 141 (5), 128 (10), 127 (6), 115 (10), 114 (8), 113 (5), 77 (5), 63 (5). **HR-MS (ESI):** m/z calculated for $[C_{15}H_{15}NO_2Na]^+$ ($[M+Na]^+$): 264.0995, measured: 264.1001. **IR (ATR):** ν (cm^{-1}): 2982, 2934, 2910, 2250, 2230, 1946, 1704, 1606, 1563, 1509, 1477, 1449, 1429, 1408, 1366, 1341, 1313, 1275, 1178, 1126, 1104, 1023, 982, 944, 857, 769, 719, 697, 642, 603, 580, 542, 527.

Ethyl 4-(cyclopentylethynyl)benzoate (3mb)

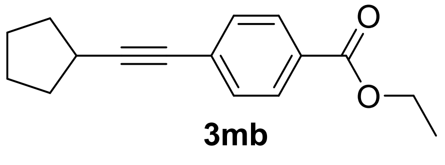

3mb

Prepared from cyclopentylacetylene (**1m**) and 4-(ethoxycarbonyl)benzenediazonium tetrafluoroborate (**2b**) on a 0.30 mmol scale. Purified by column chromatography on silica gel (eluent = pentane to pentane:EtOAc, 100:1). Isolated as a mixture with ethyl benzoate side-product, which was subsequently removed by drying under high vacuum (< 1 mbar) overnight. Colorless oil (46 mg, 0.19 mmol, 63%).

^{1}H NMR (300 MHz, CDCl$_3$): δ (ppm): 1.38 (t, $^3J_{H,H}$ = 7.1 Hz, 3H), 1.54 – 1.84 (m, 6H), 1.94 – 2.07 (m, 2H), 2.84 (pent, $^3J_{H,H}$ = 7.5 Hz, 1H), 4.36 (q, $^3J_{H,H}$ = 7.1 Hz, 2H), 7.43 (dm, $^3J_{H,H}$ = 8.5 Hz, 2H), 7.95 (dm, $^3J_{H,H}$ = 8.5 Hz, 2H). **^{13}C NMR (75.5 MHz, CDCl$_3$):** δ (ppm): 14.5 (CH$_3$), 25.2 (CH$_2$), 31.0 (CH), 33.9 (CH$_2$), 61.2 (CH$_2$), 79.8 (C$_q$), 98.2 (Cq), 129.0 (C$_q$), 129.1 (C$_q$), 129.4 (CH), 131.5 (CH), 166.4 (C$_q$). **R$_f$ (pentane:EtOAc 20:1):** 0.67. **GC-MS: t$_R$ (50_40):** 9.1 min. **EI-MS:** *m/z* (%): 243 (10), 242 (57), 214(14), 213 (16), 198 (6), 197 (37), 186 (9), 185 (10), 172 (7), 170 (14), 169 (95), 168 (10), 167 (14), 165 (7), 156 (6), 155 (38), 154 (16), 153 (17), 152 (10), 146 (5), 142 (19), 141 (100), 140 (9), 139 (18), 129 (22), 128 (21), 127 (23), 126 (11), 115 (33), 114 (6), 113 (6), 102 (5), 101 (10), 91 (11), 89 (5), 77 (13), 76 (5), 75 (8), 67 (5), 63 (9), 51 (6), 42 (5), 41 (9), 39 (7). **HR-MS (ESI):** m/z calculated for $[C_{16}H_{18}O_2Na]^+$ ($[M+Na]^+$): 265.1199, measured: 265.1200. **IR (ATR):** ν (cm^{-1}): 2958, 2873, 2225, 1716, 1700, 1606, 1561, 1507, 1469, 1447, 1405, 1368, 1328, 1307, 1269, 1175, 1105, 1019, 972, 900, 859, 769, 733, 697, 629, 560, 536, 522.

4-(phenylethynyl)benzonitrile (3fa)[15]

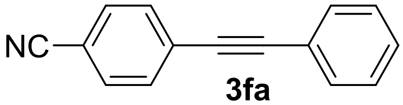

3fa

Prepared from 4-ethynylbenzonitrile (**1f**) and benzenediazonium tetrafluoroborate (**2a**) on a 0.30 mmol scale. Purified by column chromatography on silica gel (eluent = pentane:EtOAc, 20:1 to 10:1) and drying under high vacuum (< 1 mbar) overnight. Yellow solid (46 mg, 0.226 mmol, 75 %).

^{1}H NMR (300 MHz, CDCl$_3$): δ (ppm): 7.38-7.39 (m, 3H), 7.54-7.65 (m, 6H). **^{13}C NMR (75.5 MHz, CDCl$_3$):** δ (ppm): 88.1 (C_q), 94.1 (C_q), 111.8 (C_q), 118.9 (C_q), 122.5 (C_q), 128.6 (C_q), 128.8 (CH), 129.5 (CH), 132.1 (CH), 132.4 (CH), 132.4 (CH). **R_f (pentane:EtOAc 10:1):** 0.47. **GC-MS: t_R (50_40):** 9.0 min. **EI-MS:** *m/z* (%): 204 (17), 203 (100), 202 (10), 201 (9), 177 (6), 176 (7), 175 (7). **HR-MS (ESI):** *m/z* calculated for $[C_{15}H_9NNa]^+$ ($[M+Na]^+$): 226.0633, measured: 226.0632. **IR (ATR):** ν (cm^{-1}): 691, 759, 840, 1072, 1178, 1274, 1407, 1444, 1503, 1603, 2216, 2225, 3089.

4-(p-tolylethynyl)benzonitrile (3fc)[16]

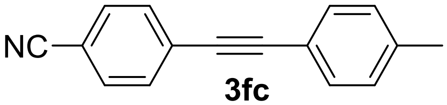

3fc

Prepared from 4-ethynylbenzonitrile (**1f**) and 4-methylbenzenediazonium tetrafluoroborate (**2c**) on a 0.30 mmol scale. Purified by column chromatography on silica gel (eluent = pentane:EtOAc, 20:1 to 10:1). The resulting solid was washed three times with 3 ml of pentane and dried under high vacuum (< 1 mbar) for 2h. Yellow solid (40 mg, 0.184 mmol, 61 %).

^{1}H NMR (300 MHz, CDCl$_3$): δ (ppm): 2.30 (s, 3H), 7.18-7.20 (m, 2H), 7.43-7.45 (m, 2H), 7.57-7.64 (m, 4H). **^{13}C NMR (75.5 MHz, CDCl$_3$):** δ (ppm): 21.9 (CH_3), 87.5 (C_q), 94.4 (C_q), 111.5 (C_q), 118.9 (C_q), 119.4 (C_q), 128.8 (C_q), 129.6 (CH), 132.0 (CH), 132.3 (CH), 132.4 (CH), 139.7 (C_q). **R_f (pentane:EtOAc 10:1):** 0.49. **GC-MS: t_R (50_40):** 9.3 min. **EI-MS:** *m/z* (%): 218 (18), 217 (100), 216 (42), 215 (6), 214 (12), 190 (14), 189 (10), 94 (6). **HR-MS (ESI):** *m/z* calculated for $[C_{16}H_{11}NNa]^+$ ($[M+Na]^+$): 240.0789, measured: 240.0790; **IR (ATR)**: ν (cm^{-1}): 756, 819, 839,1018, 1044, 1108, 1132, 1177, 1230, 1377, 1497, 1509, 1596, 1742, 2211, 2226, 2924, 2959.

4,4'-(ethyne-1,2-diyl)dibenzonitrile (3fd)[17]

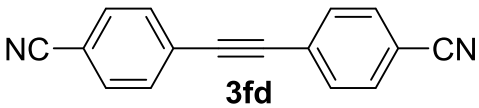

3fd

Prepared from 4-ethynylbenzonitrile (**1f**) and 4-cyanobenzenediazonium tetrafluoroborate (**2d**) on a 0.30 mmol scale. Purified by column chromatography on silica gel (eluent = pentane:EtOAc, 5:1 to 2:1). The resulting solid was washed three times with 3 ml of pentane and dried under high vacuum (< 1 mbar) for 2h. Yellow solid (50 mg, 0.219 mmol, 73 %).

Prepared on 0.30 mmol scale using 2.5 mol % of $[Ru(bpy)_3](PF_6)_2$ under air. (45 mg, 0.197 mmol, 66 %).

^{1}H NMR (300 MHz, $CDCl_3$): δ (ppm): 7.62-7.68 (q, $^3J_{H,H}$ = 8.4 Hz, 8H). **^{13}C NMR (75.5 MHz, $CDCl_3$):** δ (ppm): 91.8 (C_q), 112.7 (C_q), 118.5 (C_q), 127.3 (C_q), 132.5 (CH), 132.6 (CH). **R_f (pentane:EtOAc 2:1):** 0.58. **GC-MS: t_R (50_40):** 10.0 min. **EI-MS:** *m/z* (%): 229 (18), 228 (100), 227 (7), 201 (7), 200 (6). **HR-MS (ESI):** *m/z* calculated for $[C_{16}H_8N_2Na]^+$ ($[M+Na]^+$): 251.0585, measured: 251.0570; **IR (ATR):** ν (cm^{-1}): 830, 1099, 1172, 1277, 1405, 1504, 1604, 1920, 2226.

4-((3-(trifluoromethyl)phenyl)ethynyl)benzonitrile (3fe)[18]

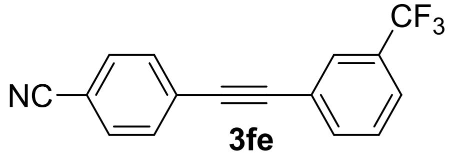

3fe

Prepared from 4-ethynylbenzonitrile (**1f**) and 3-(trifluoromethyl)benzenediazonium tetrafluoroborate (**2e**) on a 0.30 mmol scale. Purified by column chromatography on silica gel (eluent = pentane:EtOAc, 20:1 to 10:1). The resulting solid was washed three times with 3 ml of pentane and dried under high vacuum (< 1 mbar) for 2h. White solid (48 mg, 0.180 mmol, 59 %).

^{1}H NMR (600 MHz, $CDCl_3$): δ (ppm): 7.51 (t, $^3J_{H,H}$ = 7.8 Hz, 1H), 7.61 – 7.67 (m, 5H), 7.81 (s, 1H), 7.71 (d, $^3J_{H,H}$ = 7.7 Hz, 1H). **^{13}C NMR (150 MHz, $CDCl_3$):** δ (ppm): 89.2 (C_q), 92.0 (C_q), 112.2 (C_q), 118.5 (C_q), 123.4 (C_q), 123.7 (q, $^1J_{C,F}$ = 272.5 Hz, CF_3), 125.8 (*q*, $^3J_{C,F}$ = 3.7 Hz, CH), 127.6 (C_q), 128.74 (*q*, $^3J_{C,F}$ = 3.9 Hz, CH), 129.2 (CH), 131.4 (*q*, $^2J_{C,F}$ = 32.7 Hz, C_q), 132.3 (CH), 132.3 (CH), 135.0 (*q*, $^4J_{C,F}$ = 1.1 Hz, CH). **^{19}F NMR (600 MHz, $CDCl_3$):** δ (ppm): –63.03 (s). **R_f (pentane:ethylacetate 9:1):** 0.38. **GC-MS: t_R (50_40):** 8.9 min. **EI-MS:** *m/z* (%): 272 (18), 271 (100). **HR-MS (ESI):** *m/z* calculated for $[C_{16}H_8F_3NNa]^+$ ($[M+Na]^+$): 294.0501, measured: 294.0511; **IR (ATR):** ν (cm^{-1}): 2225, 1604, 1503, 1482, 1430, 1409, 1338, 1296, 1270, 1171, 1155, 1114, 1093, 1070, 1021, 891, 837, 804, 743, 694, 666, 653, 603.

4-((4-fluorophenyl)ethynyl)benzonitrile (3ff)[19]

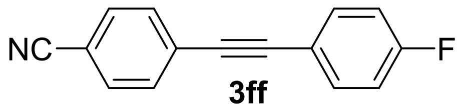

3ff

Prepared from 4-ethynylbenzonitrile (**1f**) and 3-(trifluoromethyl)benzenediazonium tetrafluoroborate (**2f**) on a 0.30 mmol scale. Purified by column chromatography on silica gel (eluent = pentane:EtOAc, 20:1 to 10:1). The resulting solid was washed three times with 3 ml of pentane and dried under high vacuum (< 1 mbar) for 2h. White solid (37 mg, 0.17 mmol, 56%).

^{1}H NMR (300 MHz, $CDCl_3$): δ (ppm): 7.03 – 7.12 (m, 2H), 7.48–7.56 (m, 2H), 7.57 – 7.67 (m, 4H). **^{13}C NMR (75.5 MHz, $CDCl_3$):** δ (ppm): 87.6 (C_q), 92.8 (C_q), 111.8 (C_q), 115.9 (CH), 116.2 (CH), 118.5 (d, $^4J_{C,F}$ = 3.5 Hz, C_q), 118.6 (C_q), 128.2 (C_q), 132.2 (d, $^3J_{C,F}$ = 3.6 Hz, CH), 133.9 (d, $^3J_{C,F}$ = 8.5 Hz, CH), 163.1 (d, $^1J_{C,F}$ = 251.1 Hz, C_q). **^{19}F NMR (282 MHz, $CDCl_3$):** δ (ppm): – 109.30 (tt, $^3J_{F,H}$ = 8.5 Hz, $^4J_{F,H}$ = 5.3 Hz). **R_f (pentane:ethylacetate 9:1):** 0.32. **GC-MS: t_R (50_40):** 9.0 min. **EI-MS:** *m/z* (%): 222 (16), 221 (100). **HR-MS (ESI):** *m/z* calculated for $[C_{15}H_8FNNa]^+$ ($[M+Na]^+$): 244.0533, measured: 244.0540. **IR (ATR):** ν (cm^{-1}): 2231, 2212, 1594, 1508, 1411, 1275, 1227, 1184, 1158, 1132, 1111, 1099, 1013, 912, 837, 811, 732, 654.

4-((4-bromophenyl)ethynyl)benzonitrile (3fg)[18]

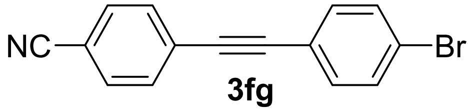

3fg

Prepared from 4-ethynylbenzonitrile (**1f**) and 4-bromobenzenediazonium tetrafluoroborate (**2g**) on a 0.30 mmol scale. Purified by column chromatography on silica gel (eluent = pentane:EtOAc, 20:1 to 10:1). The resulting solid was washed three times with 3 ml of pentane and dried under high vacuum (< 1 mbar) for 2h. Yellow solid (73 mg, 0.258 mmol, 86 %).

Prepared on 0.30 mmol scale using 2.5 mol % of $[Ru(bpy)_3](PF_6)_2$ under air. (57 mg, 0.201 mmol, 67 %).

^{1}H NMR (300 MHz, $CDCl_3$): δ (ppm): 7.38 – 7.41 (m, 2H), 7.50 – 7.53 (m, 2H), 7.58 – 7.66 (m, 4H). **^{13}C NMR (75.5 MHz, $CDCl_3$):** δ (ppm): 89.0 (C_q), 92.9 (C_q), 112.0 (C_q), 118.7 (C_q), 121.4 (C_q), 123.8 (C_q), 128.1 (C_q), 132.15 (CH), 132.38 (CH), 132.42 (CH), 133.4 (CH). **R_f (pentane:EtOAc 10:1):** 0.58. **GC-MS: t_R (50_40):** 9.9 min. **EI-MS:** *m/z* (%): 283.9 (17), 289.0 (99), 282 (18), 280 (100), 202 (6), 201 (34), 176 (13), 175 (22), 174 (8), 151 (6). **HR-MS (ESI):** *m/z* calculated for $[C_{15}H_8BrNNa]^+$ ($[M+Na]^+$): 303.9738, measured: 303.9728. **IR (ATR)**: ν (cm^{-1}): 602, 659, 824, 1010, 1065, 1102, 1176, 1274, 1307, 1394, 1475, 1502, 1582, 1600, 2217.

4-((4-chlorophenyl)ethynyl)benzonitrile (3fh)[20]

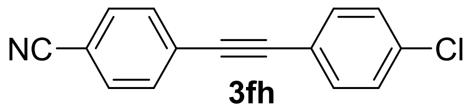

3fh

Prepared from 4-ethynylbenzonitrile (**1f**) and 4-chlorobenzenediazonium tetrafluoroborate (**2h**) on a 0.30 mmol scale. Purified by column chromatography on silica gel (eluent = pentane:EtOAc, 20:1 to 10:1). The resulting solid was washed three times with 3 ml of pentane and dried under high vacuum (< 1 mbar) for 2h. Yellow solid (46 mg, 0.193 mmol, 64 %).

^{1}H NMR (300 MHz, $CDCl_3$): δ (ppm): 7.34 – 7.36 (m, 2H), 7.46 – 7.48 (m, 2H), 7.58 – 7.65 (m, 4H). **^{13}C NMR (75.5 MHz, $CDCl_3$):** δ (ppm): 88.9 (C_q), 92.8 (C_q), 112.0 (C_q), 118.7 (C_q), 121.0 (C_q), 128.2 (C_q), 129.2 (CH), 132.3 (CH), 132.4 (CH), 133.3 (CH), 135.6 (C_q). **R_f (pentane:EtOAc 10:1):** 0.43. **GC-MS: t_R (50_40):** 9.6 min. **EI-MS:** *m/z* (%): 239 (34), 238 (17), 237 (100), 201 (18), 175 (6), 175 (11). **HR-MS (ESI):** *m/z* calculated for $[C_{15}H_8ClNNa]^+$ ($[M+Na]^+$): 260.0243, measured: 260.0230. **IR (ATR)**: ν (cm^{-1}): 558, 674, 828, 1011, 1090, 1175, 1273, 1397, 1477, 1497, 1589, 1600, 1920, 2216, 2340, 2359.

4-((4-acetylphenyl)ethynyl)benzonitrile (3fi)[21]

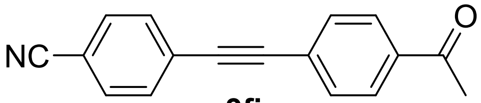

3fi

Prepared from 4-ethynylbenzonitrile (**1f**) and 4-acetylbenzenediazonium tetrafluoroborate (**2i**) on a 0.30 mmol scale. Purified by column chromatography on silica gel (eluent = pentane:EtOAc, 5:1 to 2:1). White solid (55 mg, 0.224 mmol, 74 %).

Prepared on 0.30 mmol scale using 2.5 mol % of $[Ru(bpy)_3](PF_6)_2$ under air. (48 mg, 0.195 mmol, 65 %).

^{1}H NMR (300 MHz, $CDCl_3$): δ (ppm): 2.62 (s, 3H), 7.61-7.68 (m, 6H), 7.95-7.98 (m, 2H). **^{13}C NMR (75.5 MHz, $CDCl_3$):** δ (ppm): 27.0 (CH_3), 90.9 (C_q), 92.6 (C_q), 112.3 (C_q), 118.6 (C_q), 127.2 (C_q), 127.8 (C_q), 128.6 (CH), 132.2 (CH), 132.4 (CH), 132.5 (CH), 137.1 (C_q), 197.5 (C_q). **R_f (pentane:EtOAc 2:1):** 0.48. **GC-MS: t_R (50_40):** 10.3 min. **EI-MS:** *m/z* (%): 246 (9), 245 (49), 231 (19), 230 (100), 202 (13), 201 (28), 176 (10), 175 (18), 115 (6), 101 (7), 43 (7). **HR-MS (ESI):** *m/z* calculated for $[C_{17}H_{11}NNaO]^+$ ($[M+Na]^+$): 268.0738, measured: 268.0736; **IR (ATR):** ν (cm^{-1}): 592, 696, 827, 839, 864, 961, 1014, 1111, 1174, 1262, 1356, 1404, 1493, 1555, 1602, 1683, 1934, 2225.

methyl 2-((4-cyanophenyl)ethynyl)benzoate (3fj)[14]

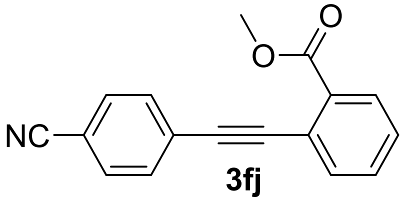

3fj

Prepared from 4-ethynylbenzonitrile (**1f**) 2-(methoxycarbonyl)benzenediazonium tetrafluoroborate (**2j**) on a 0.30 mmol scale. Purified by column chromatography on silica gel (eluent = pentane:EtOAc, 5:1 to 2:1). White solid (32 mg, 0.122 mmol, 40 %).

^{1}H NMR (400 MHz, CDCl$_3$): δ (ppm): 3.96 (s, 3H), 7.44 (td, $^3J_{H,H}$ = 7.6 Hz, $^4J_{H,H}$ = 1.4 Hz, 1H), 7.53 (td, $^3J_{H,H}$ = 7.6 Hz, $^4J_{H,H}$ = 1.4 Hz, 1H), 7.60-7.67 (m, 5H), 8.02 (dd, $^3J_{H,H}$ = 7.9 Hz, $^4J_{H,H}$ = 1.4 Hz, 1H). **^{13}C NMR (75.5 MHz, CDCl$_3$):** δ (ppm): 52.6 (CH_3), 92.7 (C_q), 92.8 (C_q), 118.8 (C_q), 120.0 (C_q), 123.1 (C_q), 128.6 (C_q), 129.0 (CH), 130.9 (CH), 132.2 (CH), 132.3 (CH), 132.5 (CH), 133.3 (C_q), 134.4 (CH), 166.5 (C_q). **R_f (pentane:EtOAc 2:1):** 0.53. **GC-MS: t_R (50_40):** 10.1 min. **EI-MS:** *m/z* (%): 262 (18), 261 (100), 247 (10), 246 (64), 230 (25), 218 (17), 202 (13), 201 (33), 191 (9), 190 (40), 176 (13), 175 (21), 115 (6), 101 (8), 87 (7), 74 (6). **HR-MS (ESI):** *m/z* calculated for $[C_{17}H_{11}NO_2Na]^+$ ($[M+Na]^+$): 284.0887, measured: 284.0682. **IR (ATR)**: ν (cm^{-1}): 668, 697, 756, 765, 835, 966, 1044, 1087, 1125, 1250, 1293, 1448, 1457, 1605, 1694, 1710, 2240, 2360.

6-((4-methoxyphenyl)ethynyl)-2,3-dihydrobenzo[b][1,4]dioxine (3ck)

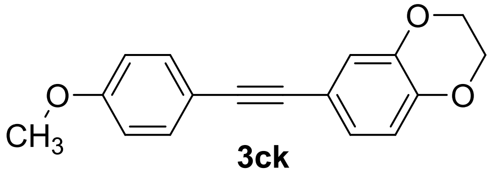

3ck

Prepared from 4-ethynylanisol (**1c**) and 2,3-dihydrobenzo[b][1,4]dioxine-6-diazonium tetrafluoroborate (**2k**) on a 0.30 mmol scale using 2.5 mol% of $[Ru(bpy)_3](PF_6)_2$ and 5W Blue Leds for 5h. Purified by column chromatography on silica gel (eluent = pentane:EtOAc, 10:1 to 5:1). White solid (39 mg, 0.146 mmol, 49 %).

^{1}H NMR (400 MHz, CDCl$_3$): δ (ppm): 3.82 (s, 3H), 4.26 (s, 4H), 6.80-6.88 (m, 3H), 6.99-7.03 (m, 2H) 7.42-7.47 (m, 2H). **^{13}C NMR (75.5 MHz, CDCl$_3$):** δ (ppm): 55.6 (CH_3), 64.5 (CH_2), 64.8 (CH_2), 88.0 (C_q), 114.2 (CH), 115.8 (C_q), 116.7 (C_q), 117.6 (C_q), 120.5 (CH), 125.4 (CH), 133.2 (CH), 143.5 (C_q), 144.2 (C_q), 159.7 (C_q). **R_f (pentane:EtOAc 5:1):** 0.58. **GC-MS: t_R (50_40):** 10.8 min. **EI-MS:** *m/z* (%): 267 (10), 266 (100), 251 (23), 210 (34), 182 (10), 167 (7), 156 (8), 139 (15), 125 (7), 112 (7). **HR-MS (ESI):** *m/z* calculated for $[C_{17}H_{14}O_3Na]^+$ ($[M+Na]^+$): 289.0841, measured: 289.0835. **IR (ATR)**: ν (cm^{-1}): 525, 560, 651, 729, 906, 1068, 1247, 1281, 1323, 1513, 1577, 1607, 2254.

1-ethoxy-4-((4-methoxyphenyl)ethynyl)benzene (3cl)[22]

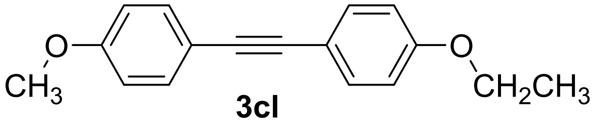

3cl

Prepared from 4-ethynylanisol (**1c**) and 4-ethoxybenzenediazonium tetrafluoroborate (**2l**) on a 0.30 mmol scale using 2.5 mol% of $[Ru(bpy)_3](PF_6)_2$ and 5W Blue Leds for 5h. Purified by column chromatography on silica gel (eluent = pentane:EtOAc, 10:1 to 5:1). White solid (38 mg, 0.150 mmol, 50 %).

^{1}H NMR (400 MHz, $CDCl_3$): δ (ppm): 1.42 (t, $^3J_{H,H}$ = 7.0 Hz, 3H), 3.82 (s, 3H), 4.0 (q, $^3J_{H,H}$ = 7.0 Hz, 2H), 6.83-6.89 (m, 4H), 7.42-7.47 (m, 4H). **^{13}C NMR (75.5 MHz, $CDCl_3$):** δ (ppm): 15.0 (CH_3), 55.5 (CH_3), 67.7 (CH_2), 88.0 (C_q), 88.2 (C_q), 114.1 (CH), 114.6 (CH), 115.7 (C_q), 115.9 (C_q), 133.0 (CH), 159.0 (C_q), 159.5 (C_q). **R_f (pentane:EtOAc 5:1):** 0.66. **GC-MS: t_R (50_40):** 9.90 min. **EI-MS:** *m/z* (%): 253 (17), 252 (100), 225 (6), 224 (35), 223 (49), 210 (7), 209 (41), 195 (10), 181 (11), 180 (7), 163 (9), 152 (25), 151 (9), 126 (6). **IR (ATR)**: ν (cm^{-1}): 534, 574, 630, 730, 813, 836, 907, 1029, 1047, 1114, 1176, 1245, 1286, 1307, 1399, 1443, 1475, 1517, 1570, 1607, 2985.

4. Further Manipulations of Brominated Diarylalkynes

Ethyl 4-((4-(phenylethynyl)phenyl)ethynyl)benzoate (4)[23]

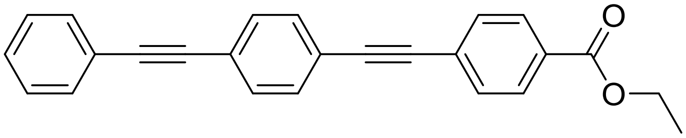

4

Following a modified procedure from Fischmeister et al,[22] in a dry Schlenk tube, ethyl 4-((4-bromophenyl)ethynyl)benzoate (**3eb**, 50 mg, 0.15 mmol, 1.0 equiv.), phenylacetylene (**1b**, 19 µL, 0.17 mmol, 1.1 equiv), $Pd(PPh_3)_2Cl_2$ (2.2 mg, 3.0 µmol, 2.0 mol%) and PPh_3 (1.6 mg, 6.1 µmol, 4.0 mol%) were dissolved in dry triethylamine (0.70 mL). The mixture was stirred under argon for 5 min at rt before CuI (1.2 mg, 6.1 µmol, 4.0 mol%) was added. The tube was re-sealed under argon and stirred at 60 °C overnight. After cooling to room temperature, the reaction was filtered and the solids were washed with Et_2O (2 mL). The filtrate was then washed with NH_4Cl (sat. aq., 3 mL), HCl (1 M aq., 3 mL), NaOH (1 M aq., 3 mL) and brine (3 mL). After drying over anhydrous Na_2SO_4, the organic fraction was filtered and concentrated *in vacuo*. Column chromatography on silica gel (eluent = pentane:EtOAc, 20:1) afforded **4** as a white solid (26 mg, 74 µmol, 49%).

^{1}H NMR (400 MHz, $CDCl_3$): δ (ppm): 1.41 (t, $^3J_{H,H}$ = 7.1 Hz, 3H), 4.39 (q, $^3J_{H,H}$ = 7.1 Hz, 2H), 7.33 – 7.40 (m, 3H), 7.49 – 7.57 (m, 6H), 7.59 (dm, $^3J_{H,H}$ = 8.5 Hz, 2H), 8.04 (dm, $^3J_{H,H}$ = 8.5 Hz, 2H); **^{13}C NMR (101**

MHz, $CDCl_3$): δ (ppm): 14.5 (CH_3), 61.3 (CH_2), 89.1 (C_q), 90.6 (C_q), 91.7 (C_q), 92.1 (C_q), 112.6 (C_q), 123.1 (C_q), 123.8 (C_q), 127.8 (C_q), 128.6 (CH), 128.7 (CH), 129.7 (CH), 130.2 (C_q), 131.6 (CH), 131.7 (CH), 131.8 (CH), 131.8 (CH), 166.2 (C_q); **R_f (pentane:EtOAc 20:1):** 0.49; **GC-MS: t_R (50_40):** 17.6 min; **EI-MS:** *m/z* (%): 351 (28), 350 (100), 323 (7), 322 (29), 306 (6), 305 (22), 277 (13), 276 (35), 275 (6), 274 (13), 152 (6), 138 (13); **HR-MS (ESI):** m/z calculated for $[C_{25}H_{18}O_2Na]^+$ ($[M+Na]^+$): 373.1199, measured: 373.1198; **IR (ATR):** ν (cm^{-1}): 2933, 2211, 1708, 1601, 1559, 1515, 1499, 1444, 1407, 1366, 1308, 1268, 1173, 1101, 1018, 860, 837, 767, 756, 692, 654, 600, 588, 576, 552.

4-([1,1'-Biphenyl]-4-ylethynyl)benzonitrile (5)[24]

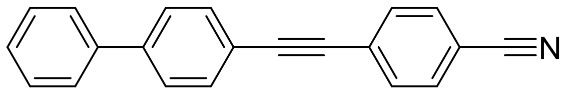

5

Following a modified procedure from Pascal Jr. et al,[21] in a dry Schlenk tube, 4-((4-bromophenyl)ethynyl) benzonitrile (**3fg**, 150 mg, 0.532 mmol, 1.00 equiv.) and phenylboronic acid (81.0 mg, 0.665 mmol, 1.25 equiv) were dissolved in toluene (10.7 mL). An aqueous solution of K_2CO_3 (2 M, 3.60 mL) and ethanol (1.40 mL) were added followed by $Pd(PPh_3)_4$ (31 mg, 27 µmol, 5.0 mol%). The tube was sealed under argon and stirred at 50 °C for 20 h. After cooling to room temperature, the reaction was quenched with NH_4Cl (sat. aq., 10 mL) and extracted with ethyl acetate (2 × 20 mL). The combined organic fractions were washed with brine (30 mL), dried over anhydrous Na_2SO_4, filtered and concentrated *in vacuo*. Column chromatography on silica gel (eluent = pentane:EtOAc, 20:1) afforded **5** as a white solid (146 mg, 0.523 mmol, 98%).

^{1}H NMR (300 MHz, CD_2Cl_2): δ (ppm): 7.39 (m, 1H), 7.44 – 7.52 (m, 2H), 7.59 – 7.70 (m, 10H); **^{13}C NMR (75.5 MHz, CD_2Cl_2):** δ (ppm): 88.9 (C_q), 94.0 (C_q), 112.0 (C_q), 119.0 (C_q), 121.6 (C_q), 127.5 (CH), 127.6 (CH), 128.4 (CH), 128.6 (C_q), 129.5 (CH), 132.5 (CH), 132.7 (CH), 132.8 (CH), 140.5 (C_q), 142.3 (C_q); **R_f (pentane:EtOAc 20:1):** 0.41; **GC-MS: t_R (50_40):** 12.4 min; **EI-MS:** *m/z* (%): 280 (24), 279 (100), 278 (7), 277 (21), 140 (10); **HR-MS (APCI):** m/z calculated for $[C_{21}H_{13}N]^+$ ($[M]^+$): 279.1043, measured: 279.1041; **IR (ATR):** ν (cm^{-1}): 2931, 2168, 1928, 1797, 1681, 1597, 1550, 1525, 1500, 1482, 1448, 1405, 1308, 1275, 1212, 1178, 1137, 1018, 1004, 974, 916, 839, 763, 716, 690, 653, 621, 593, 555.

5. Kinetic Studies

A reaction between phenylacetylene (0.10 mmol), benzenediazonium tetrafluoroborate (**2a**, 0.40 mmol), $[Ru(bpy)_3](PF_6)_2$ (0.5 mol%) and $((p\text{-MeO})C_6H_4)_3PAuCl$ (10 mol%) in degassed DMF (1.0 mL) under 23 W CFL irradiation was performed to determine the kinetic profile of the reaction. The yields of diphenylacetylene (**S1**) after different time intervals were determined by GC-FID using mesitylene as internal standard. A fast reaction (completed within 40 min) was observed without any induction periods (see Figure S3 below).

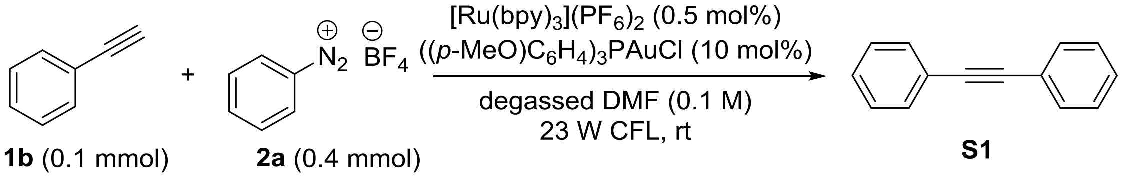

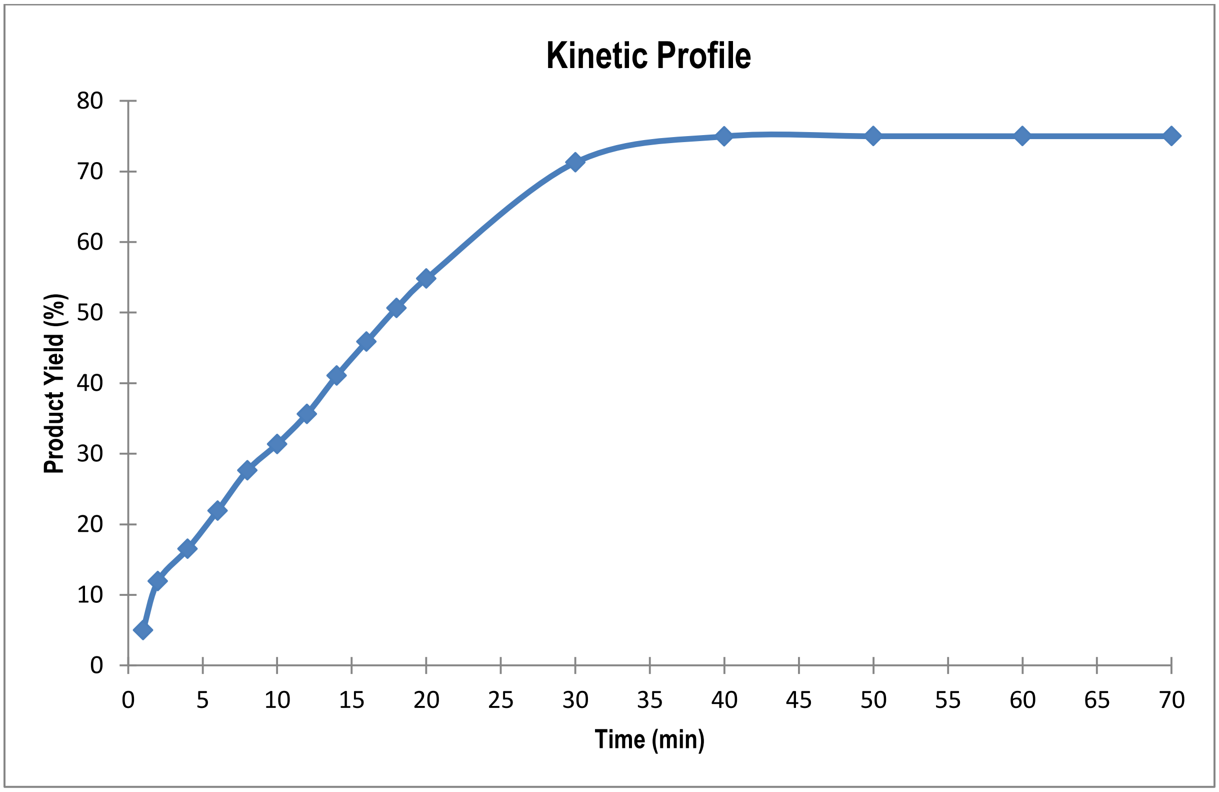

Figure S3. Kinetic profile of the reaction: Average yields of diphenylacetylene (**S1**) after 3 runs are plotted.

6. Quantum Yield Measurement

Following a modified procedure reported by Melchiorre and co-workers,[25] an aq. ferrioxalate actinometer solution was prepared and stored in the dark. The actinometer solution measures the photodecomposition of ferric oxalate anions to ferrous oxalate anions, which are then reacted with 1,10-phenanthroline to form $Fe(Phen)_3^{2+}$. Its concentration is then estimated by UV/Vis absorbance at 510 nm. The number of moles of $Fe(Phen)_3^{2+}$ complex formed is related to the numbers of photons absorbed by the actinometer solution.

Preparation of the solutions used for the studies:

1. Potassium ferrioxalate solution: Potassium ferrioxalate trihydrate (295 mg) and 95-98% H_2SO_4 (140 µL) were added to a 50 mL volumetric flask and filled to the mark with distilled water.
2. Buffer solution: Sodium acetate (4.94 g) and 95-98% H_2SO_4 (1.0 mL) were added to a 100 mL volumetric flask and filled to the mark with distilled water.
3. The reaction solution: $[Ru(bpy)_3](PF_6)_2$ (0.4 mg, 0.46 µmol, 0.5 mol%), $((p\text{-MeO})C_6H_4)_3PAuCl$ (5.8 mg, 9.92 µmol, 10 mol%), the aryldiazonium salt **2a** (0.4 mmol, 4.0 equiv) and phenylacetylene (0.1 mmol, 1.0 equiv) were added to a quartz cuvette (l = 1 cm) containing a stirring bar. In the absence of light, anhydrous DMF (1.0 mL, 0.10 M) was added under argon.

The actinometry measurements:

a) 1 mL of the actinometer solution was taken in a quartz cuvette (l = 1 cm). Both the cuvettes of actinometer solution and reaction solution were placed next to each other at a distance of 5 cm away from a 5 W blue LED (λ_{max} = 465 nm) featured with a bandpass filter (450 ± 2 nm) (Thorlabs FB450-10) and irradiated for 150 s. The same process was repeated for different time intervals: 300, 450 and 600 s.
b) After irradiation, the actinometer solution was transferred to a 10 mL volumetric flask containing 1.0 mg of 1,10-phenanthroline in 2 ml of buffer solution. The flask was filled to the mark with distilled water. In a similar manner, a blank solution (10 mL) was also prepared using the actinometer solution stored in dark.
c) Absorbance of the actinometer solution after complexation with 1,10-phenanthroline at λ = 510 nm was measured by UV/Vis spectrophotometry.
d) According to Beer's law, the number of moles of Fe^{2+} formed (x) for each sample was determined by:

$$Fe^{2+} = \frac{v_1 v_3 \Delta A(510\ nm)}{10^3 v_2 l \varepsilon(510\ nm)}$$

Where:

v1 = Irradiated volume (1 mL).

v2 = The aliquot of the irradiated solution taken for the estimation of Fe+ ions (1 mL).

v3 = Final volume of the solution after complexation with 1,10-phenanthroline (10 mL).

ε(510 nm) = Molar extinction coefficient of $[Fe(Phen)_3]^{2+}$ complex (11100 L $mol^{-1}cm^{-1}$).

l = Optical path-length of the cuvette (1 cm)

ΔA(510 nm) = Difference in absorbance between the irradiated solution and the solution stored in dark (blank)

e) The number of moles of Fe^{2+} formed (x) was plotted as a function of time (t) (Figure S4). The slope (dx/dt) of the line is equal to the number of moles of Fe^{2+} formed per unit time.

f) This slope (dx/dt) was correlated to the number of moles of incident photons per unit time (F = photon flux) by using following equation:

$$\Phi(\lambda) = \frac{\frac{dx}{dt}}{F(1 - 10^{-A(\lambda)})}$$

Φ(λ) = The quantum yield for Fe^{2+} formation at 450 nm is 0.9.[26]

g) A(λ) = Absorbance of the ferrioxalate actinometer solution at a wavelength of 450 nm, which was measured placing 1 mL of the solution in a cuvette of pathlength 1 cm by UV/Vis spectrophotometry. We obtained an absorbance value of 0.3022.

h) The determined incident photons per unit time (F) is 5.558×10^{-9} einsteins/s.

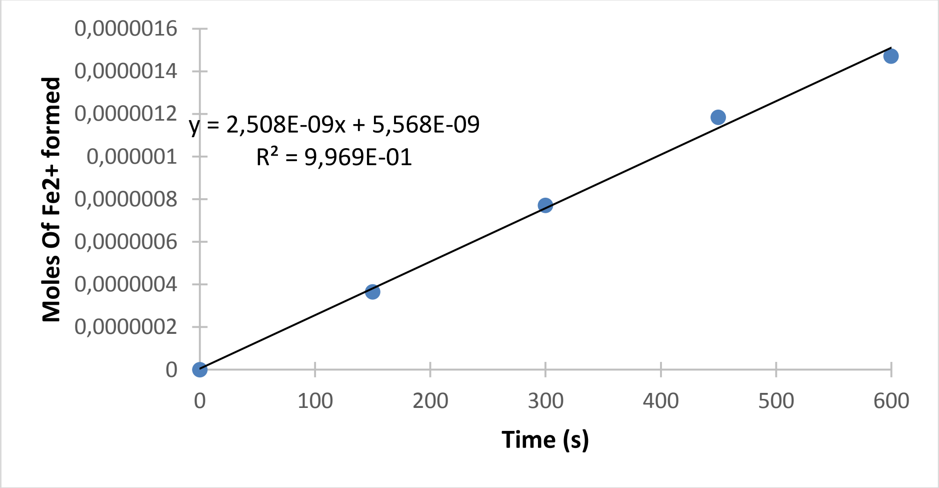

Figure S4. Moles of $[Fe(Phen)_3]^{2+}$ per unit of time formed due to decomposition of the actinometer solution at 450 nm blue Led irradiation.

i) The number of moles of the product **diphenylacetylene** formed was determined by GC (FID) analysis using mesitylene as internal standard. The measured absorbance of the reaction solution at 450 nm by UV/Vis spectrophotometer is greater than 3. Thus the number of moles of photons absorbed by the reaction mixture is roughly equal to the number of moles of incident photon per unit time (F). The number of moles of product formed was plotted against the number of moles of photon absorbed by the reaction (Figure S5). The slope of the line is equal to the quantum yield of the reaction. The calculated apparent **quantum yield** (Φ) of the reaction is **3.6**.

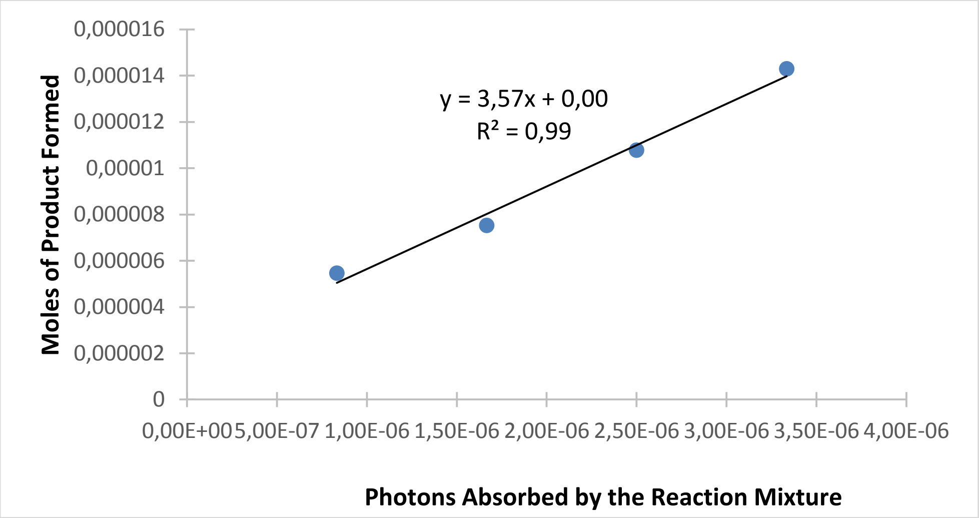

Figure S5. Moles of product formed per photon absorbed.

7. References

[1] P. Hanson, J. R. Jones, A. B. Taylor, P. H. Walton, A. W. Timms, *J. Chem. Soc., Perkin Trans. 2* **2002**, 1135-1150

[2] M. A. Ishay, Z. Lu, T. P. Yoon, *J. Am. Chem. Soc.* **2010**, *132*, 8572-8574.

[3] a) S. Sprouse, K. A. King, P. J. Spellane, R. J. Watts, *J. Am. Chem. Soc.* **1984**, *106*, 6647-6653; b) J. D. Slinker, A. A. Gorodetsky, M. S. Lowry, J. Wang, S. Parker, R. Rohl, S. Bernhard, G. G. Malliaras, *J. Am. Chem. Soc.* **2004**, *126*, 2763-2767.

[4] A. S. K. Hashmi, I. Braun, M. Rudolph, F. Rominger, *Organometallics* **2012**, *31*, 644-661.

[5] A. Collado, A. Gomez-Suarez, A. R. Martin, A. M. Z. Slawin, S. P. Nolan, *Chem. Commun.* **2013**, *49*, 5541—5543.

[6] N. Mézailles, L. Ricard, F. Gagosz, *Org. Lett.* **2005**, *7*, 4133-4136.

[7] The homocoupling product was prepared following the procedure of *Jia* and coworkers. See: X. Jia, K. Yin, C. Li, J. Lia, H. Bian, *Green Chem.* **2011**, 13, 2175–2178.

[8] A. Gogoi, A. Dewan, G. Boraha, U. Bora, *New J. Chem.* **2015**, 39, 3341-3344.

[9] C. He, J. Ke, H. Xu, A. Lei, *Angew. Chem. Int. Ed.* **2013**, *52*, 1527-1530.

[10] E. Fager-Jokela, M. Muuronen, M. Patzschke, J. Helaja, *J. Org. Chem.* **2012**, *77*, 9134-9147.

[11] D. Polyansky, E. Danilov, S. Voskresensky, M. Rodgers, D. Neckers, *J. Am. Chem. Soc.* **2005**, 127, 13452-13453.

[12] L. Melzig, A. Metzger, P. Knochel, *Chem. Eur. J.* **2011**, *17*, 2948-2956.

[13] T. Shibata, G. Nishizawa, K. Endo, *Synlett* **2008**, *5*, 765-768.

[14] M. Uemura, H. Yorimitsu, K. Oshima, *Tetrahedron* **2008**, *64*, 1829-1833.

[15] Y. Wagh, N. Asao, *J. Org. Chem.* **2015**, 80, 847−851.

[16] S. Jadhav, A. Kumbhar, S. Mali, C. Hong, R. Salunkhe, *New J. Chem.* **2015**, 39, 2333--2341

[17] S. Takahashi, Y. Kuroyama, K. Sonogashira, N. Hagihara, *Synthesis* **1980**, 627-629.

[18] Y. Nishihara, E. Inoue, S. Noyori, D. Ogawa, Y. Okada, M. Iwasaki, K. Takagi, *Tetrahedron* **2012**, 4869-4881.

[19] K. Kondo, T. Fujitanib, N. Ohnishi, *J. Mater. Chem.* **1997**, 429–433.

[20] M. Corpet, X. Bai, C. Gosmini, *Adv. Synth. Catal* **2014**, 356, 2937 – 2942

[21] J. Kim, D. Lee, B. Jun, Y. Lee, *Tetrahedron Letters* **2007**, 7079-7084.

[22] W. Tadros, A. B. Badie, M. S. Ishak, *J. Chem. Soc.* **1958**, 4110-4112.

[23] Y. Miao, A. Dupé, C. Bruneau, C. Fischmeister, *Eur. J. Org. Chem.* **2014**, 5071-5077.

[24] X. Shen, D. M. Ho, R. A. Pascal Jr., *J. Am. Chem. Soc.* **2004**, *126*, 5798-5805.

[25] Ł. Wozniak, J. J. Murphy, P. Melchiorre, *J. Am. Chem. Soc.* **2015**, 137, 5678-5681.

[26] S. R. Kandukuri, A. Bahamonde, I. Chatterjee, I. D. Jurberg, E. C. Escudero-Adán, P. Melchiorre Angew. *Chem. Int. Ed.* **2015**, 54, 1485-1489.

8. NMR Spectra of the Cross-Coupled Products

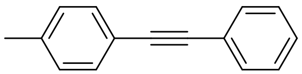

3aa

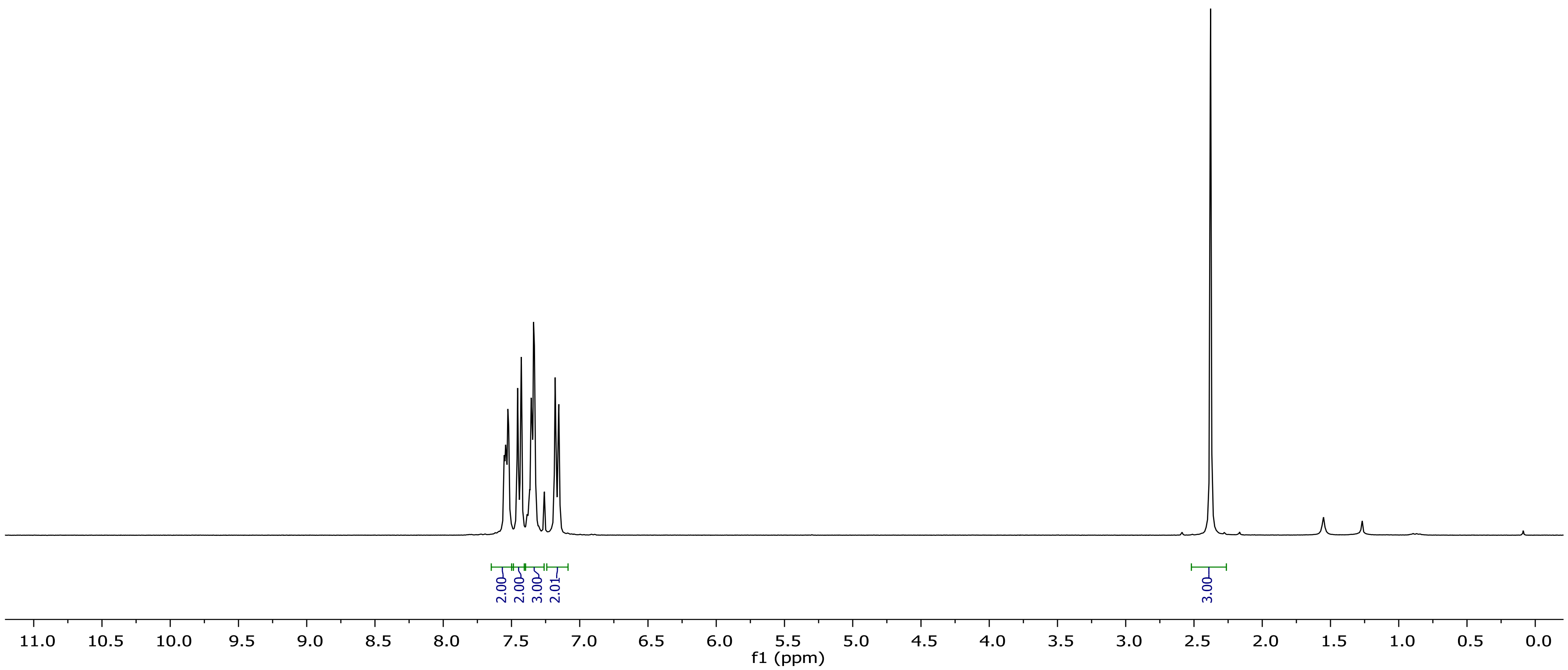

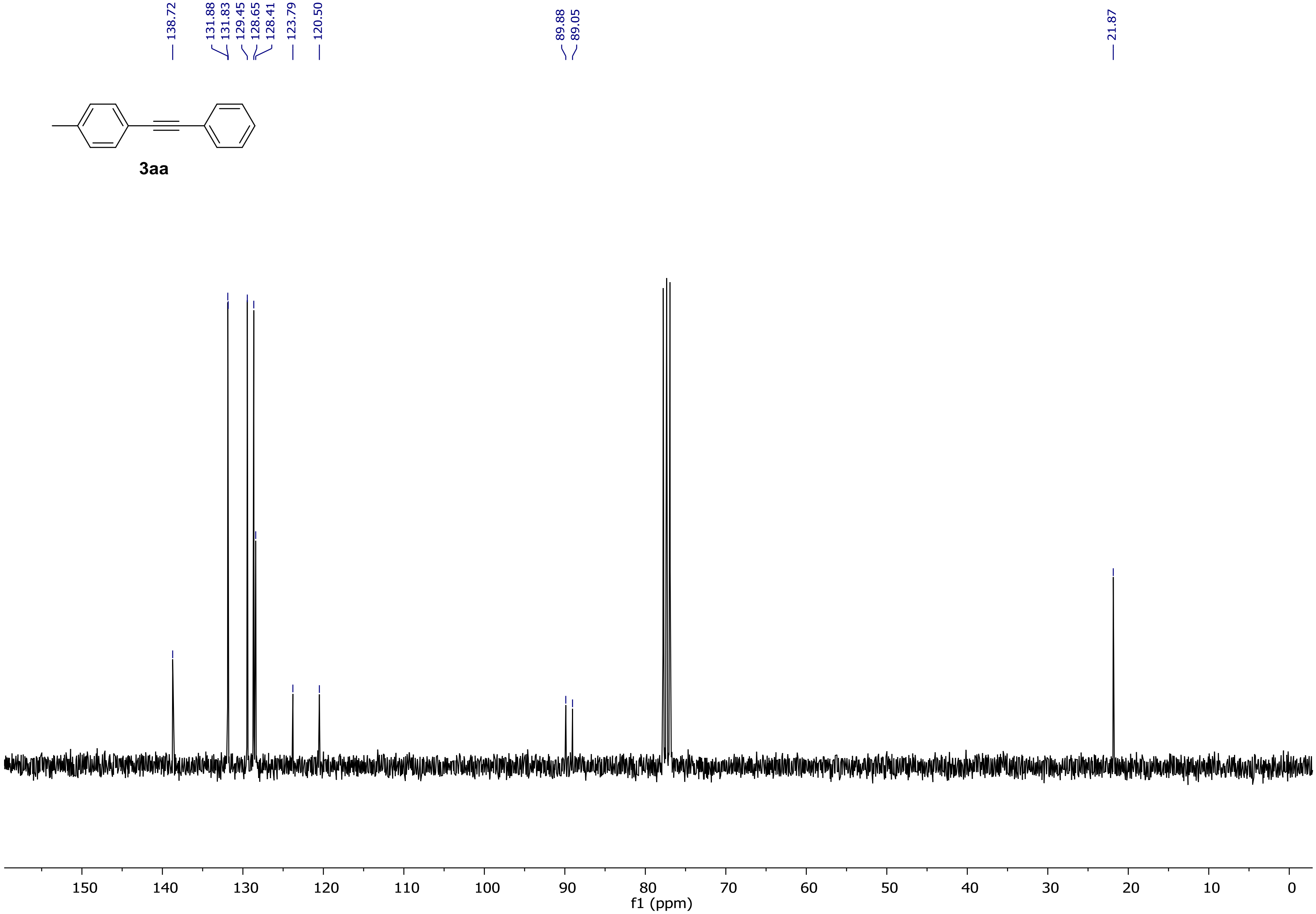

138.72
131.88
131.83
129.45
128.65
128.41
123.79
120.50
89.88
89.05
21.87
3aa
150
140
130
120
110
100
90
80
70
60
50
40
30
20
10
0
f1 (ppm)

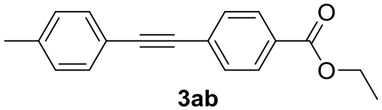

3ab

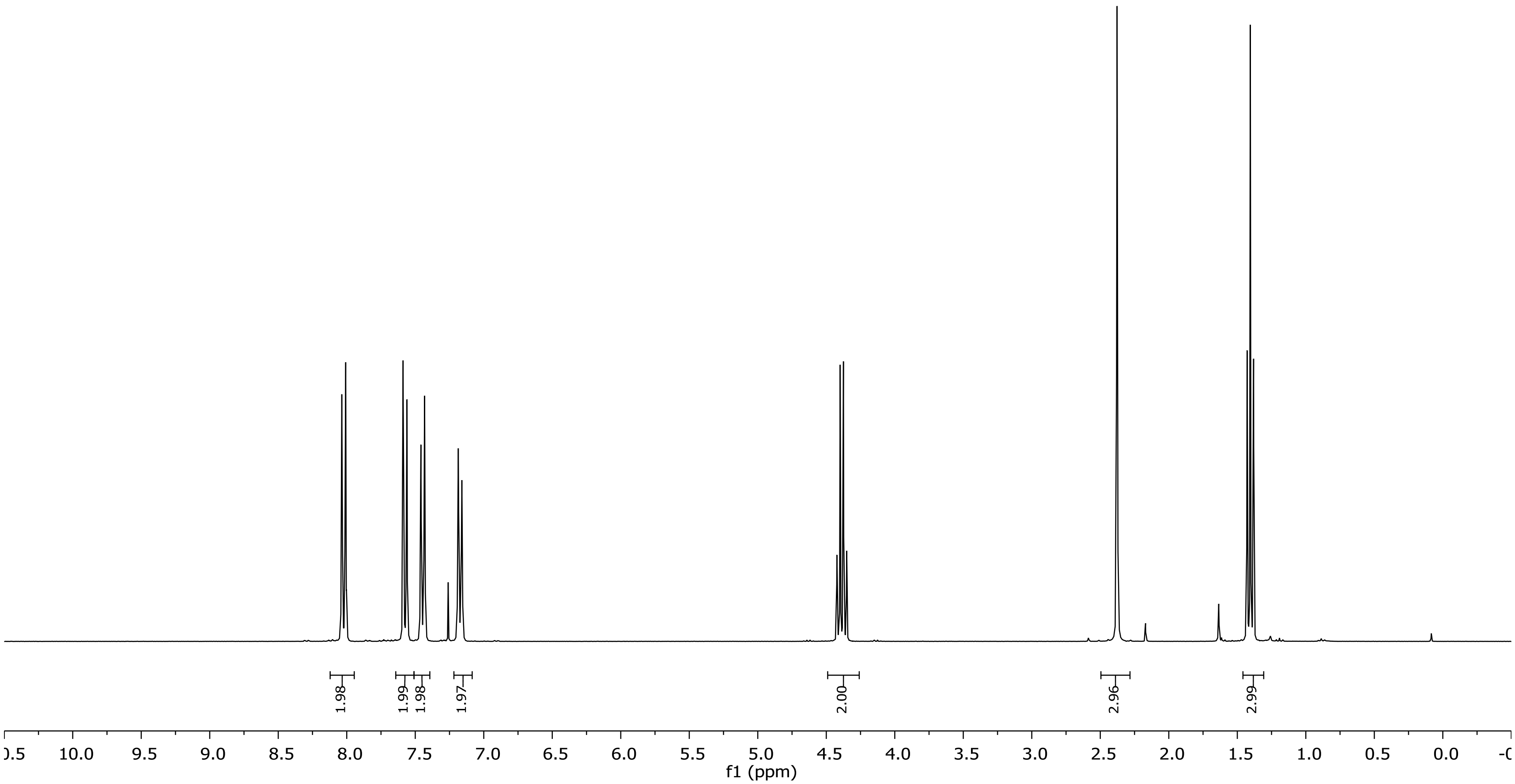

1.98
1.99
1.98
1.97
2.00
2.96
2.99
0.5 10.0 9.5 9.0 8.5 8.0 7.5 7.0 6.5 6.0 5.5 5.0 4.5 4.0 3.5 3.0 2.5 2.0 1.5 1.0 0.5 0.0
f1 (ppm)

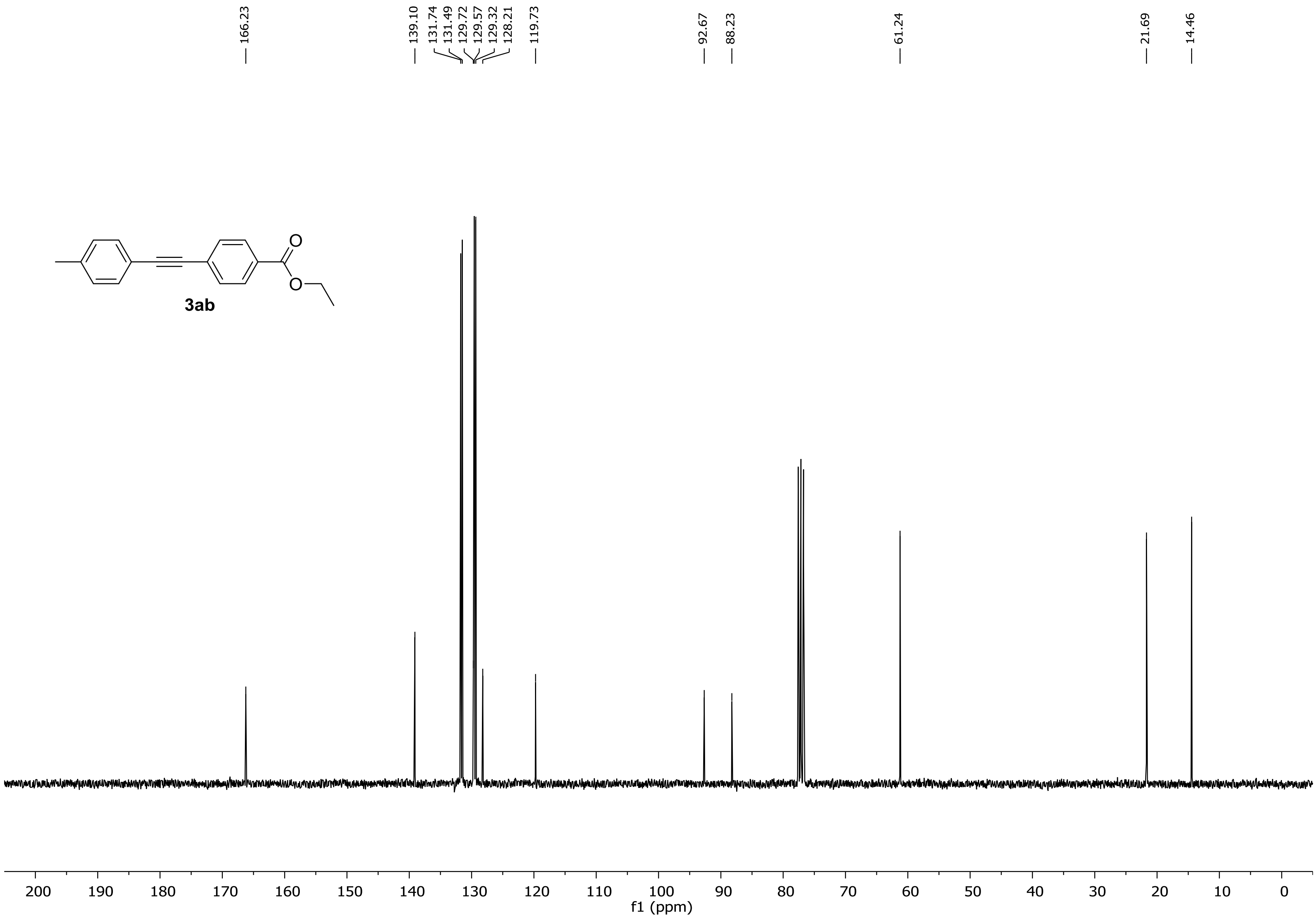
166.23
139.10
131.74
131.49
129.72
129.57
129.32
128.21
119.73
92.67
88.23
61.24
21.69
14.46
O
O
3ab
200
190
180
170
160
150
140
130
120
110
100
90
80
70
60
50
40
30
20
10
0
f1 (ppm)

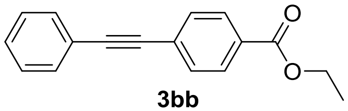

3bb

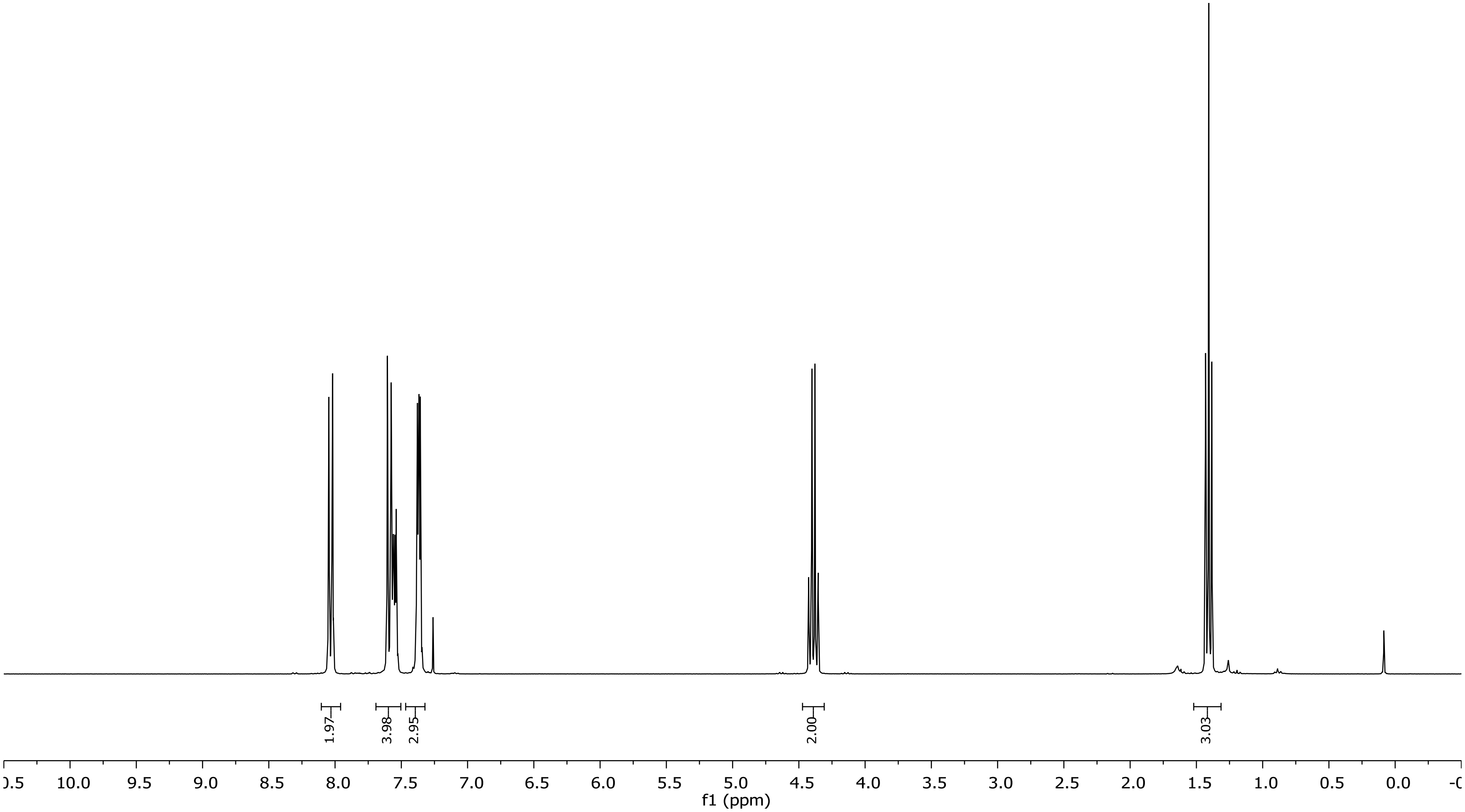
1.97
3.98
2.95
2.00
3.03
0.5
10.0
9.5
9.0
8.5
8.0
7.5
7.0
6.5
6.0
5.5
5.0
4.5
4.0
3.5
3.0
2.5
2.0
1.5
1.0
0.5
0.0
f1 (ppm)

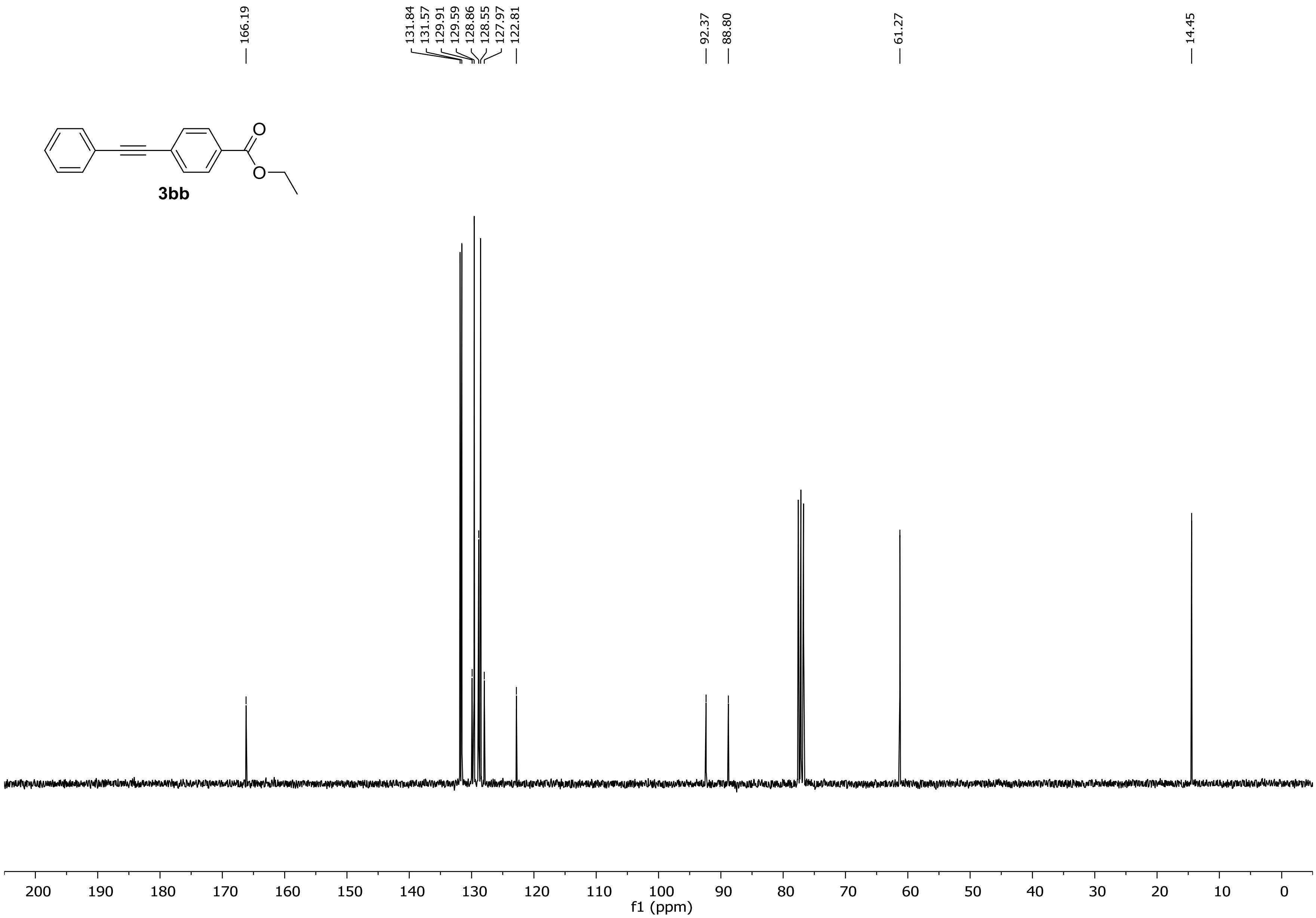
166.19
131.84
131.57
129.91
129.59
128.86
128.55
127.97
122.81
92.37
88.80
61.27
14.45
3bb
200 190 180 170 160 150 140 130 120 110 100 90 80 70 60 50 40 30 20 10 0
f1 (ppm)

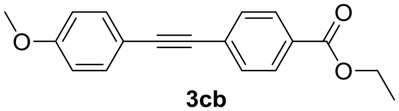

3cb

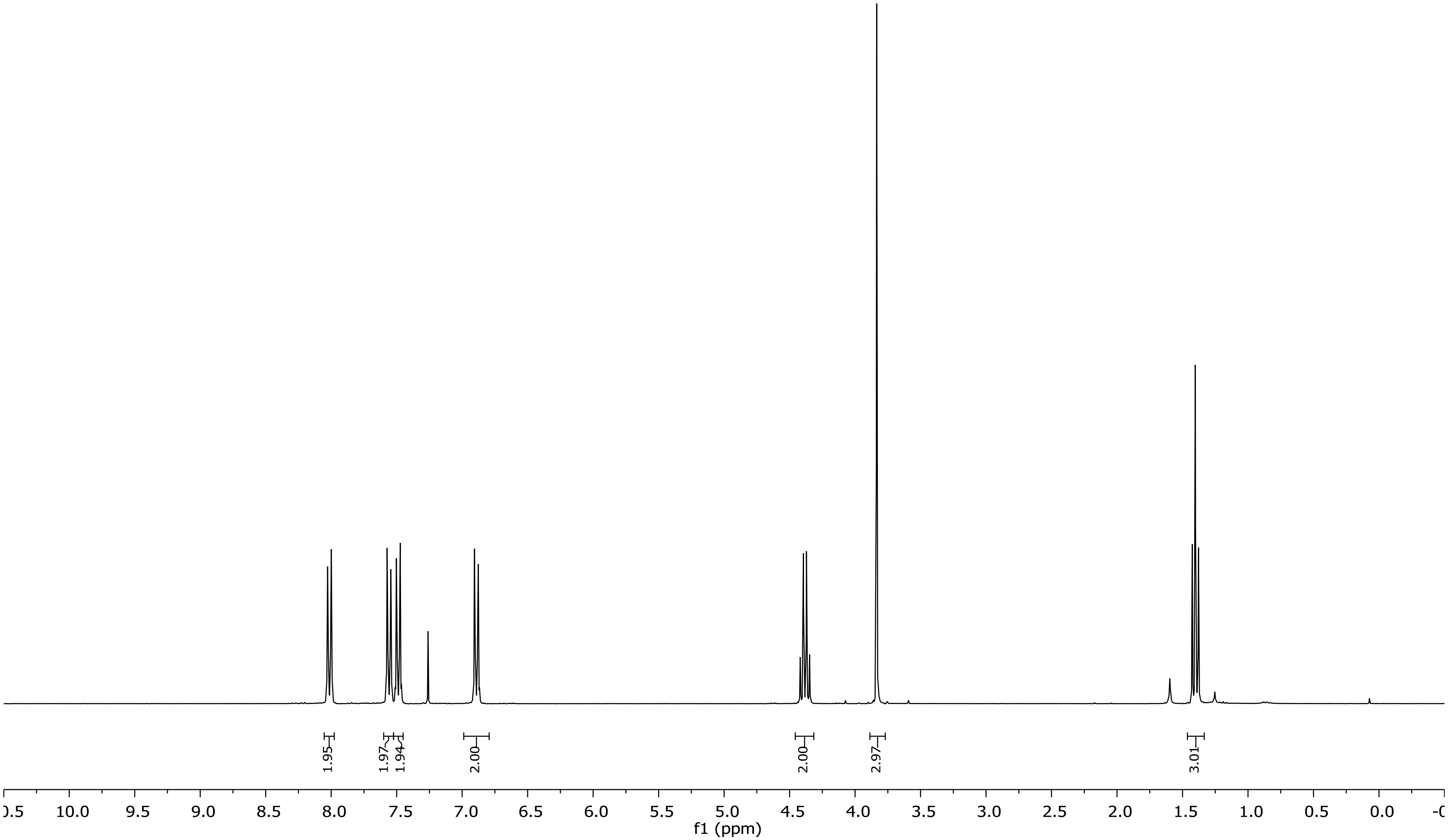

1.95
1.97
1.94
2.00
2.00
2.97
3.01
0.5
10.0
9.5
9.0
8.5
8.0
7.5
7.0
6.5
6.0
5.5
5.0
4.5
4.0
3.5
3.0
2.5
2.0
1.5
1.0
0.5
0.0
f1 (ppm)

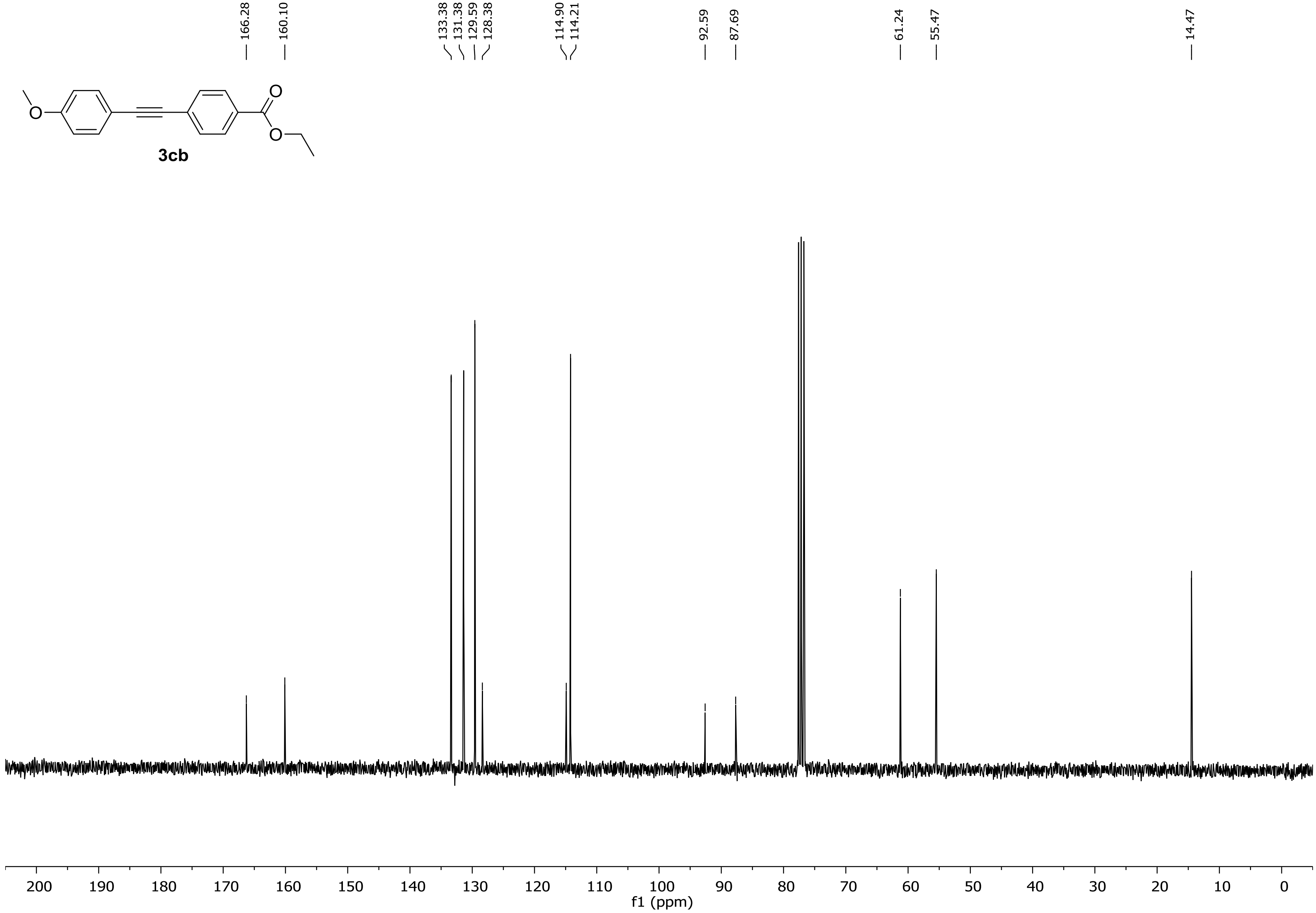
166.28
160.10
133.38
131.38
129.59
128.38
114.90
114.21
92.59
87.69
61.24
55.47
14.47
O
O
O
3cb
200 190 180 170 160 150 140 130 120 110 100 90 80 70 60 50 40 30 20 10 0
f1 (ppm)

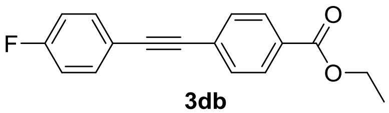

3db

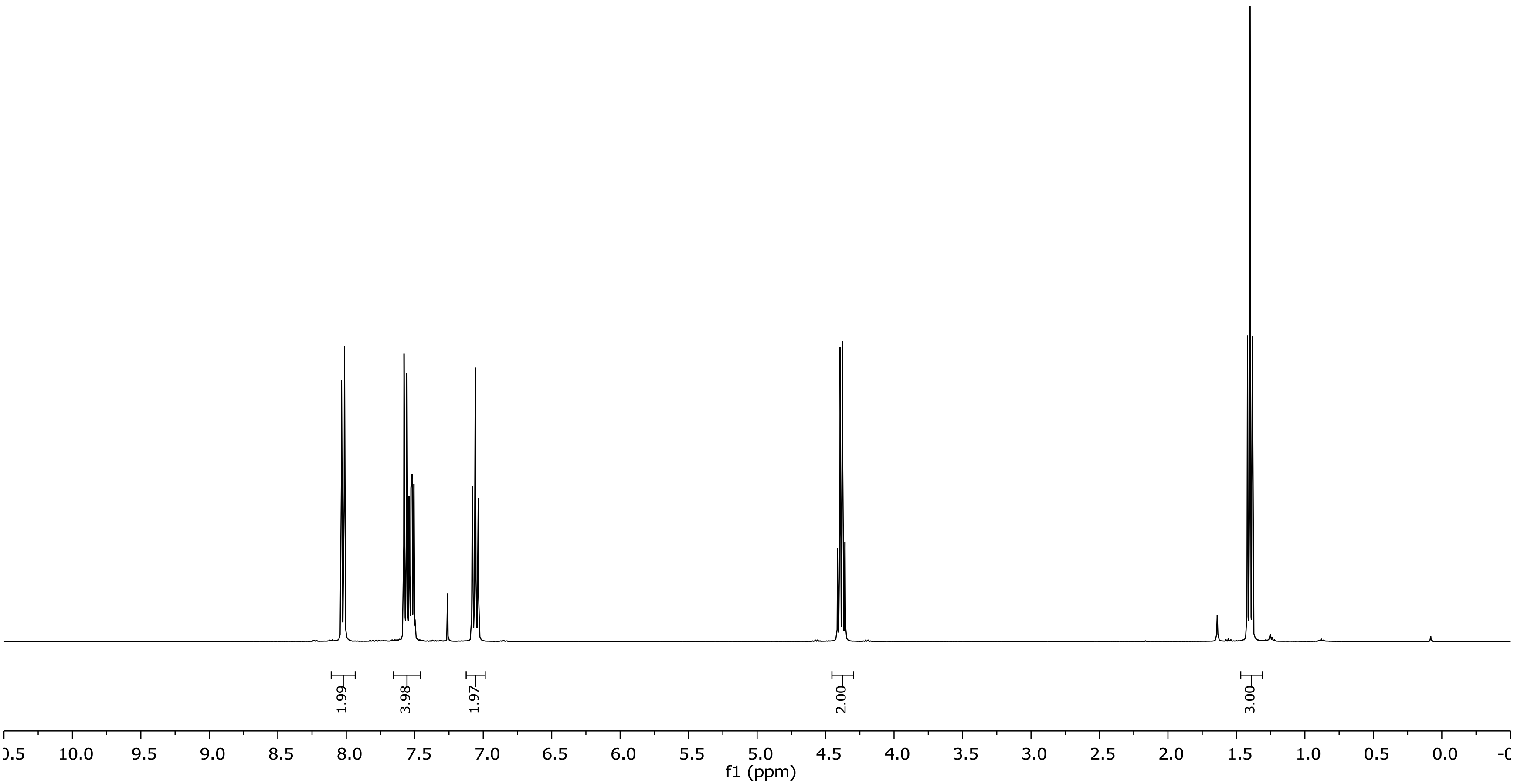
1.99
3.98
1.97
2.00
3.00
10.0
9.5
9.0
8.5
8.0
7.5
7.0
6.5
6.0
5.5
5.0
4.5
4.0
3.5
3.0
2.5
2.0
1.5
1.0
0.5
0.0
f1 (ppm)

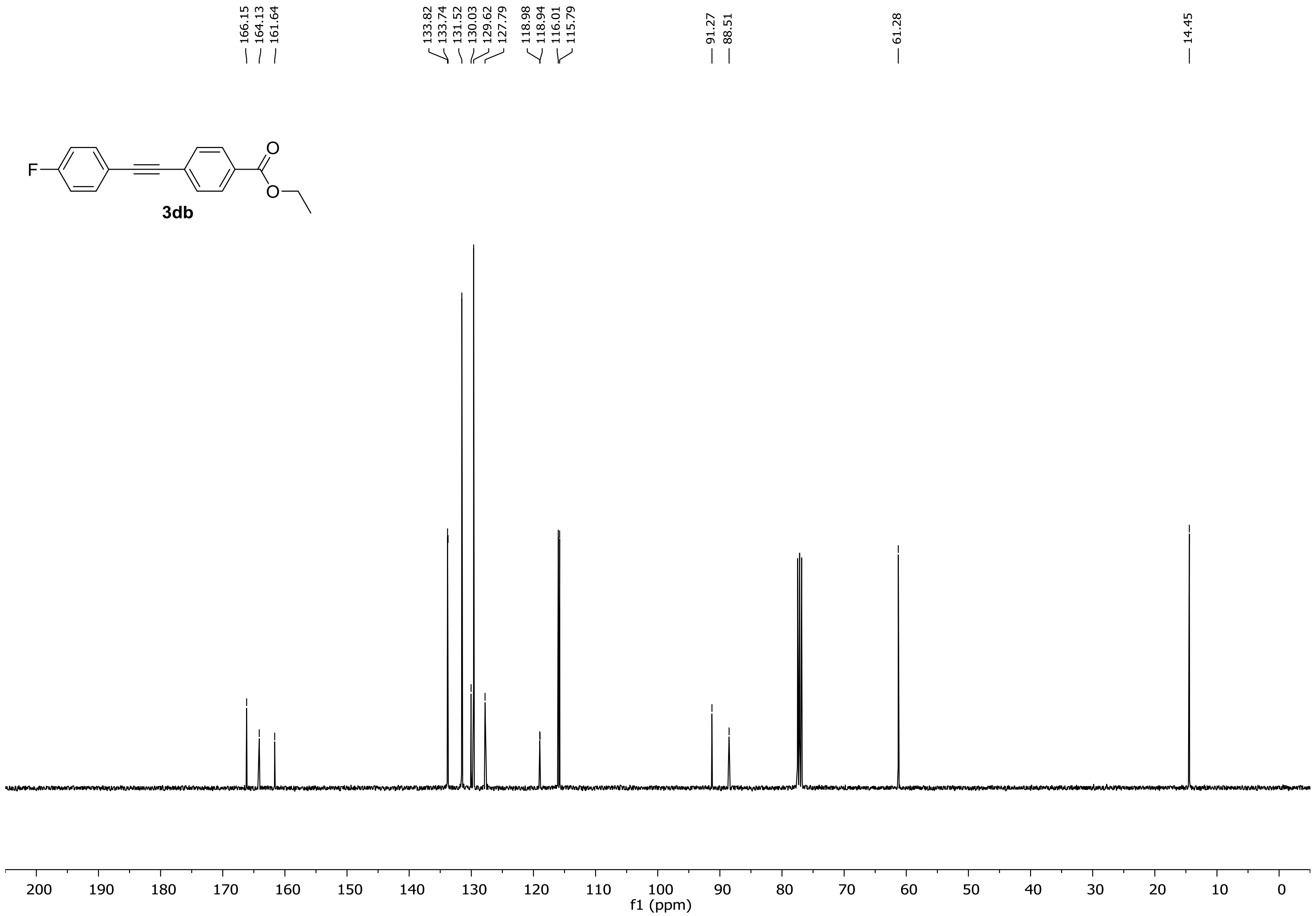
166.15
164.13
161.64
133.82
133.74
131.52
130.03
129.62
127.79
118.98
118.94
116.01
115.79
91.27
88.51
61.28
14.45
F
O
O
3db
200
190
180
170
160
150
140
130
120
110
100
90
80
70
60
50
40
30
20
10
0
f1 (ppm)

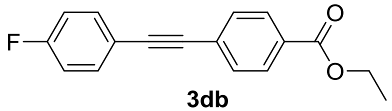

3db

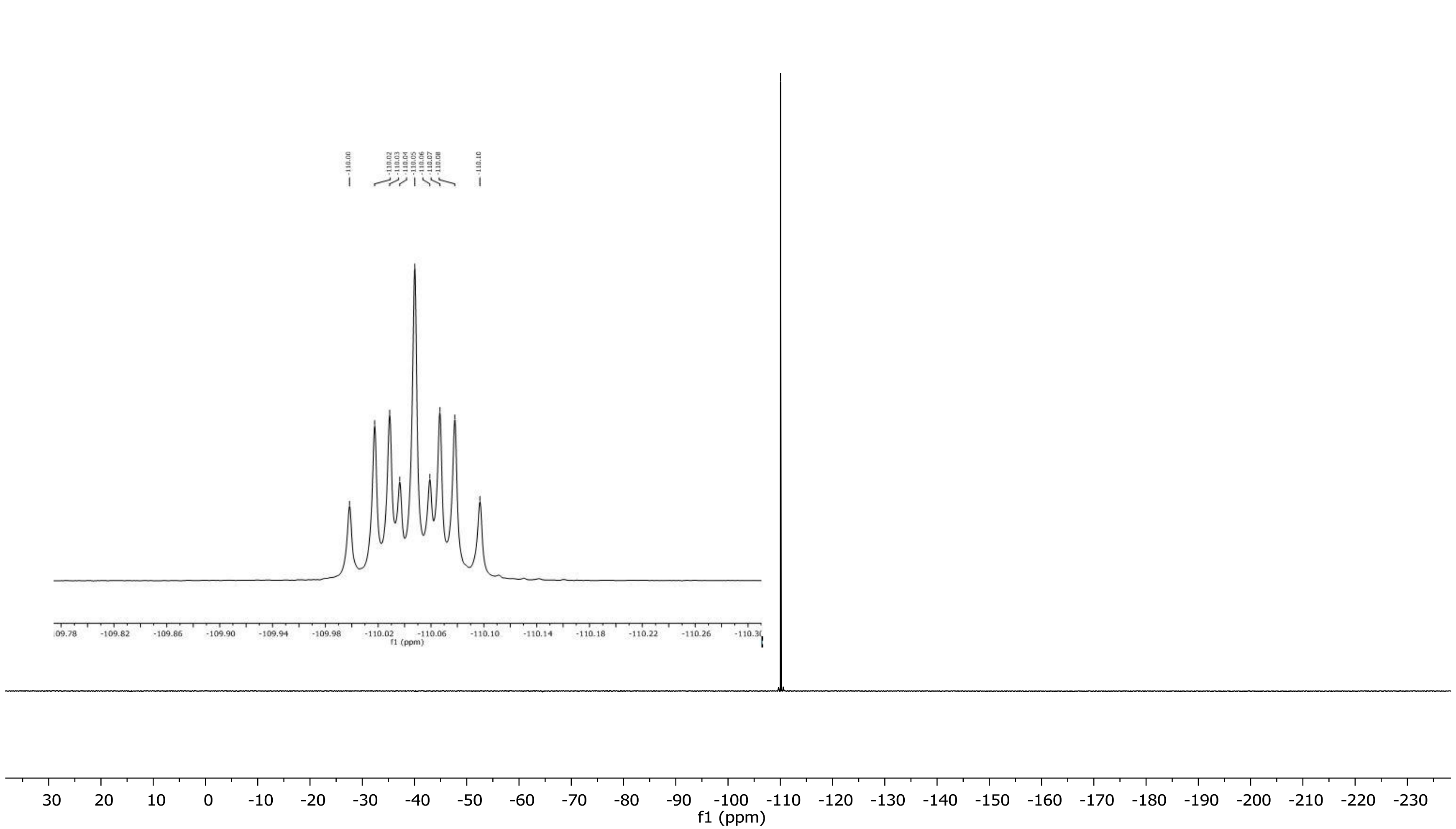

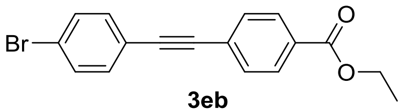
Br
O
O

3eb

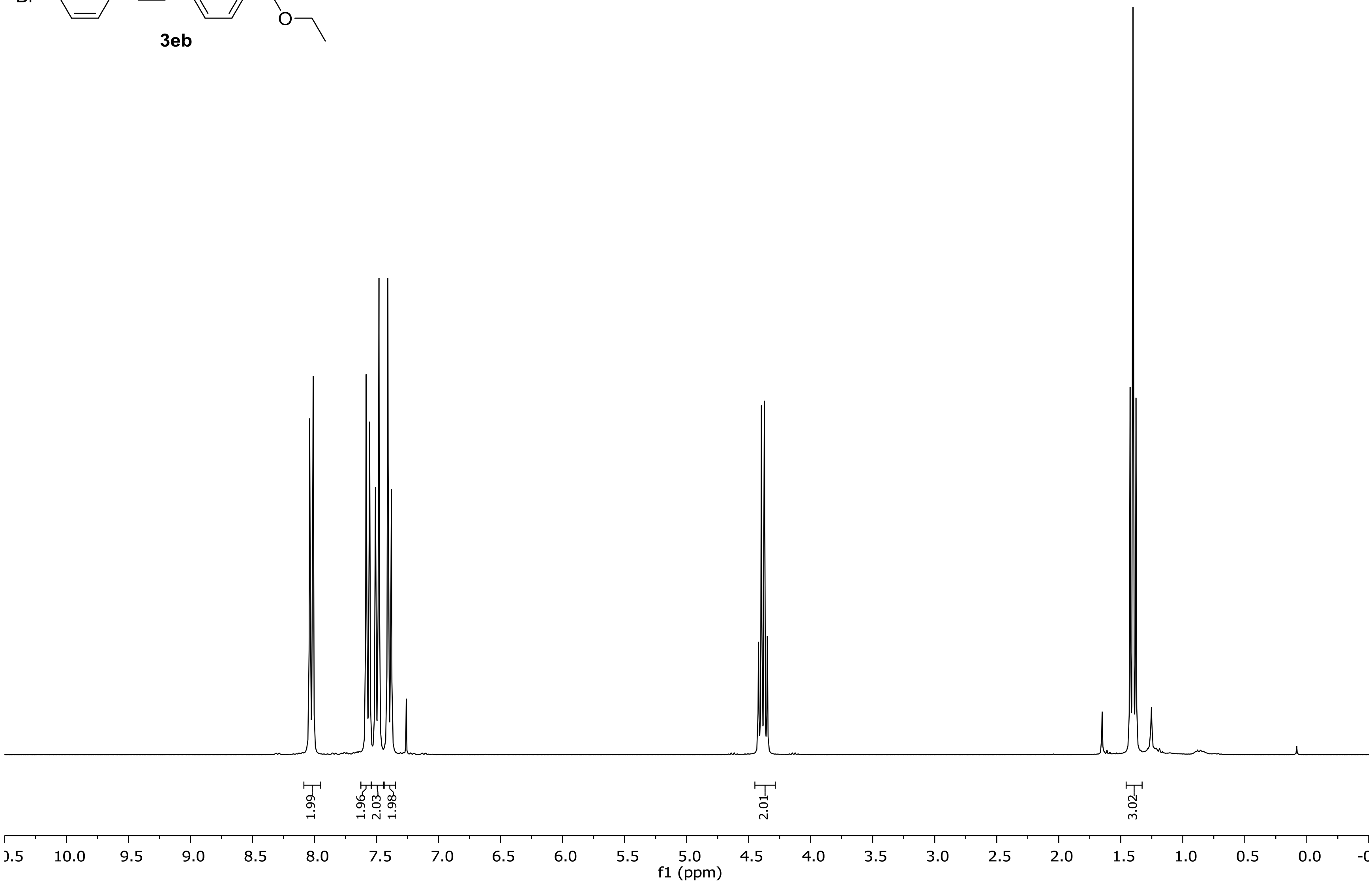
1.99
1.96
2.03
1.98
2.01
3.02
10.0 9.5 9.0 8.5 8.0 7.5 7.0 6.5 6.0 5.5 5.0 4.5 4.0 3.5 3.0 2.5 2.0 1.5 1.0 0.5 0.0
f1 (ppm)

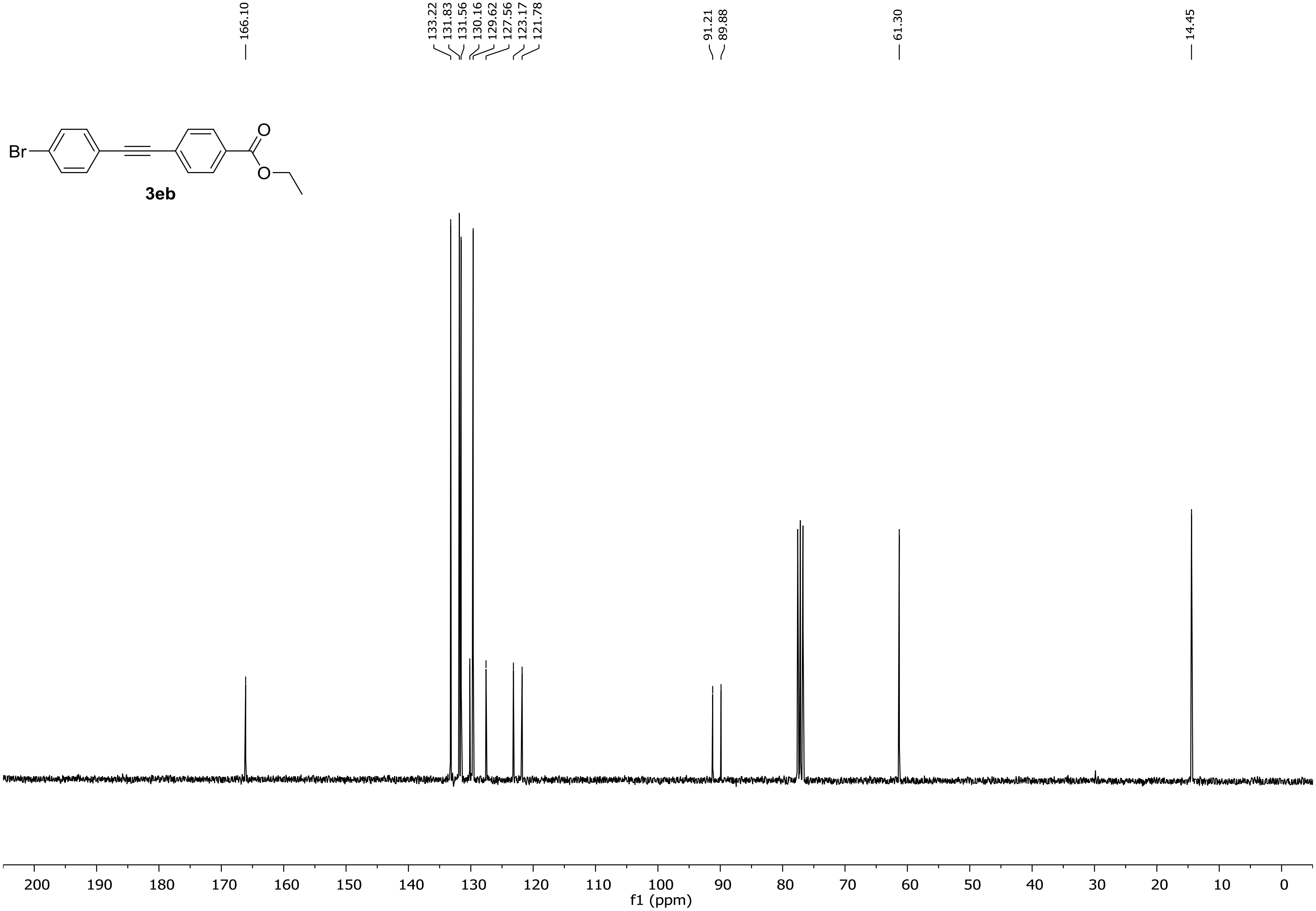

166.10
133.22
131.83
131.56
130.16
129.62
127.56
123.17
121.78
91.21
89.88
61.30
14.45
Br
O
O
3eb
200 190 180 170 160 150 140 130 120 110 100 90 80 70 60 50 40 30 20 10 0
f1 (ppm)

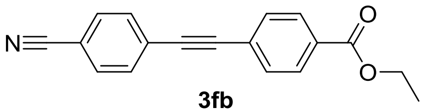
N
O
O

3fb

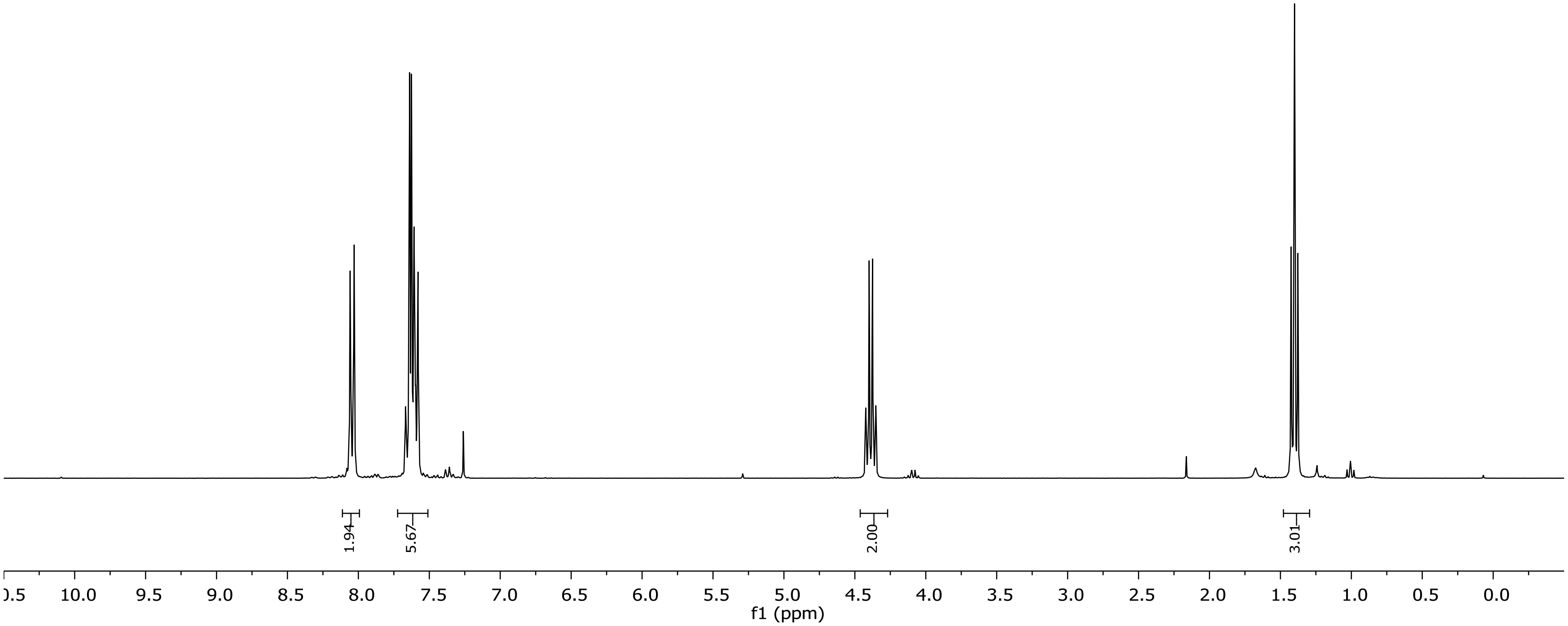
1.94
5.67
2.00
3.01
).5 10.0 9.5 9.0 8.5 8.0 7.5 7.0 6.5 6.0 5.5 5.0 4.5 4.0 3.5 3.0 2.5 2.0 1.5 1.0 0.5 0.0
f1 (ppm)

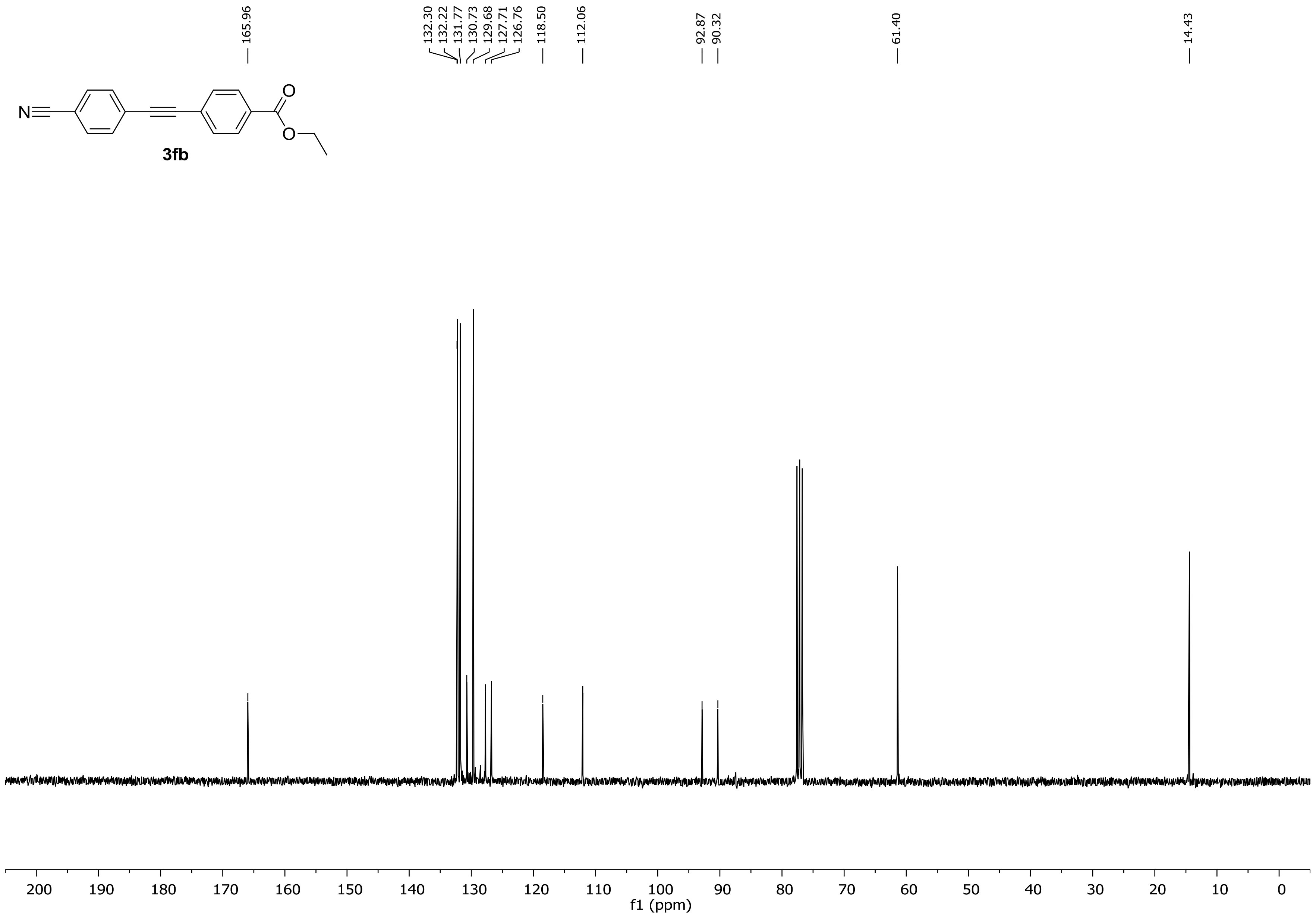
165.96
132.30
132.22
131.77
130.73
129.68
127.71
126.76
118.50
112.06
92.87
90.32
61.40
14.43
N
O
O
3fb
200 190 180 170 160 150 140 130 120 110 100 90 80 70 60 50 40 30 20 10 0
f1 (ppm)

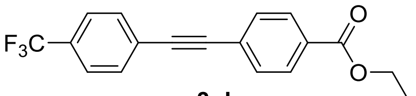
F3C
O
O

3gb

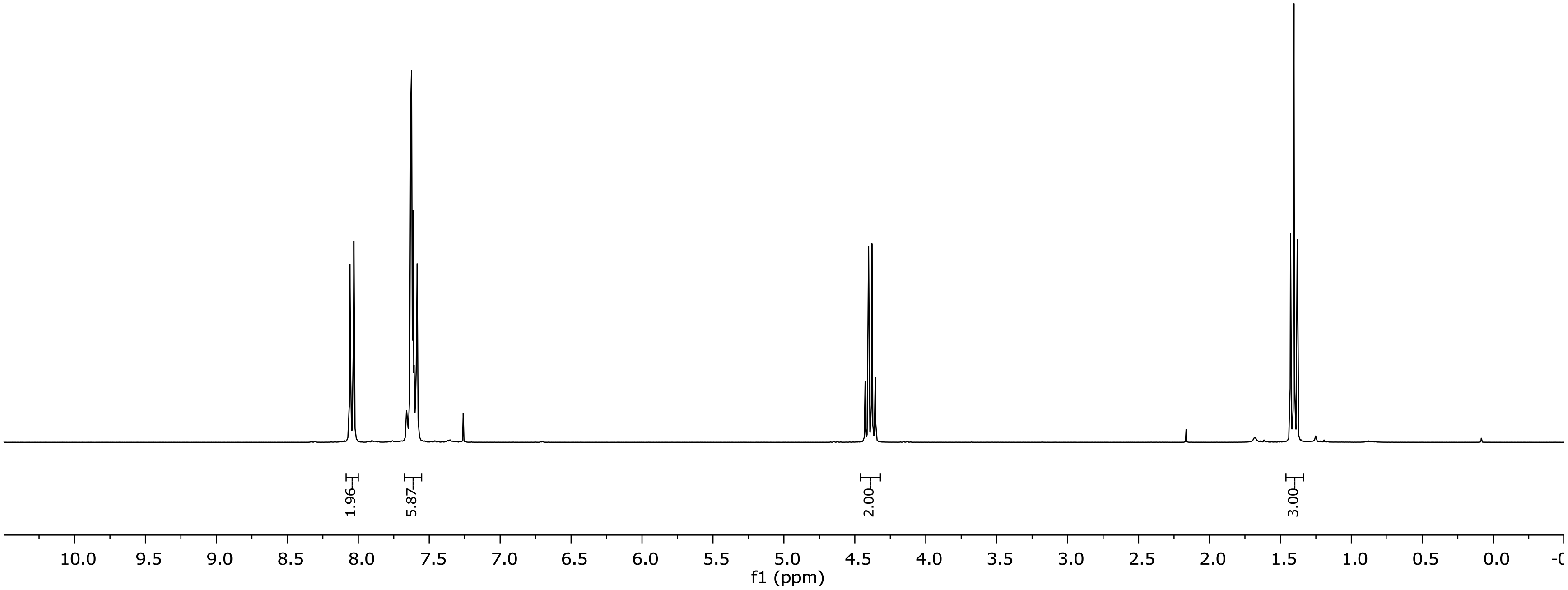
1.96
5.87
2.00
3.00
10.0
9.5
9.0
8.5
8.0
7.5
7.0
6.5
6.0
5.5
5.0
4.5
4.0
3.5
3.0
2.5
2.0
1.5
1.0
0.5
0.0
f1 (ppm)

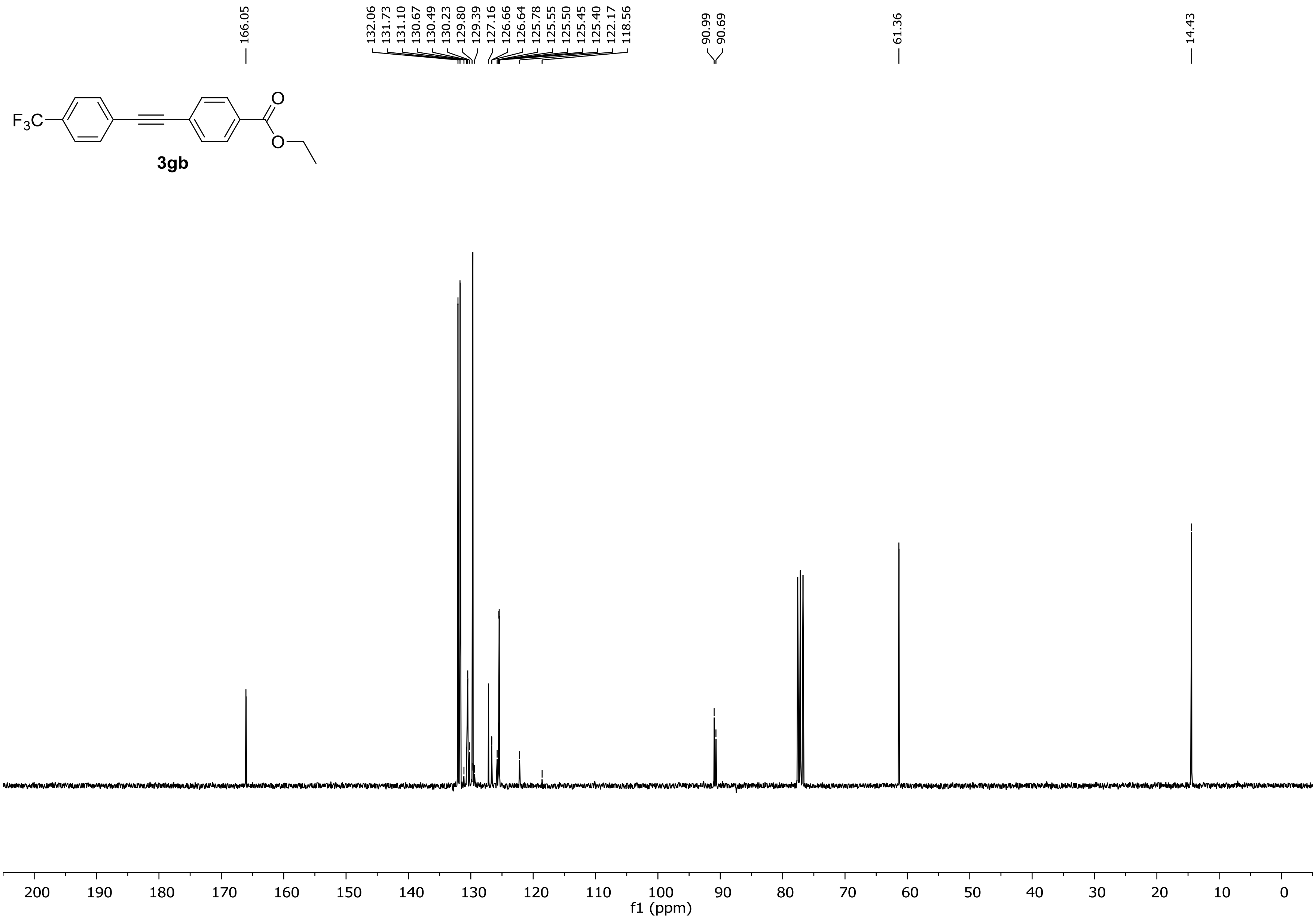
166.05
132.06
131.73
131.10
130.67
130.49
130.23
129.80
129.39
127.16
126.66
126.64
125.78
125.55
125.50
125.45
125.40
122.17
118.56
90.99
90.69
61.36
14.43
F3C
O
O
3gb
200
190
180
170
160
150
140
130
120
110
100
90
80
70
60
50
40
30
20
10
0
f1 (ppm)

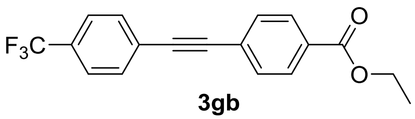

3gb

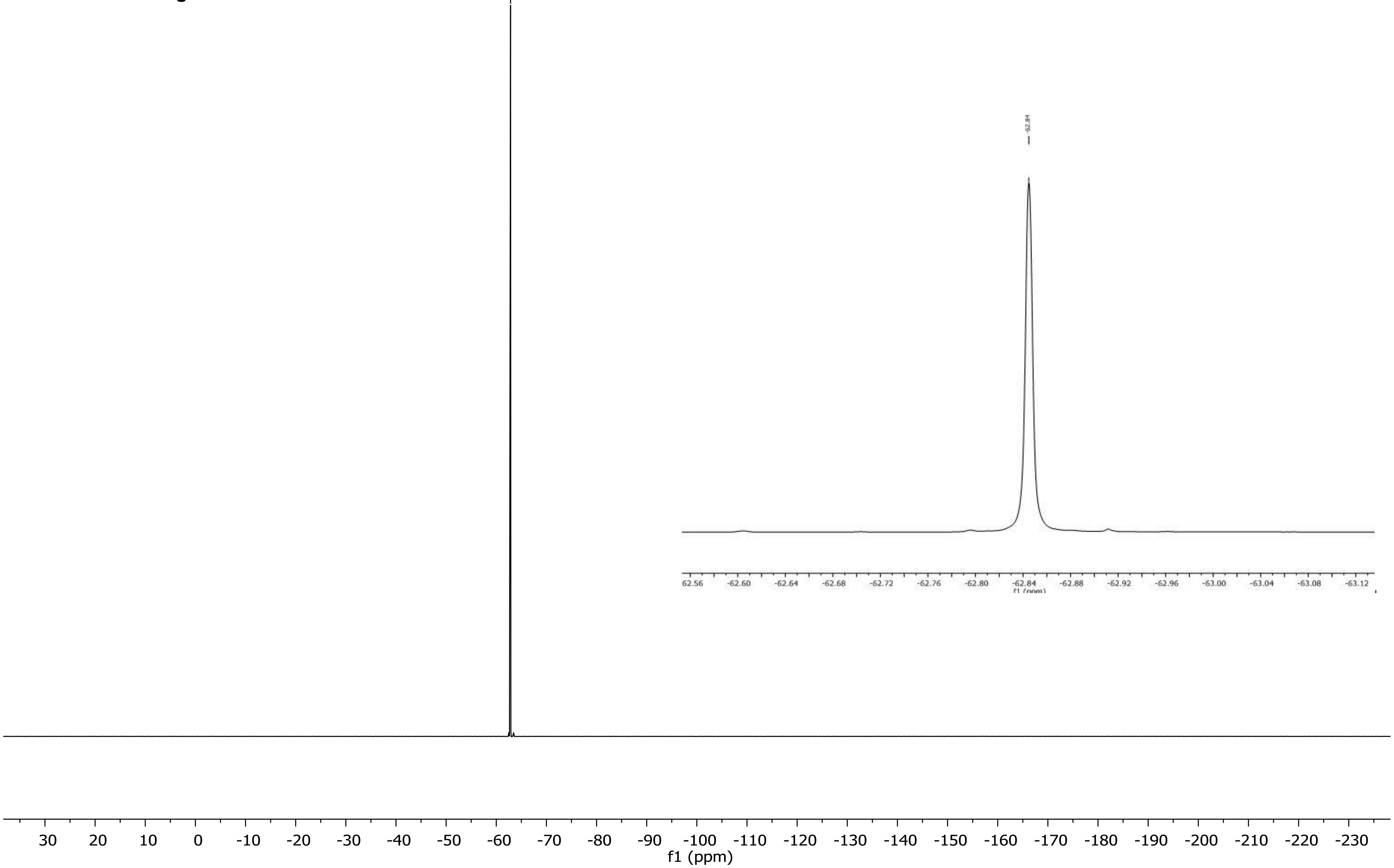

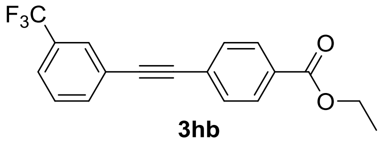

3hb

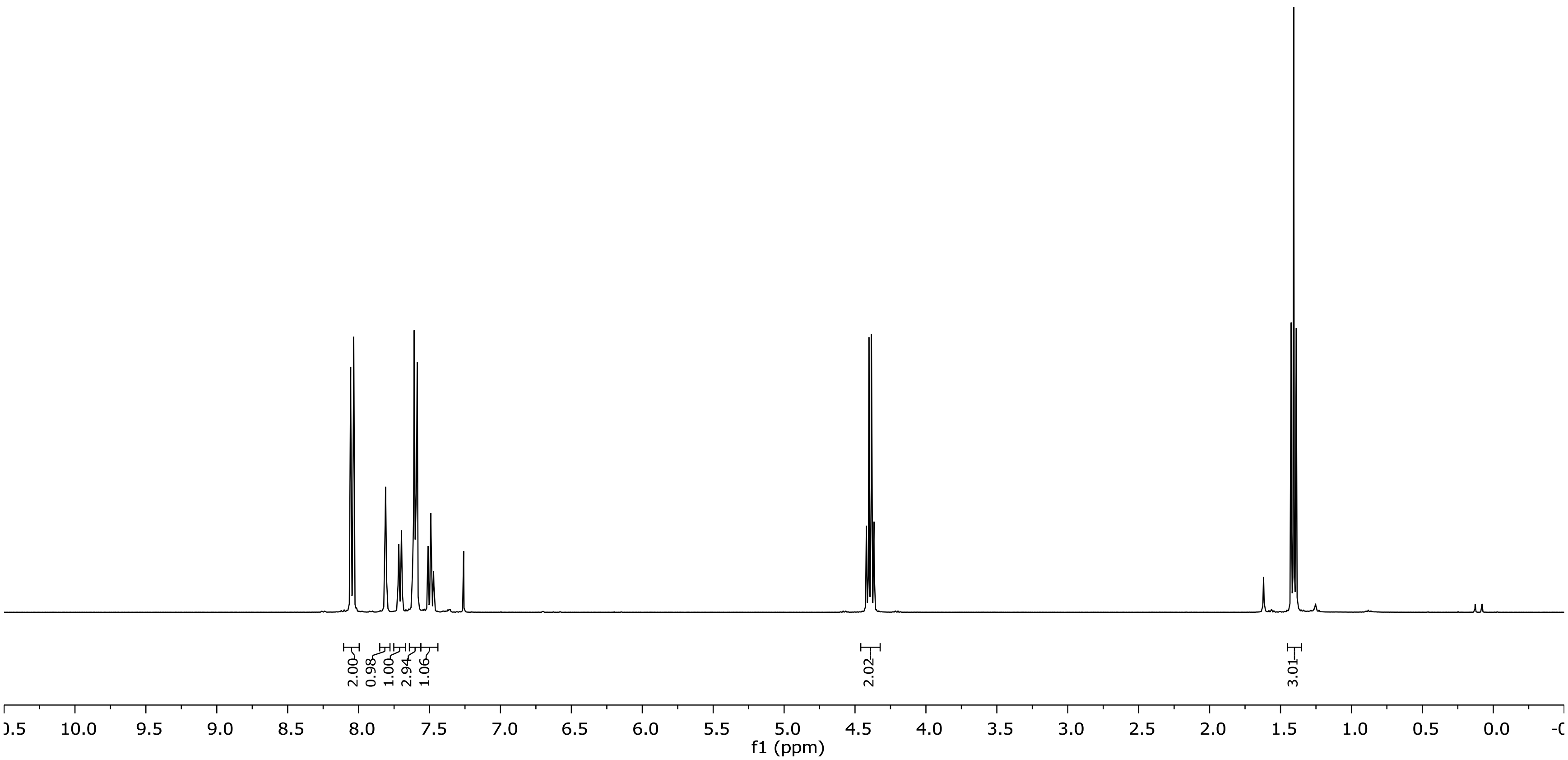

2.00
0.98
1.00
2.94
1.06
2.02
3.01
10.0
9.5
9.0
8.5
8.0
7.5
7.0
6.5
6.0
5.5
5.0
4.5
4.0
3.5
3.0
2.5
2.0
1.5
1.0
0.5
0.0
f1 (ppm)

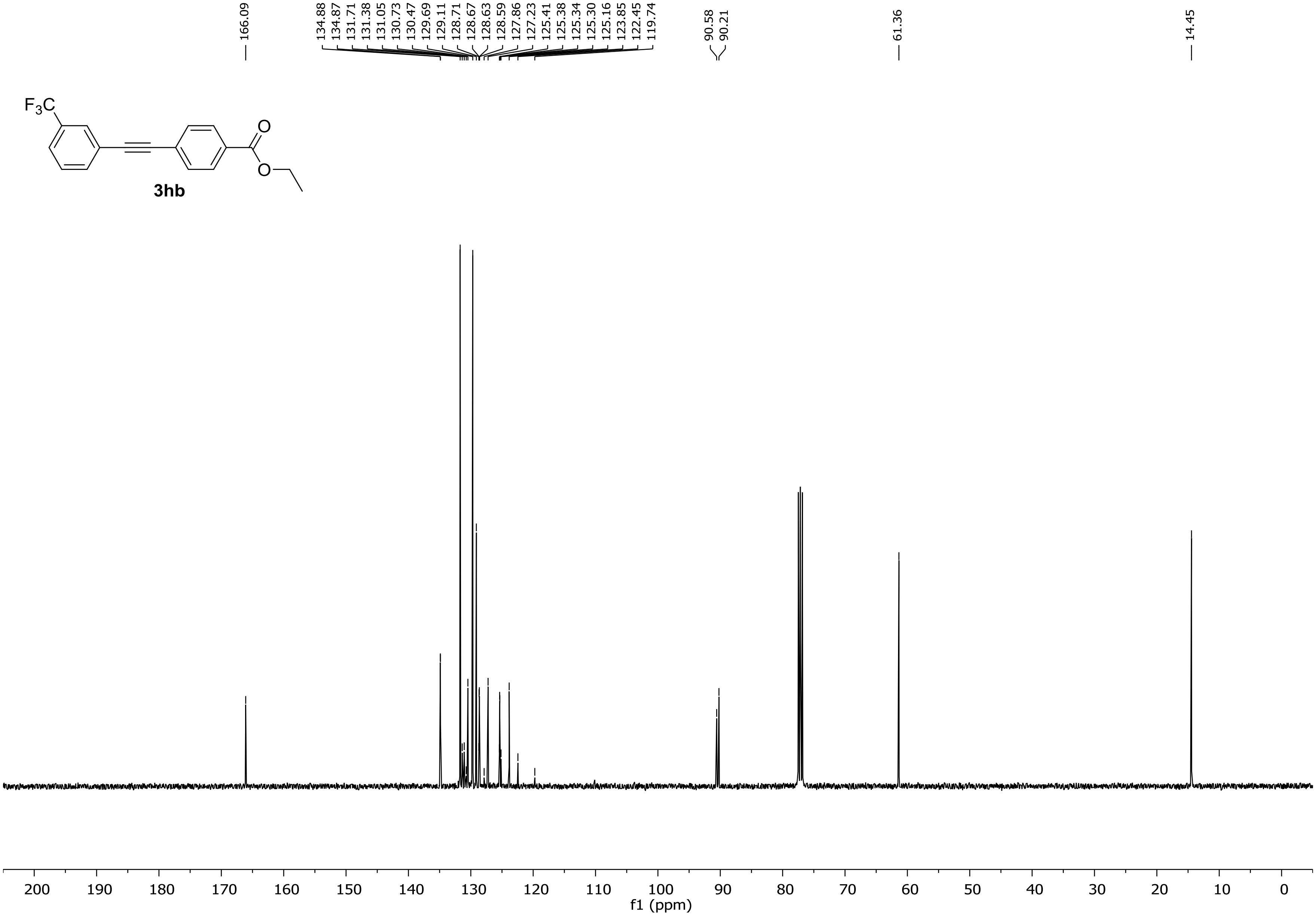
166.09
134.88
134.87
131.71
131.38
131.05
130.73
130.47
129.69
129.11
128.71
128.67
128.63
128.59
127.86
127.23
125.41
125.38
125.34
125.30
125.16
123.85
122.45
119.74
90.58
90.21
61.36
14.45
F3C
O
O
3hb
200 190 180 170 160 150 140 130 120 110 100 90 80 70 60 50 40 30 20 10 0
f1 (ppm)

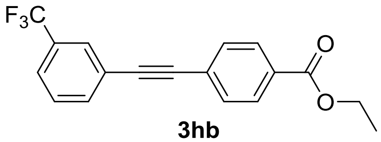
F3C
O
O

3hb

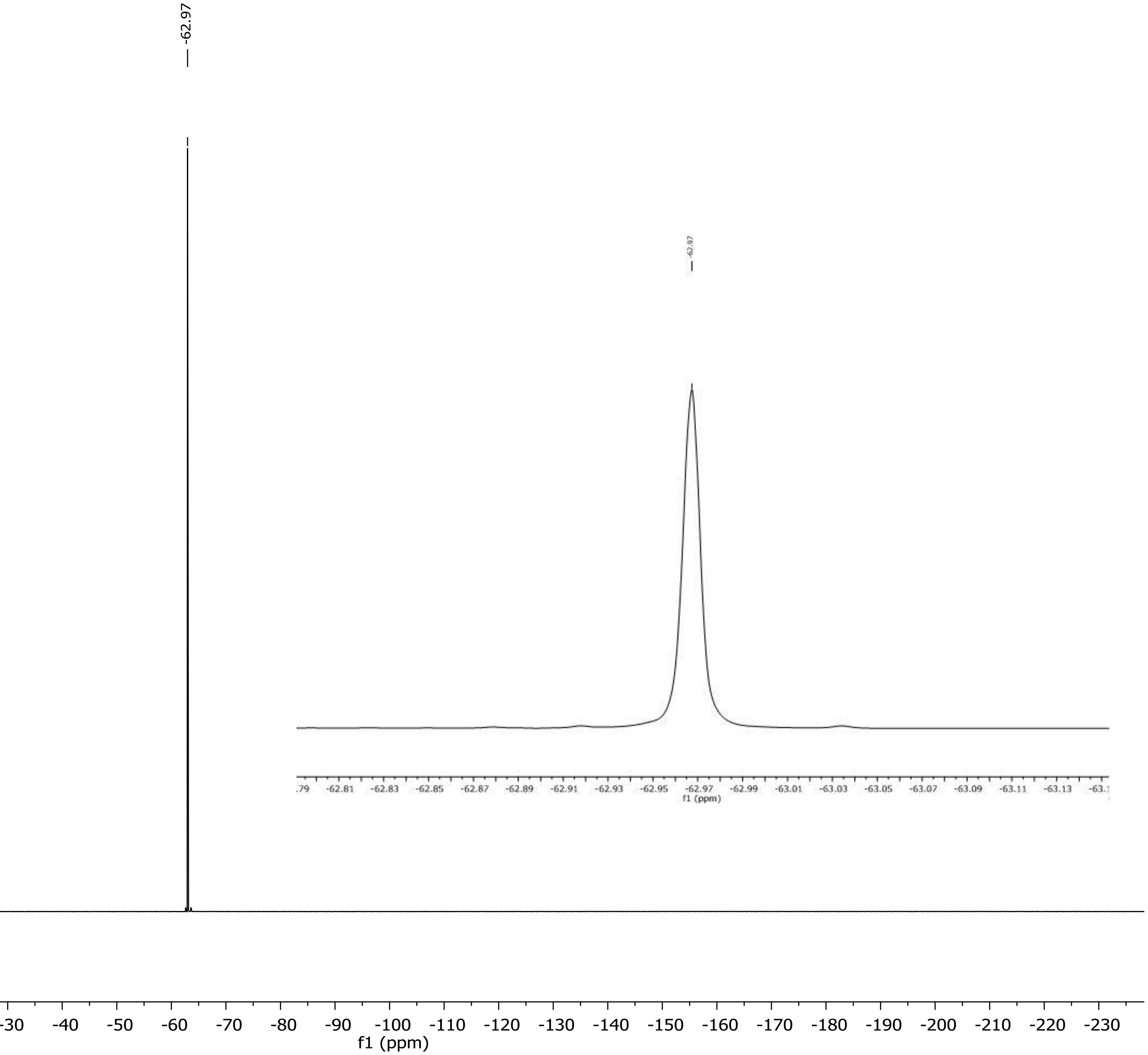
-62.97
-62.97
30 20 10 0 -10 -20 -30 -40 -50 -60 -70 -80 -90 -100 -110 -120 -130 -140 -150 -160 -170 -180 -190 -200 -210 -220 -230
f1 (ppm)

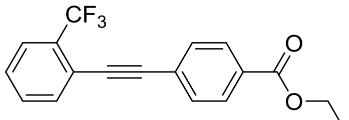

3ib

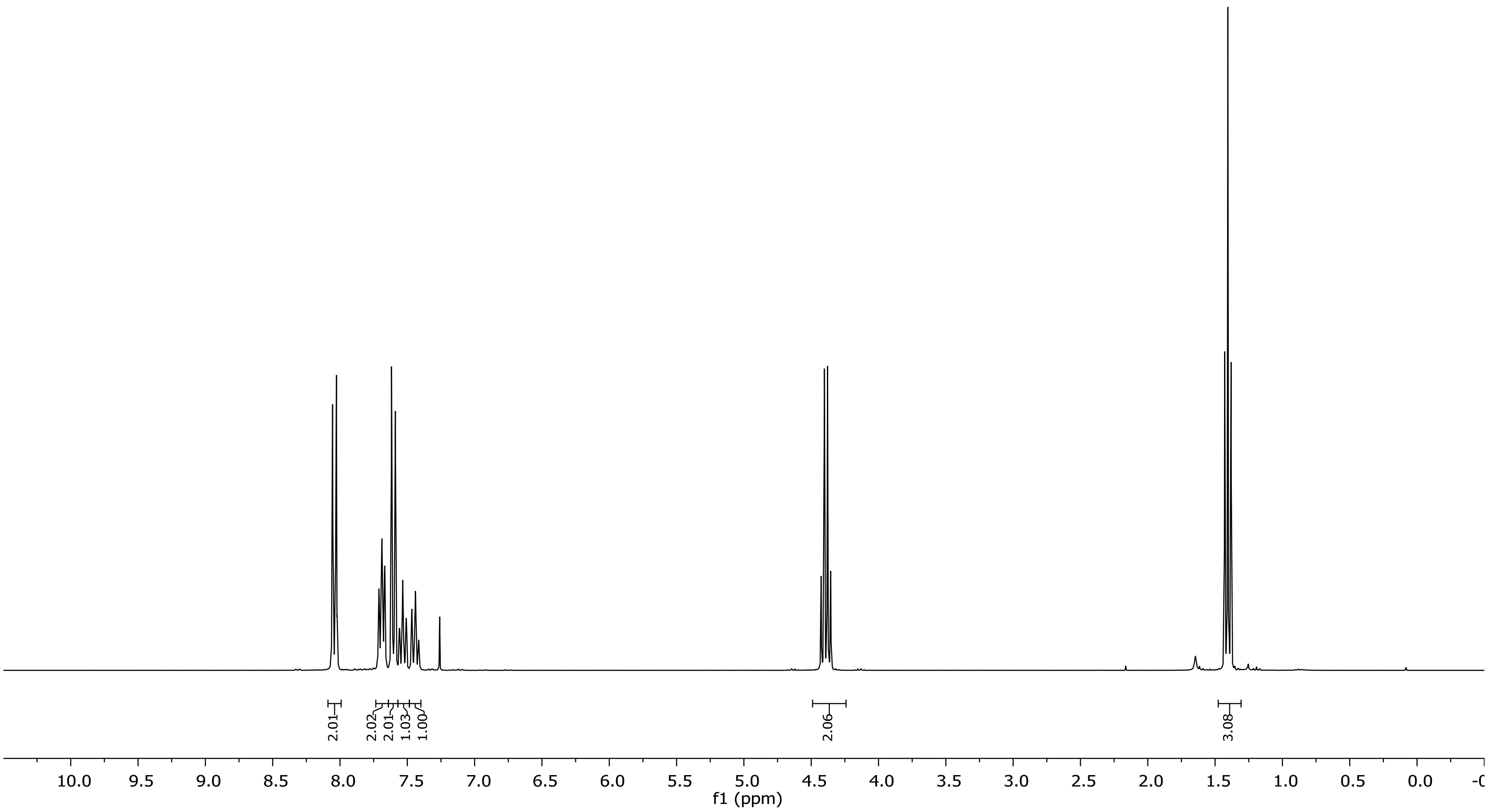
2.01
2.02
2.01
1.03
1.00
2.06
3.08
10.0
9.5
9.0
8.5
8.0
7.5
7.0
6.5
6.0
5.5
5.0
4.5
4.0
3.5
3.0
2.5
2.0
1.5
1.0
0.5
0.0
f1 (ppm)

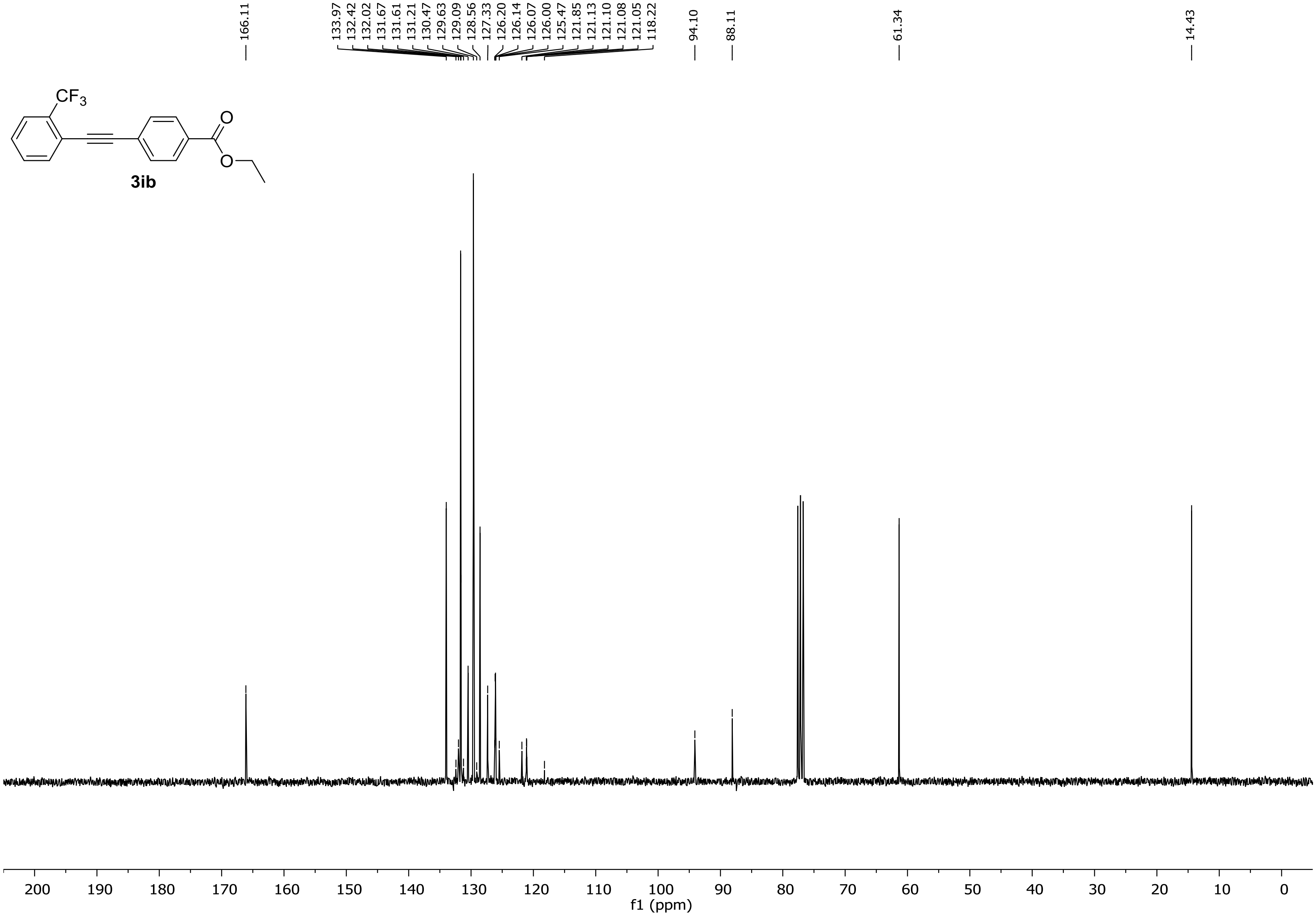

166.11
133.97
132.42
132.02
131.67
131.61
131.21
130.47
129.63
129.09
128.56
127.33
126.20
126.14
126.07
126.00
125.47
121.85
121.13
121.10
121.08
121.05
118.22
94.10
88.11
61.34
14.43
CF3
O
O
3ib
200
190
180
170
160
150
140
130
120
110
100
90
80
70
60
50
40
30
20
10
0
f1 (ppm)

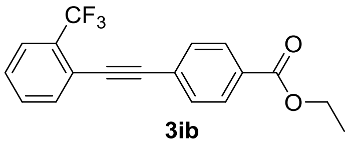

3ib

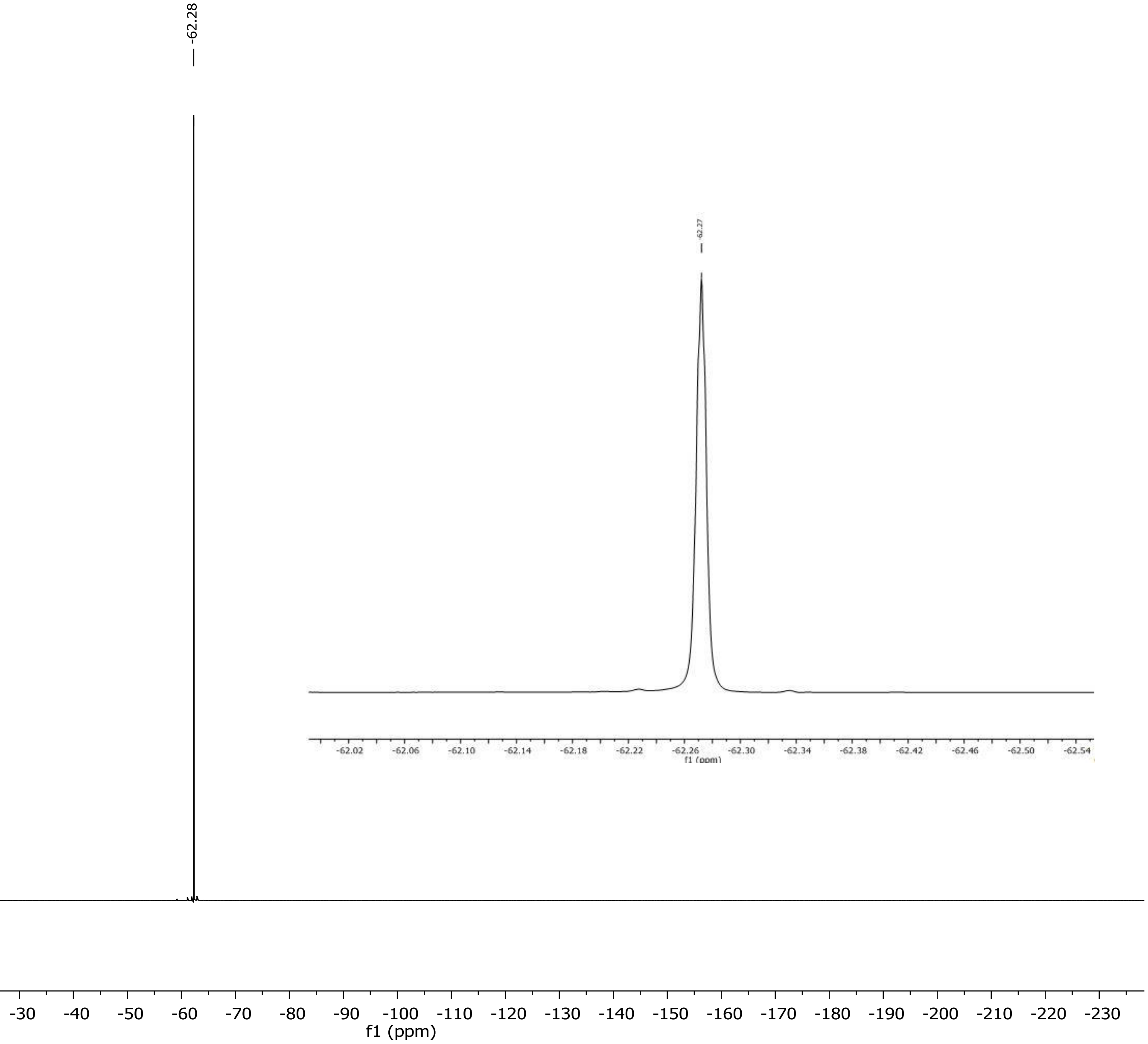

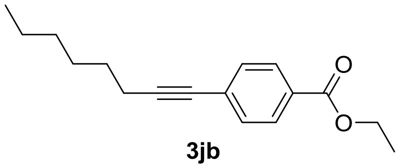
O
O

3jb

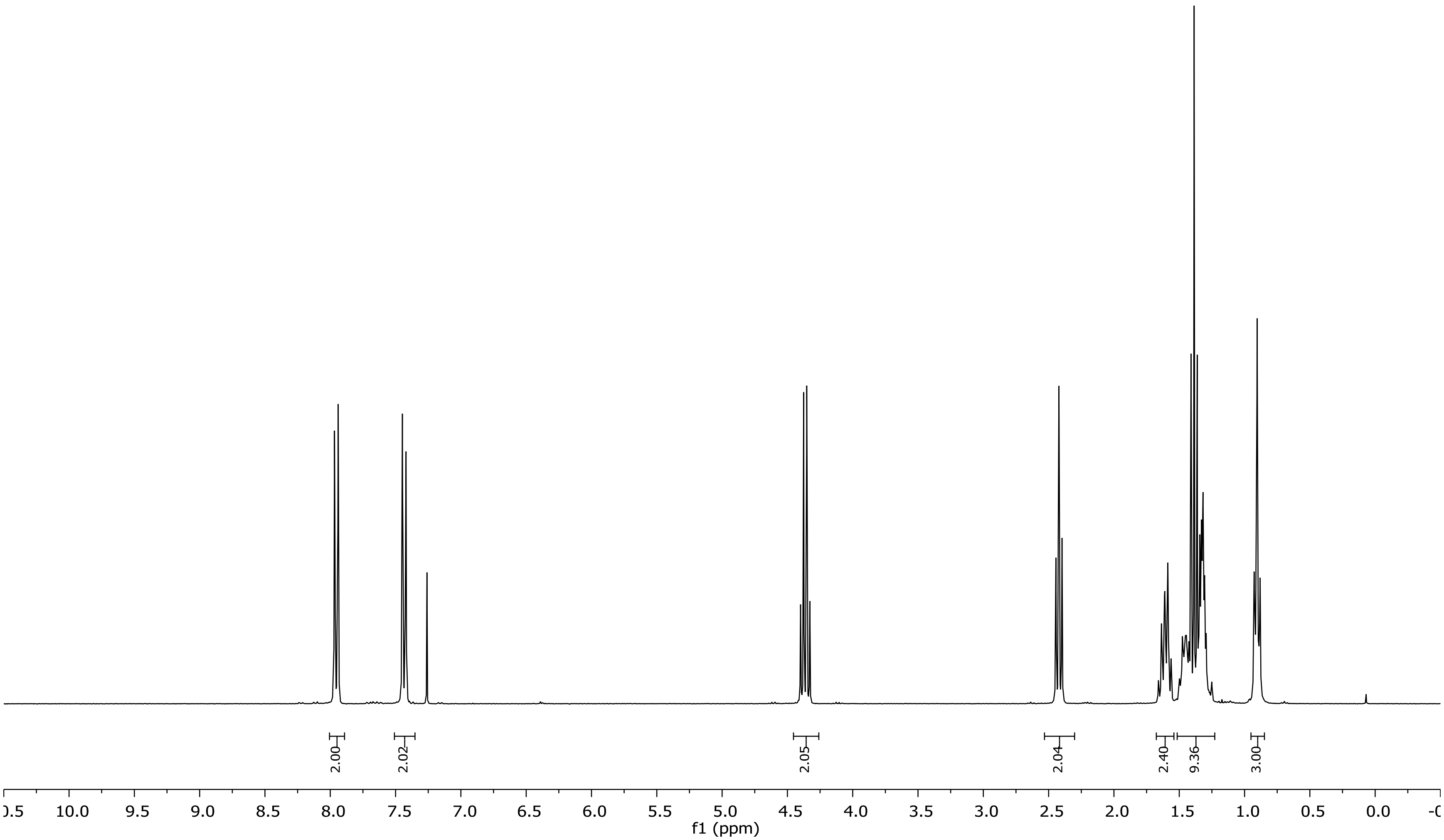
2.00
2.02
2.05
2.04
2.40
9.36
3.00
10.0
9.5
9.0
8.5
8.0
7.5
7.0
6.5
6.0
5.5
5.0
4.5
4.0
3.5
3.0
2.5
2.0
1.5
1.0
0.5
0.0
f1 (ppm)

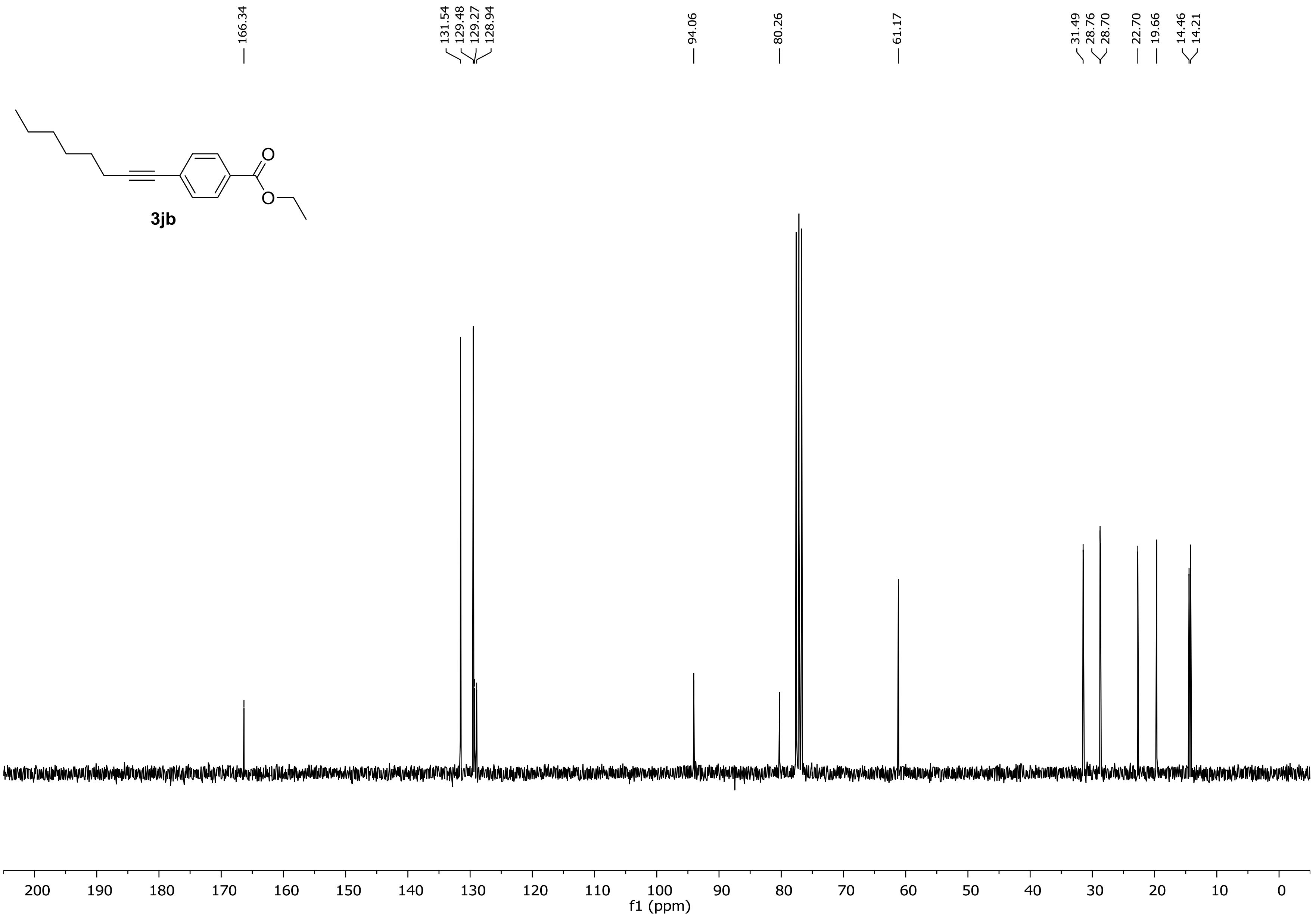
166.34
131.54
129.48
129.27
128.94
94.06
80.26
61.17
31.49
28.76
28.70
22.70
19.66
14.46
14.21
3jb
f1 (ppm)

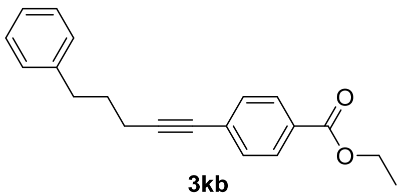

3kb

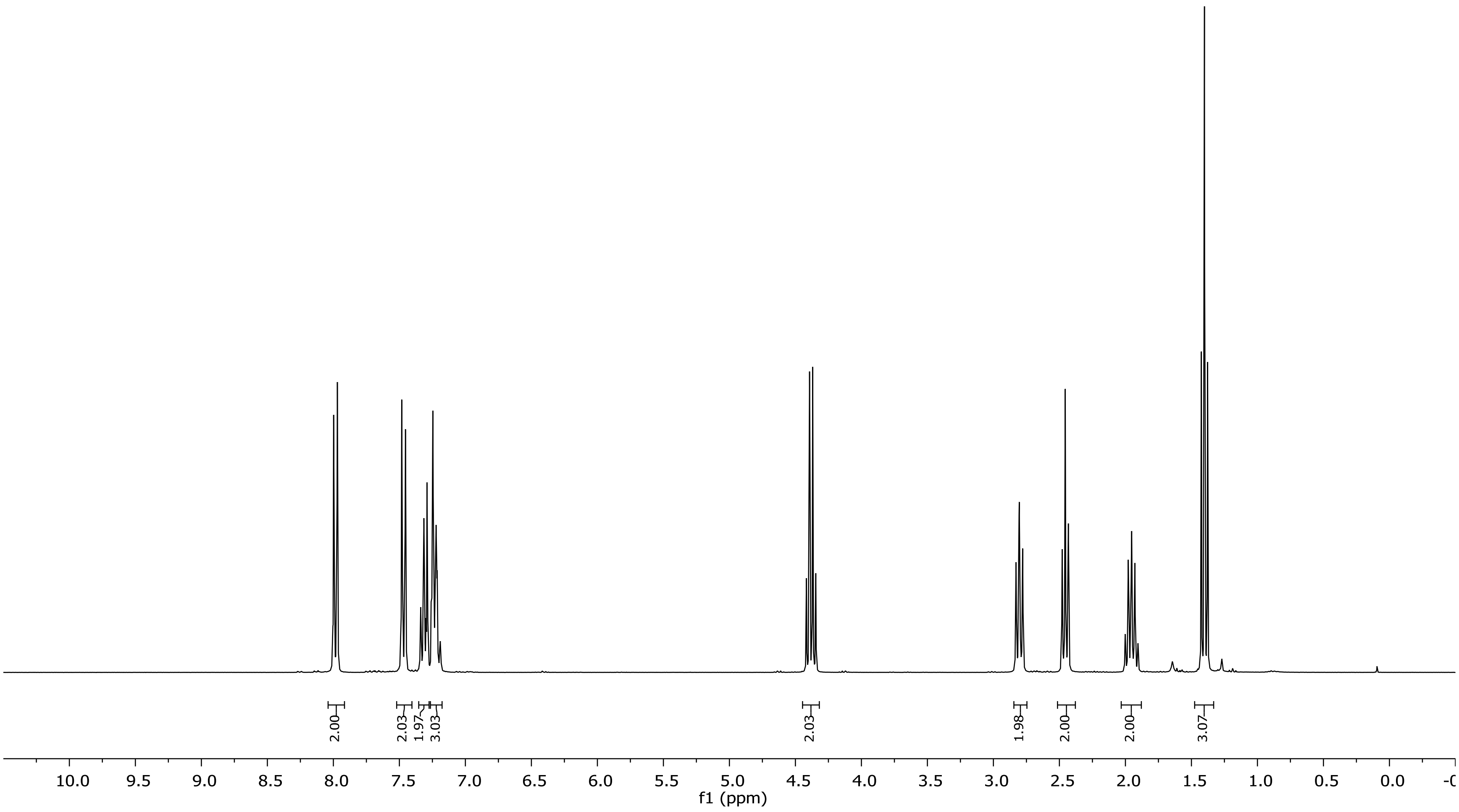

2.00
2.03
1.97
3.03
2.03
1.98
2.00
2.00
3.07
10.0 9.5 9.0 8.5 8.0 7.5 7.0 6.5 6.0 5.5 5.0 4.5 4.0 3.5 3.0 2.5 2.0 1.5 1.0 0.5 0.0
f1 (ppm)

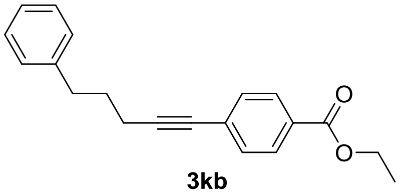

3kb

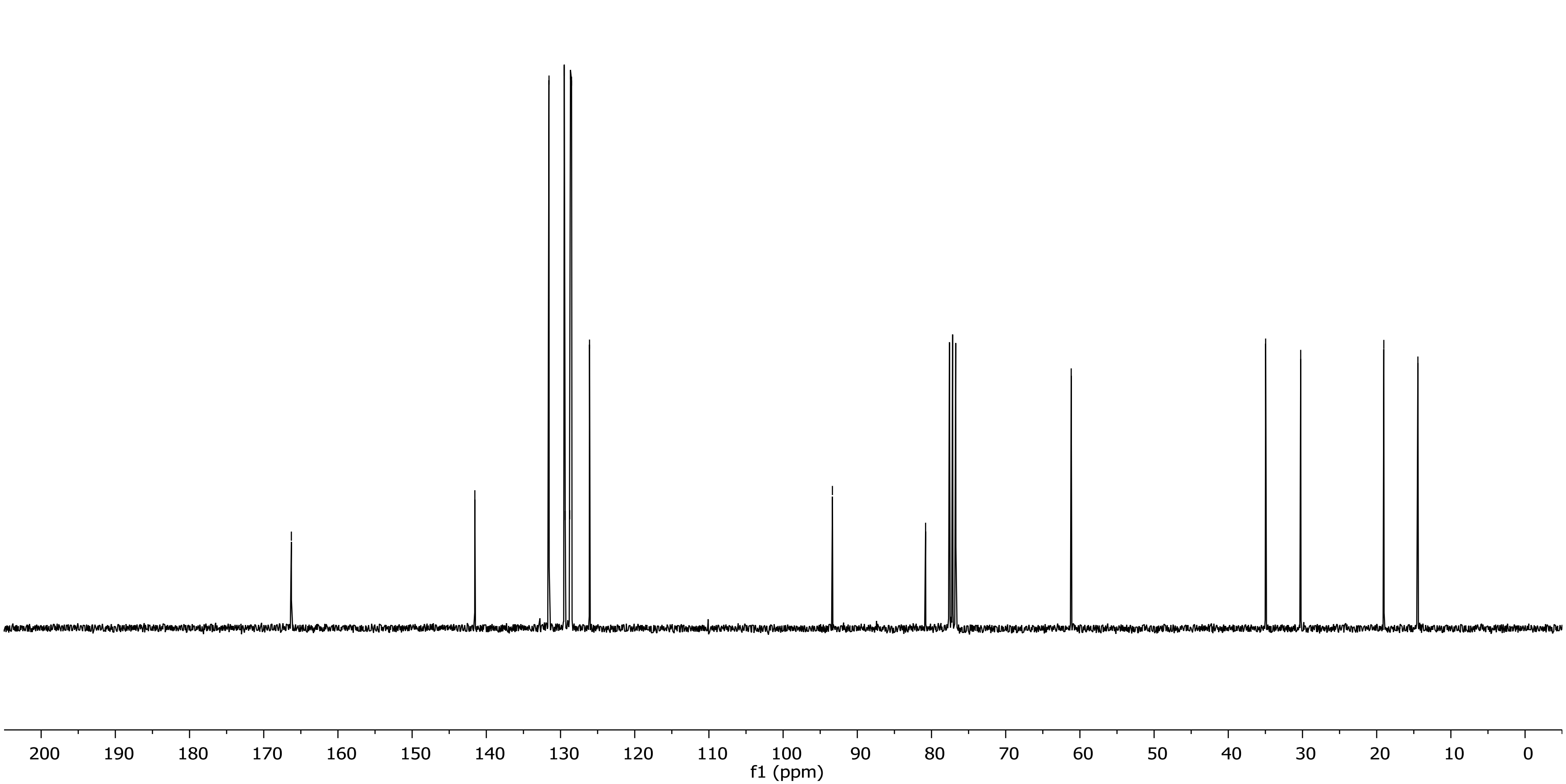

166.28
141.55
131.55
129.49
129.36
128.76
128.65
128.51
126.09
93.35
80.80
61.17
34.96
30.24
19.04
14.45
200 190 180 170 160 150 140 130 120 110 100 90 80 70 60 50 40 30 20 10 0
f1 (ppm)

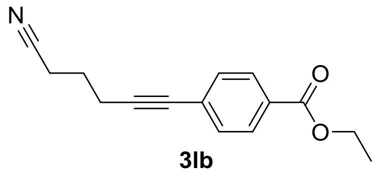

3lb

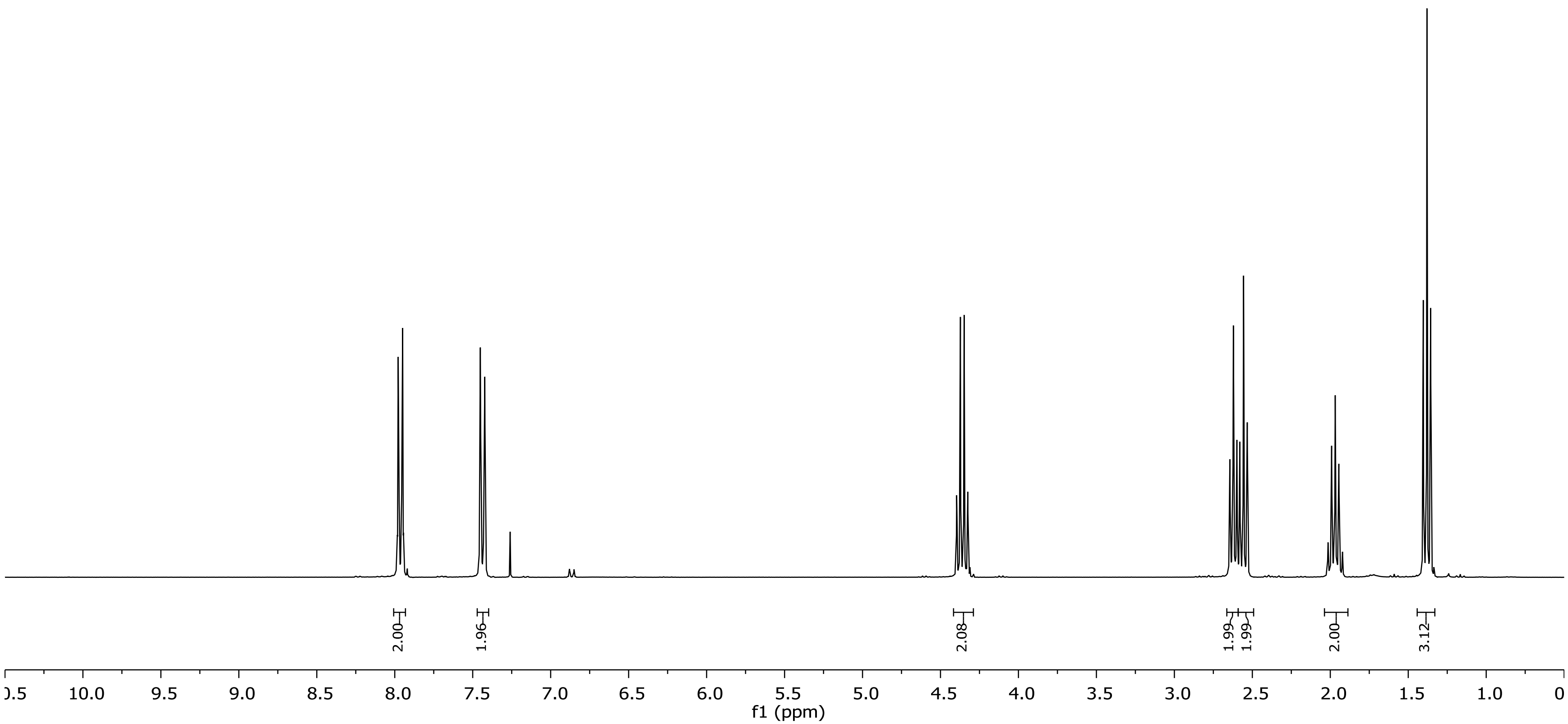

2.00
1.96
2.08
1.99
1.99
2.00
3.12
10.0
9.5
9.0
8.5
8.0
7.5
7.0
6.5
6.0
5.5
5.0
4.5
4.0
3.5
3.0
2.5
2.0
1.5
1.0
f1 (ppm)

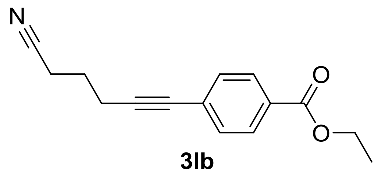

3lb

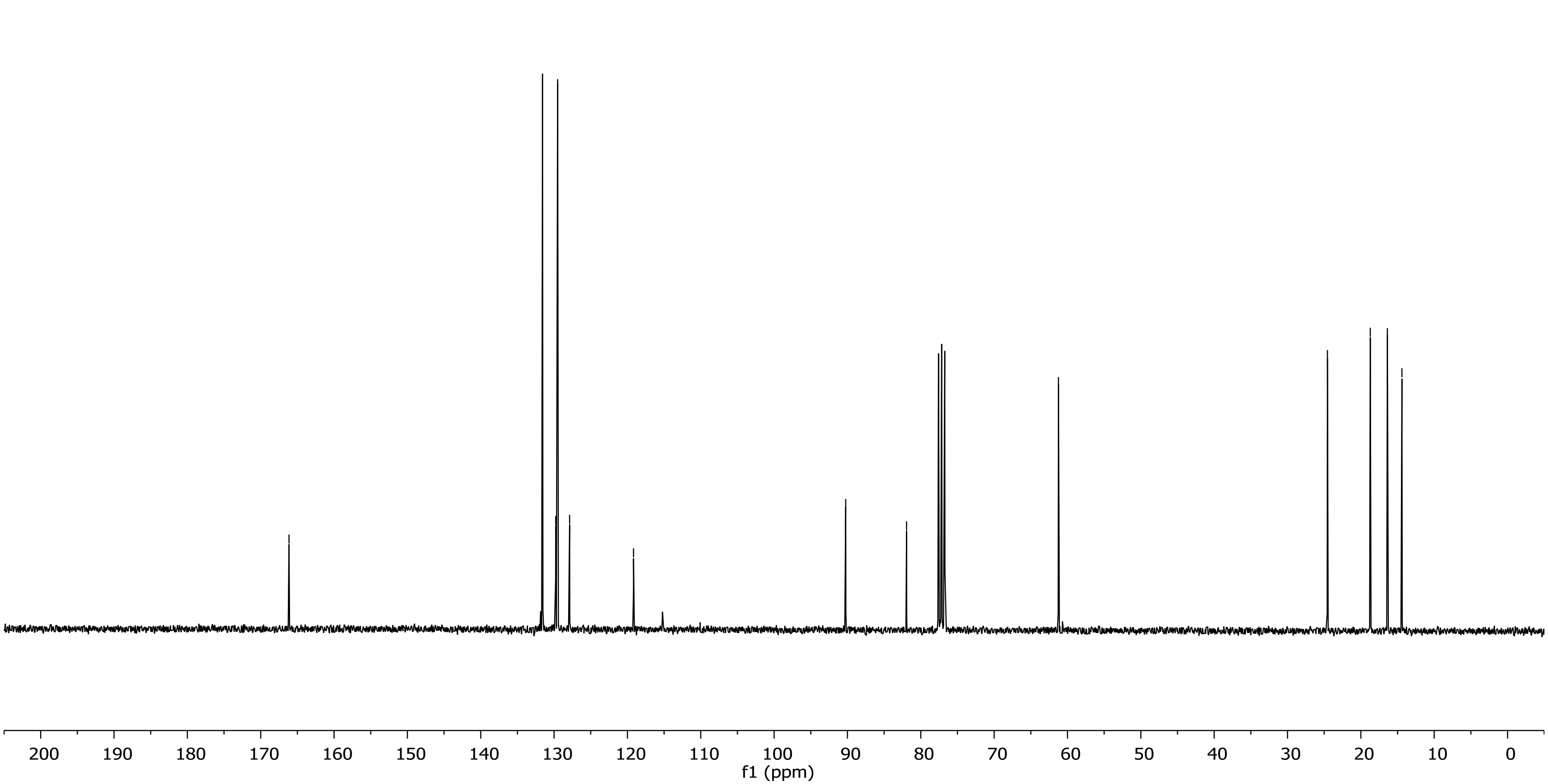
166.14
131.57
129.78
129.51
127.89
119.16
90.24
81.94
61.23
24.56
18.72
16.38
14.40
f1 (ppm)

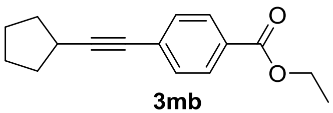

3mb

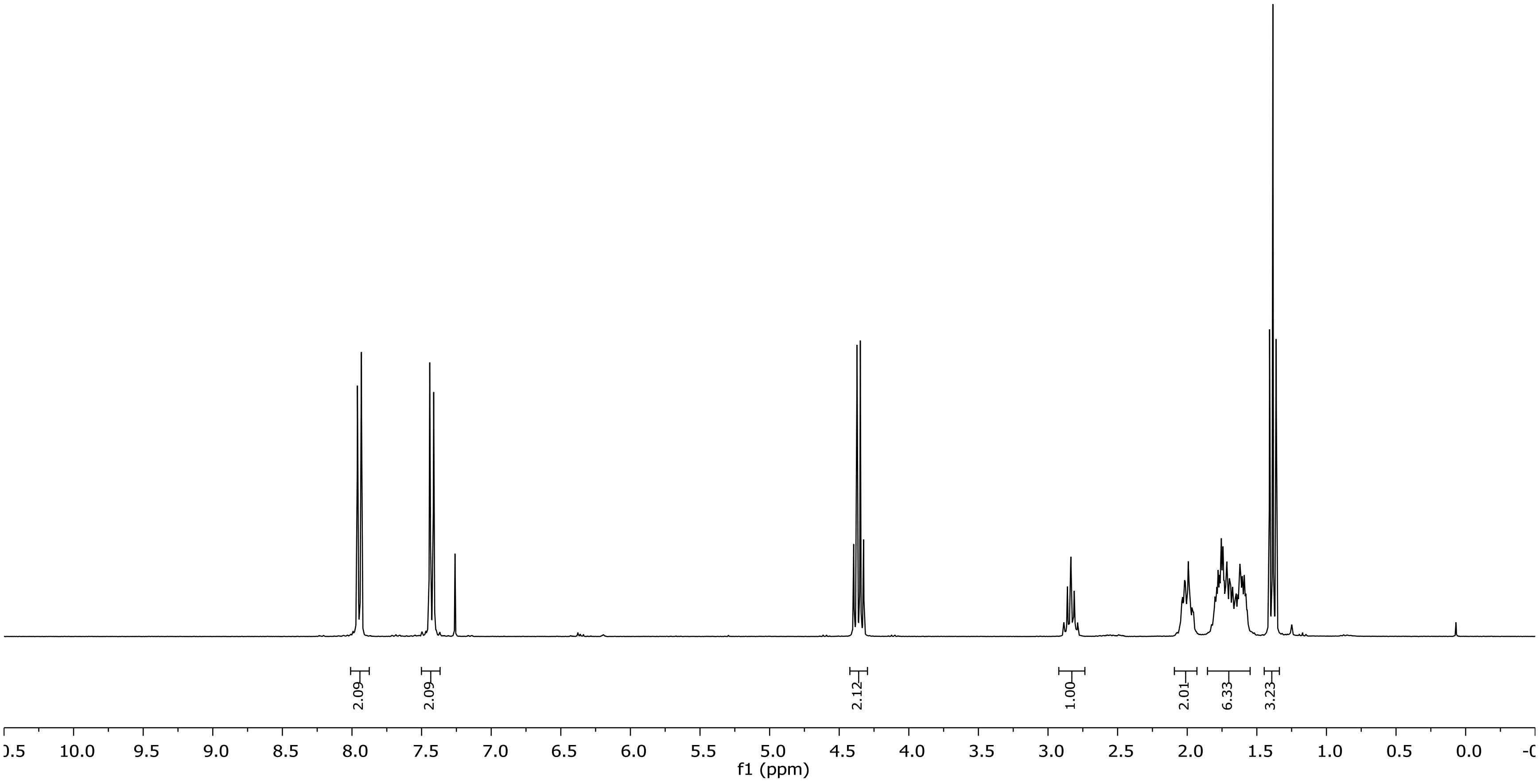
2.09
2.09
2.12
1.00
2.01
6.33
3.23
0.5 10.0 9.5 9.0 8.5 8.0 7.5 7.0 6.5 6.0 5.5 5.0 4.5 4.0 3.5 3.0 2.5 2.0 1.5 1.0 0.5 0.0
f1 (ppm)

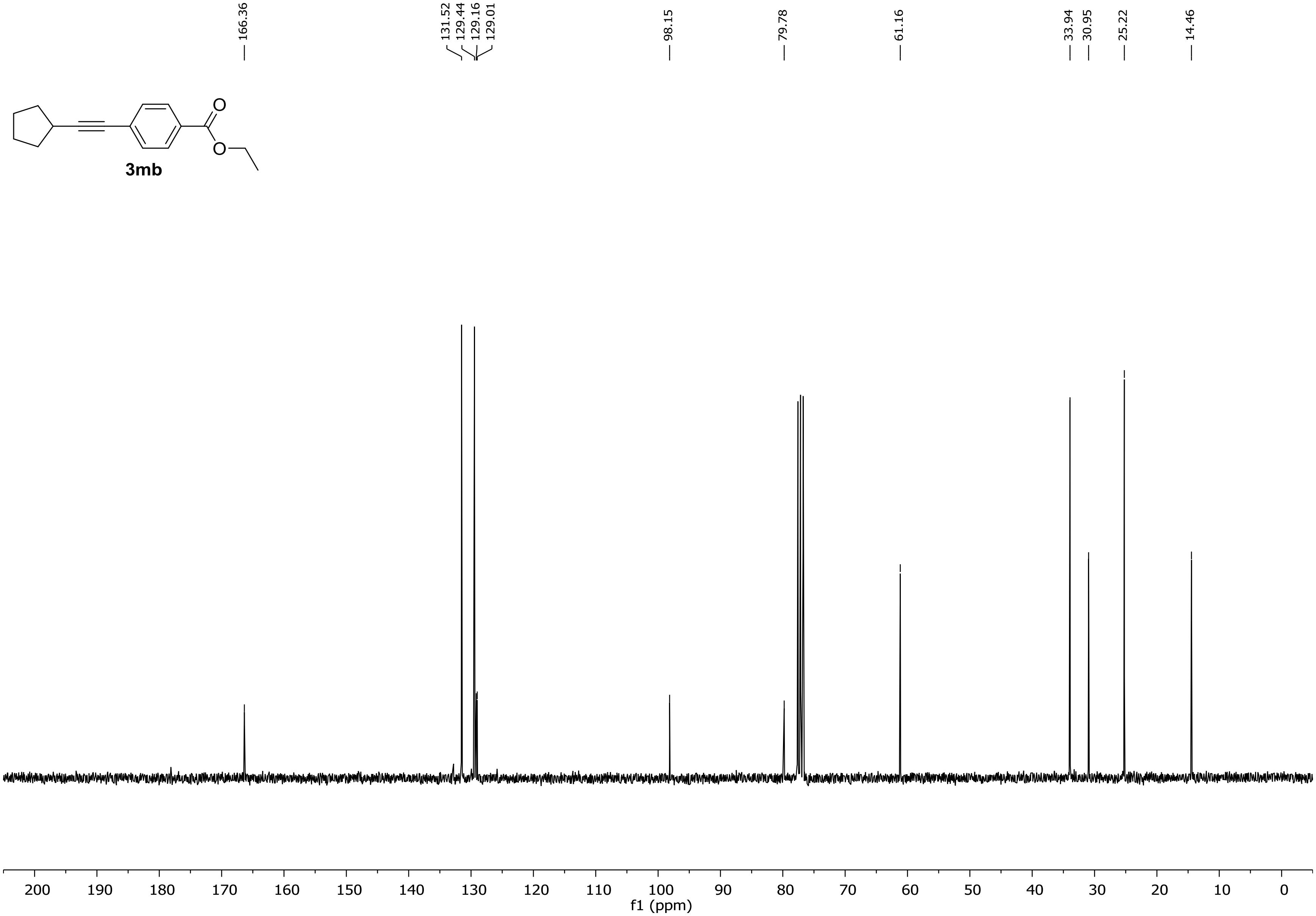
166.36
131.52
129.44
129.16
129.01
98.15
79.78
61.16
33.94
30.95
25.22
14.46
3mb
200 190 180 170 160 150 140 130 120 110 100 90 80 70 60 50 40 30 20 10 0
f1 (ppm)

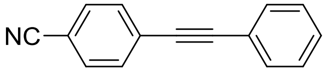
NC

3fa

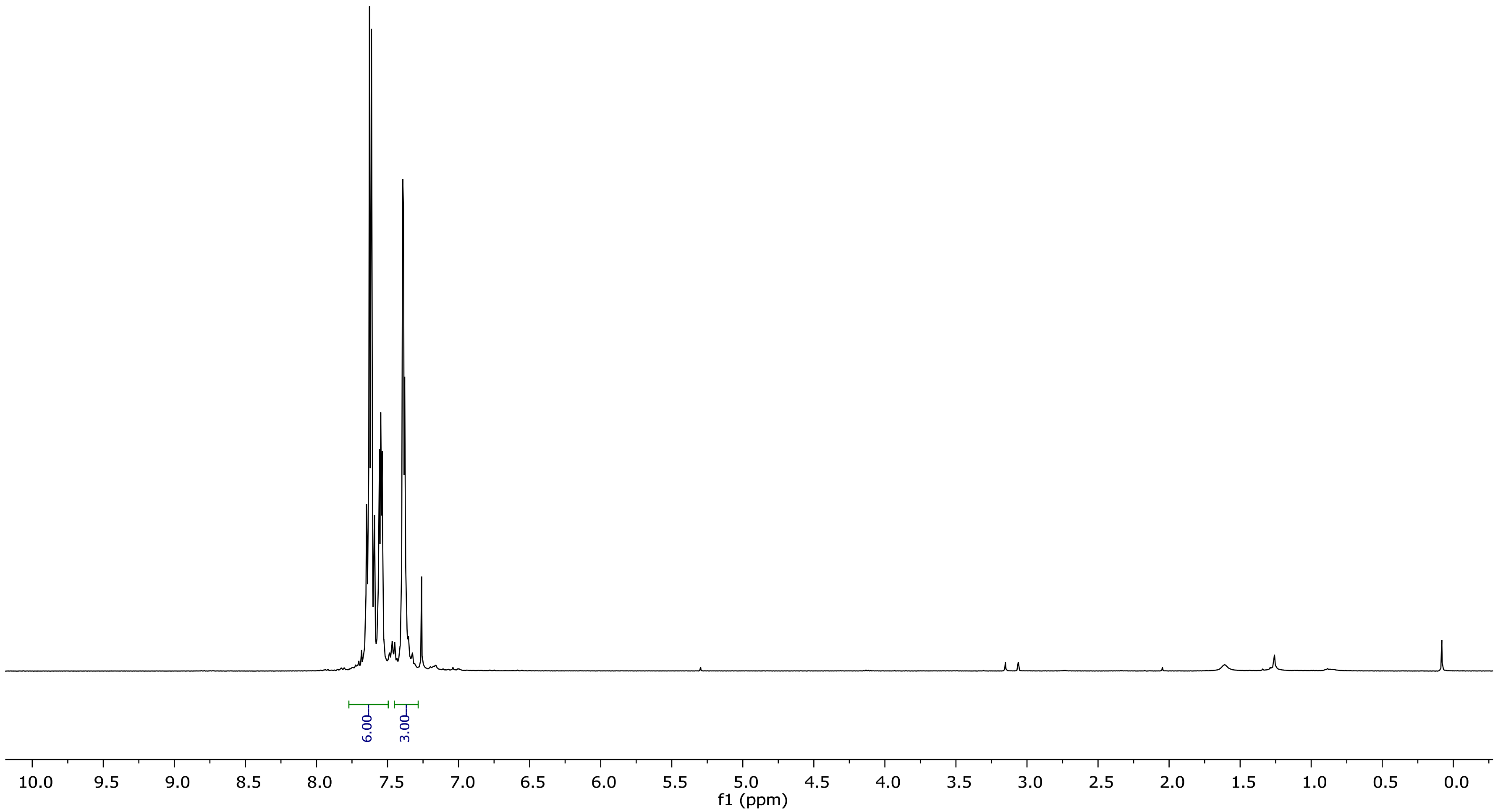
6.00
3.00
10.0 9.5 9.0 8.5 8.0 7.5 7.0 6.5 6.0 5.5 5.0 4.5 4.0 3.5 3.0 2.5 2.0 1.5 1.0 0.5 0.0
f1 (ppm)

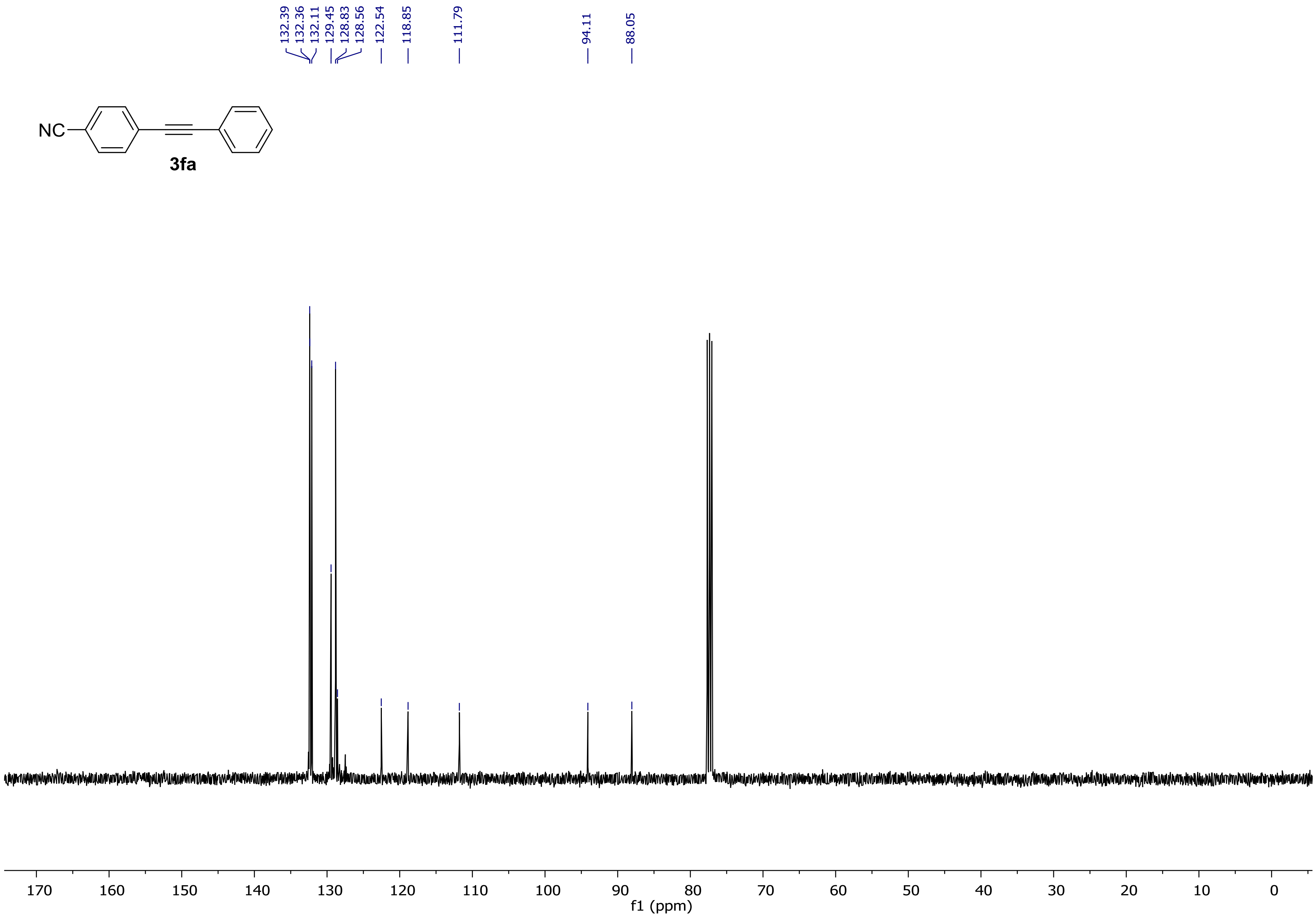
132.39
132.36
132.11
129.45
128.83
128.56
122.54
118.85
111.79
94.11
88.05
NC
3fa
170
160
150
140
130
120
110
100
90
80
70
60
50
40
30
20
10
0
f1 (ppm)

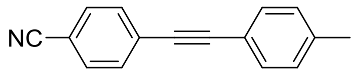
NC

3fc

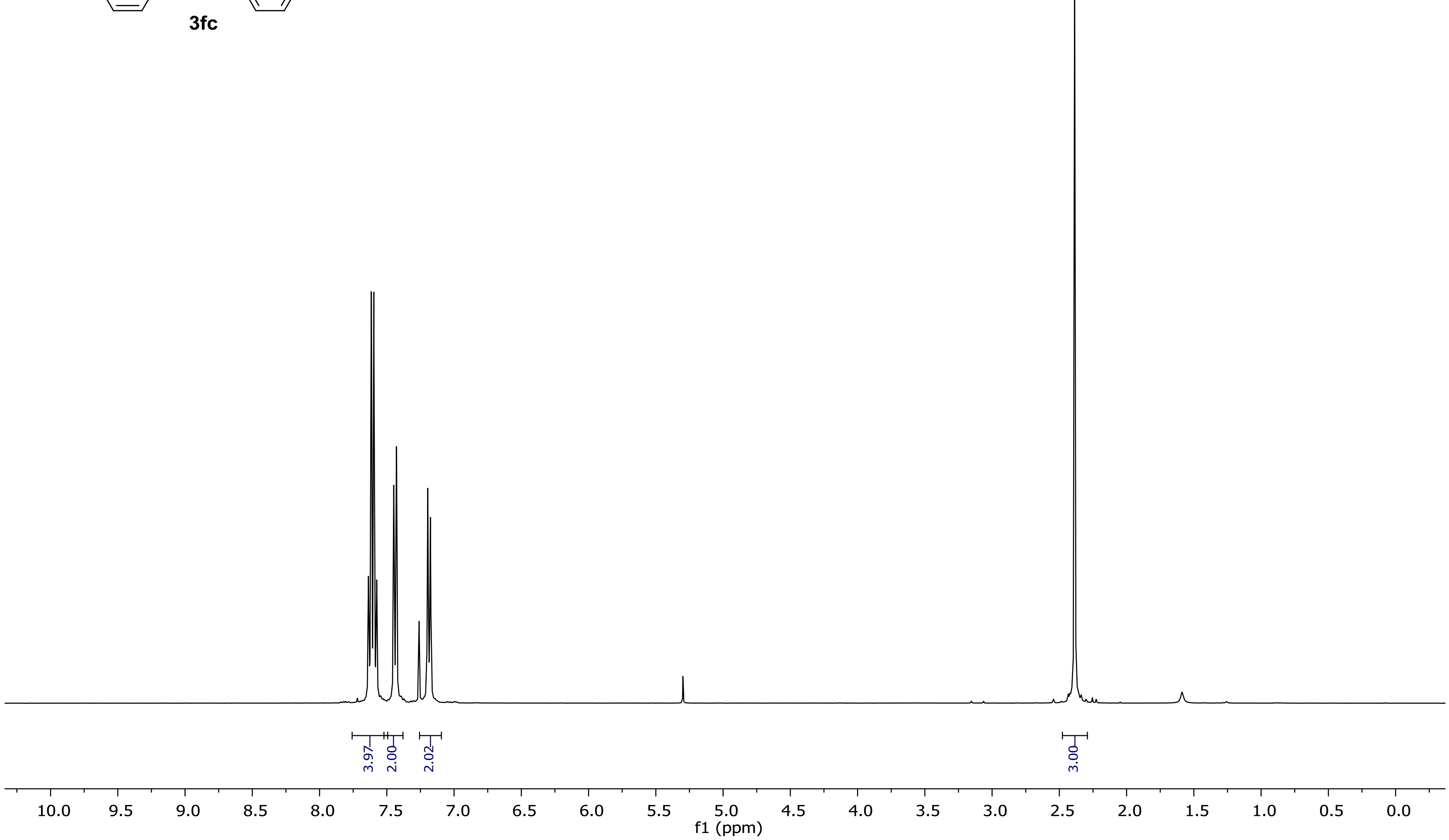
3.97
2.00
2.02
3.00
10.0
9.5
9.0
8.5
8.0
7.5
7.0
6.5
6.0
5.5
5.0
4.5
4.0
3.5
3.0
2.5
2.0
1.5
1.0
0.5
0.0
f1 (ppm)

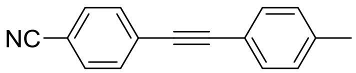

3fc

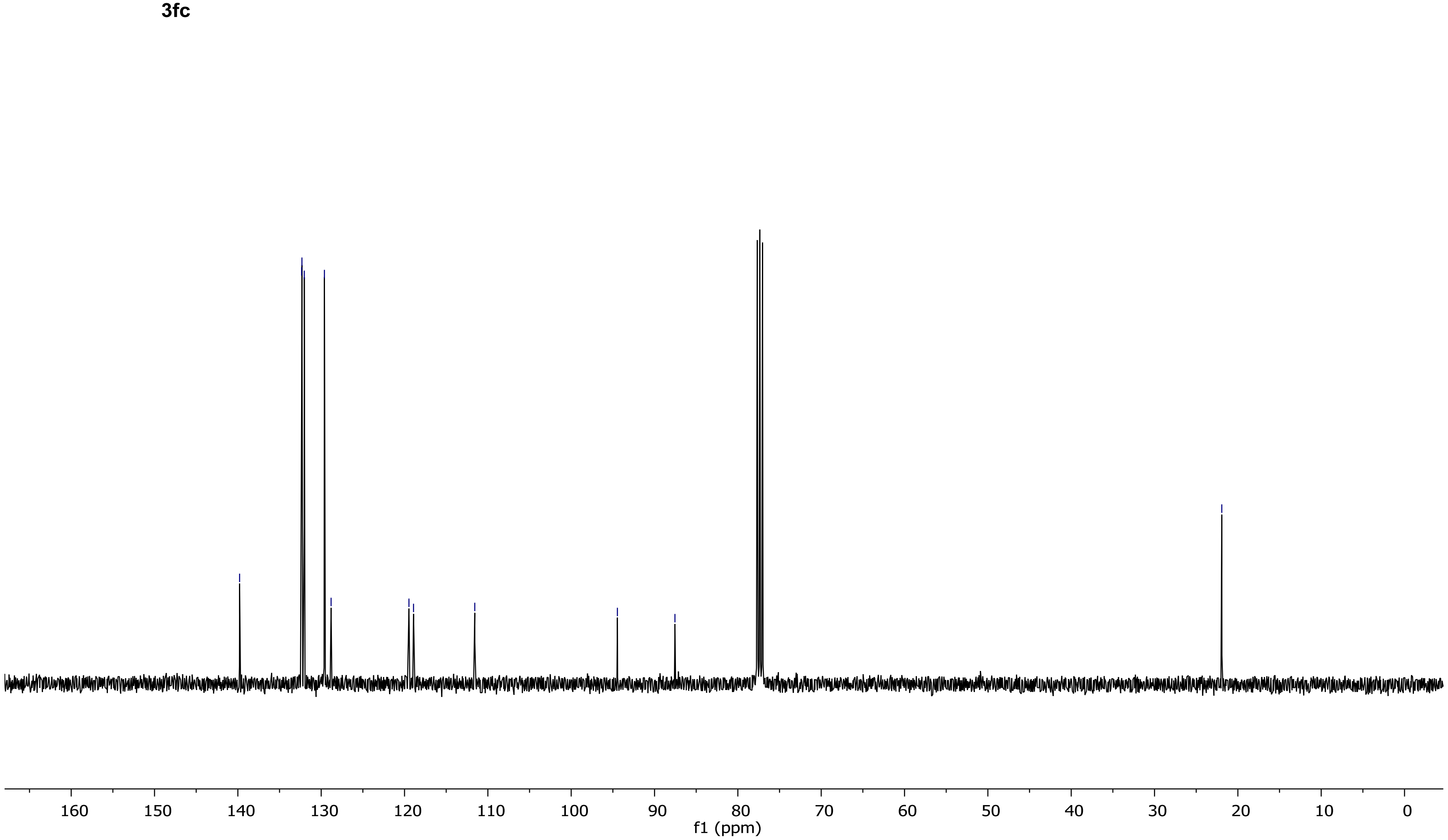
139.79
132.35
132.31
132.03
129.61
128.81
119.47
118.92
111.57
94.46
87.55
21.93
160
150
140
130
120
110
100
90
80
70
60
50
40
30
20
10
0
f1 (ppm)

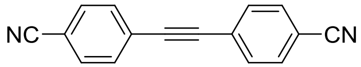
NC
CN

3fd

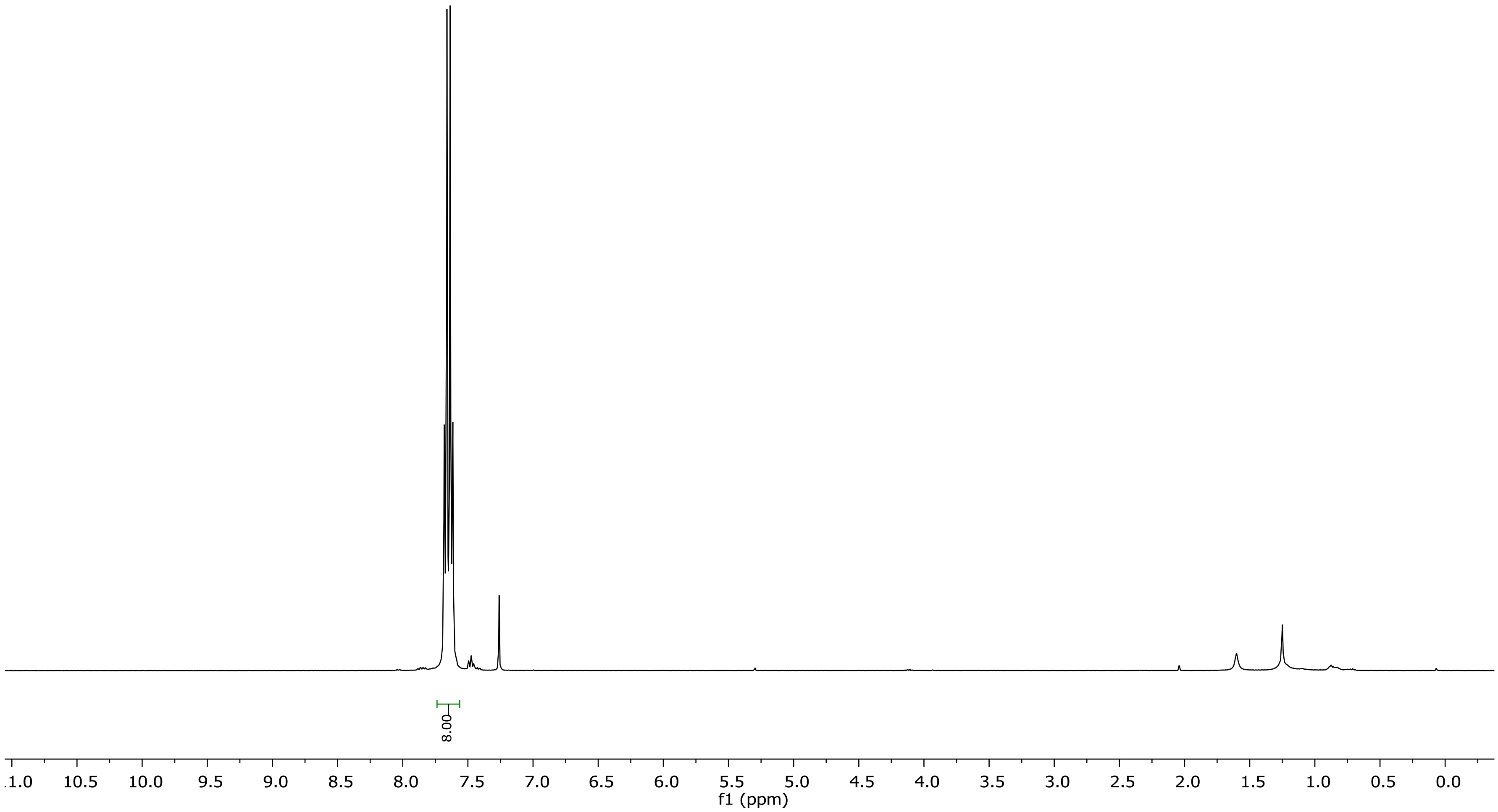
8.00
.1.0
10.5
10.0
9.5
9.0
8.5
8.0
7.5
7.0
6.5
6.0
5.5
5.0
4.5
4.0
3.5
3.0
2.5
2.0
1.5
1.0
0.5
0.0
f1 (ppm)

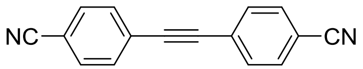

3fd

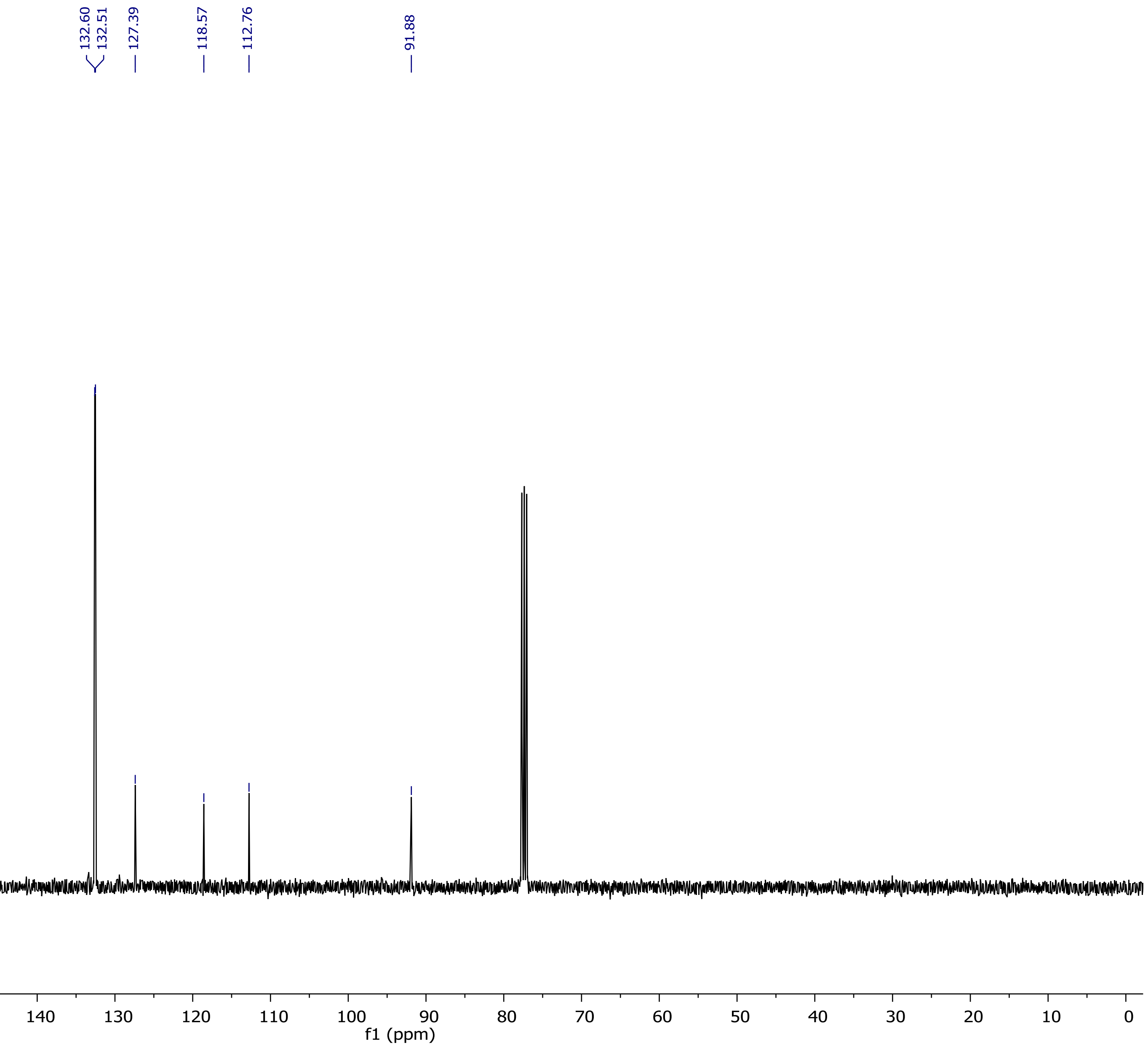
132.60
132.51
127.39
118.57
112.76
91.88
180
170
160
150
140
130
120
110
100
90
80
70
60
50
40
30
20
10
0
f1 (ppm)

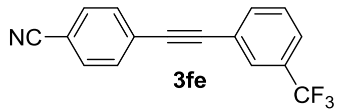
NC
CF3

3fe

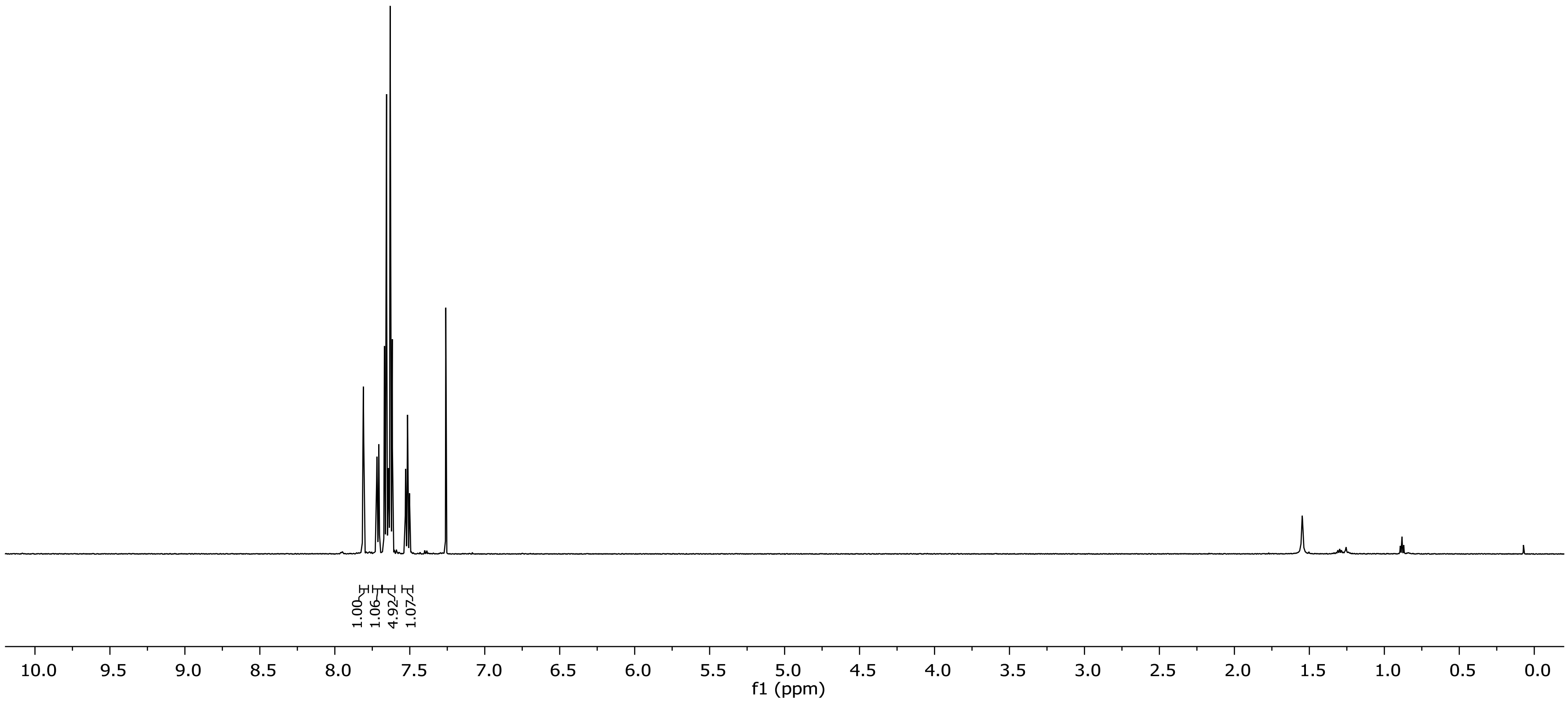
1.00
1.06
4.92
1.07
10.0
9.5
9.0
8.5
8.0
7.5
7.0
6.5
6.0
5.5
5.0
4.5
4.0
3.5
3.0
2.5
2.0
1.5
1.0
0.5
0.0
f1 (ppm)

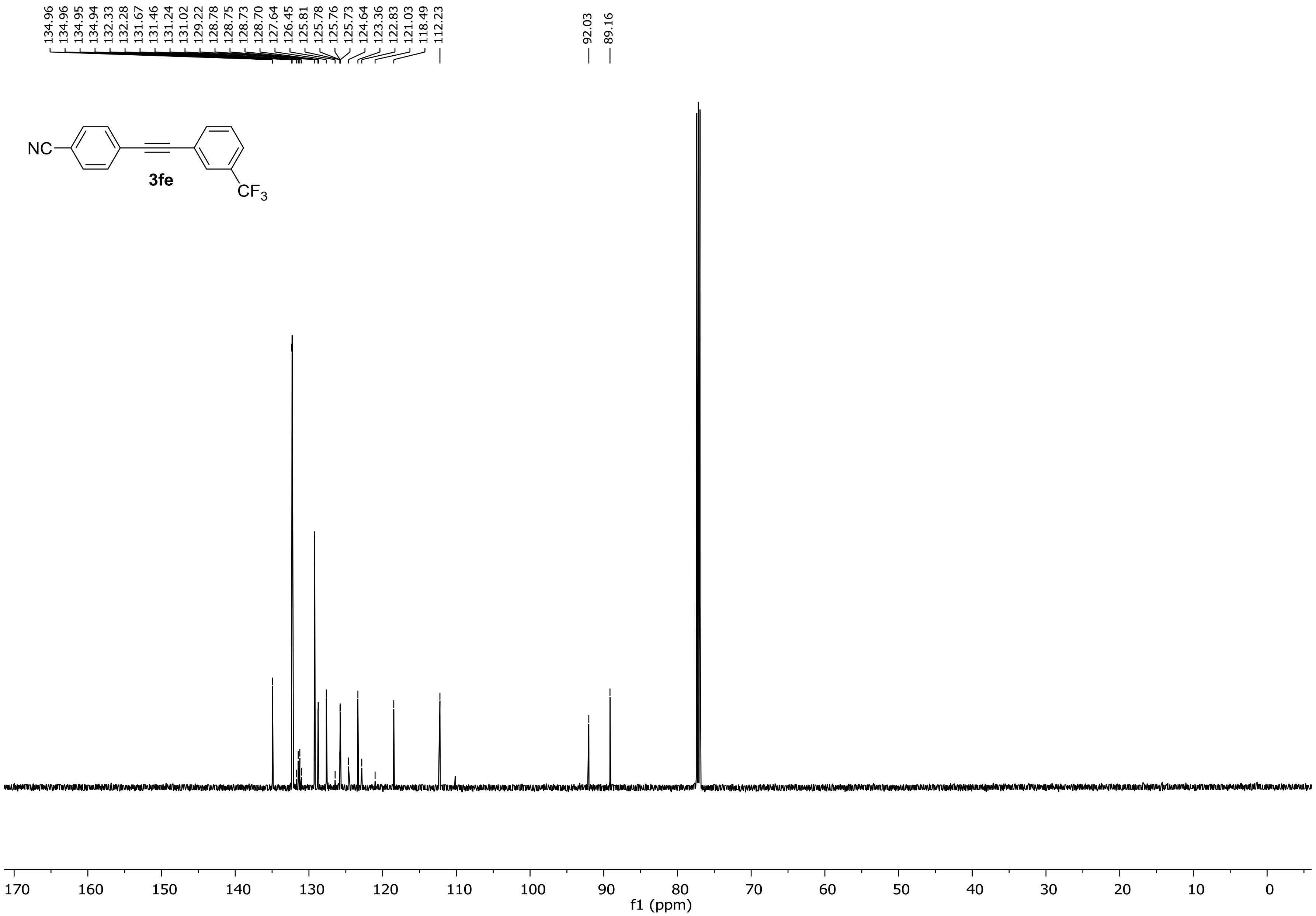
134.96
134.96
134.95
134.94
132.33
132.28
131.67
131.46
131.24
131.02
129.22
128.78
128.75
128.73
128.70
127.64
126.45
125.81
125.78
125.76
125.73
124.64
123.36
122.83
121.03
118.49
112.23
92.03
89.16
NC
3fe
CF_3
170 160 150 140 130 120 110 100 90 80 70 60 50 40 30 20 10 0
f1 (ppm)

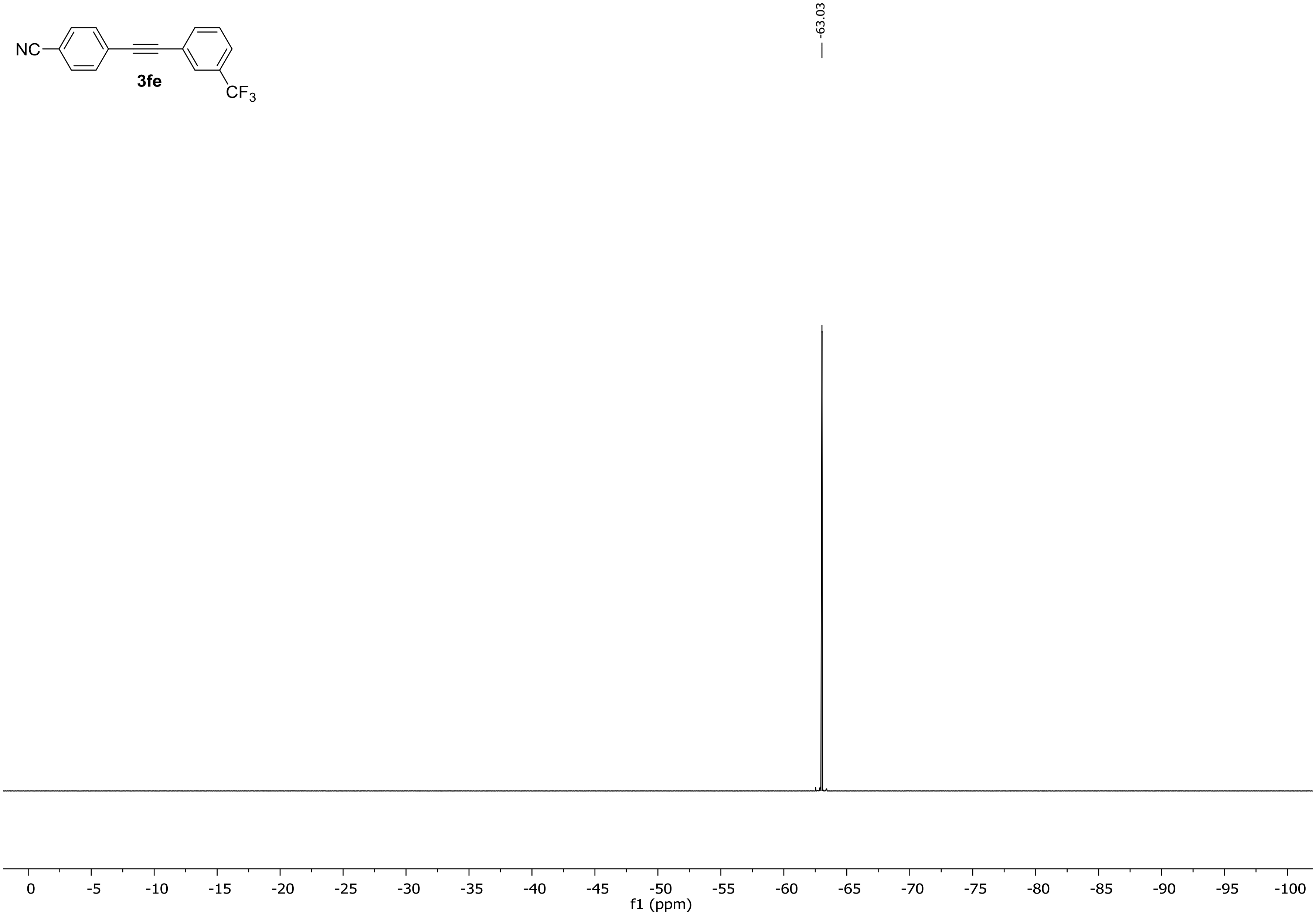
NC
3fe
CF3
-63.03
0 -5 -10 -15 -20 -25 -30 -35 -40 -45 -50 -55 -60 -65 -70 -75 -80 -85 -90 -95 -100
f1 (ppm)

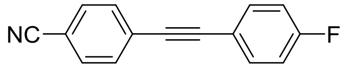
NC
F

3ff

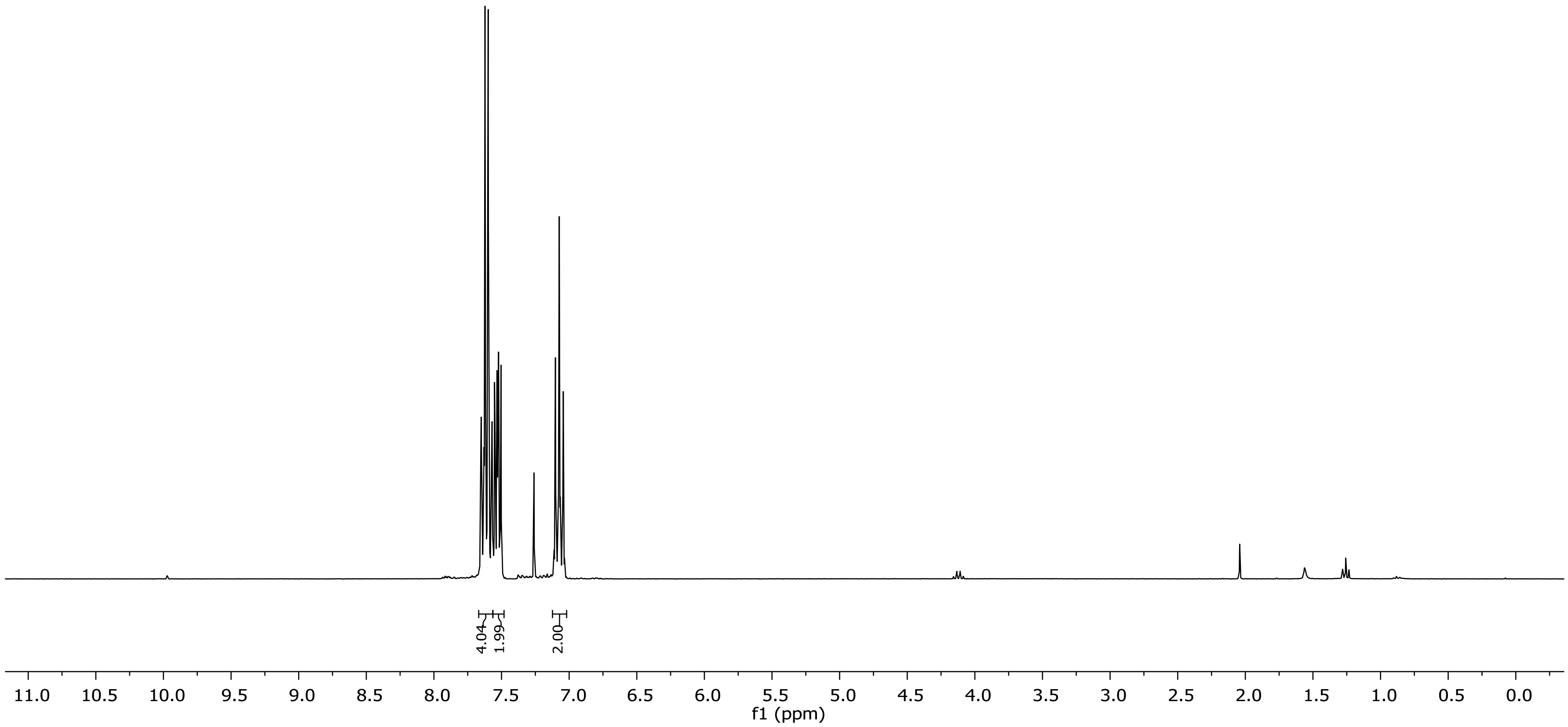
4.04
1.99
2.00
11.0 10.5 10.0 9.5 9.0 8.5 8.0 7.5 7.0 6.5 6.0 5.5 5.0 4.5 4.0 3.5 3.0 2.5 2.0 1.5 1.0 0.5 0.0
f1 (ppm)

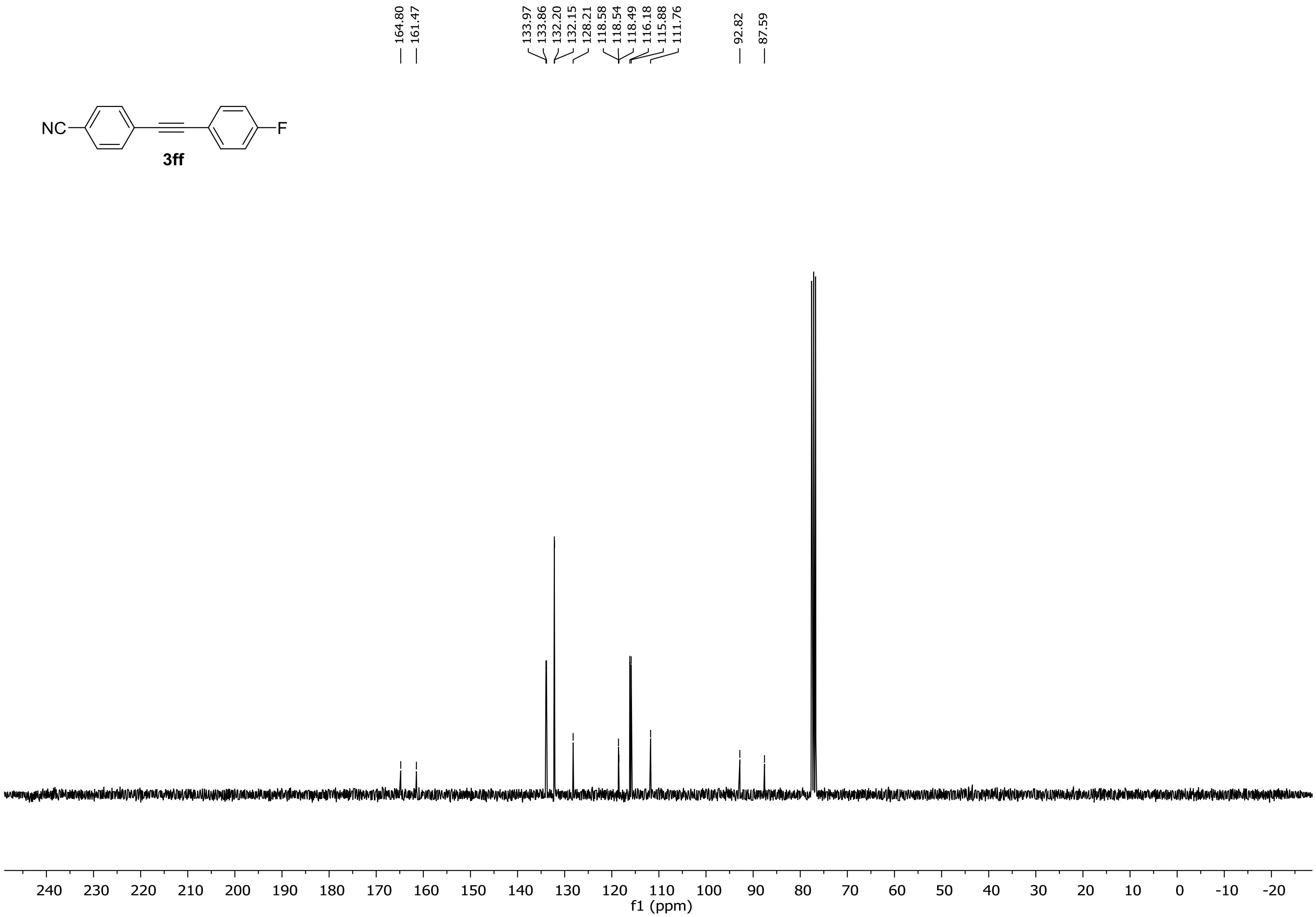
NC
F
164.80
161.47
133.97
133.86
132.20
132.15
128.21
118.58
118.54
118.49
116.18
115.88
111.76
92.82
87.59
240 230 220 210 200 190 180 170 160 150 140 130 120 110 100 90 80 70 60 50 40 30 20 10 0 -10 -20
f1 (ppm)

3ff

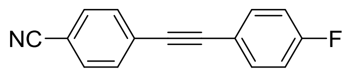
NC
F

3ff

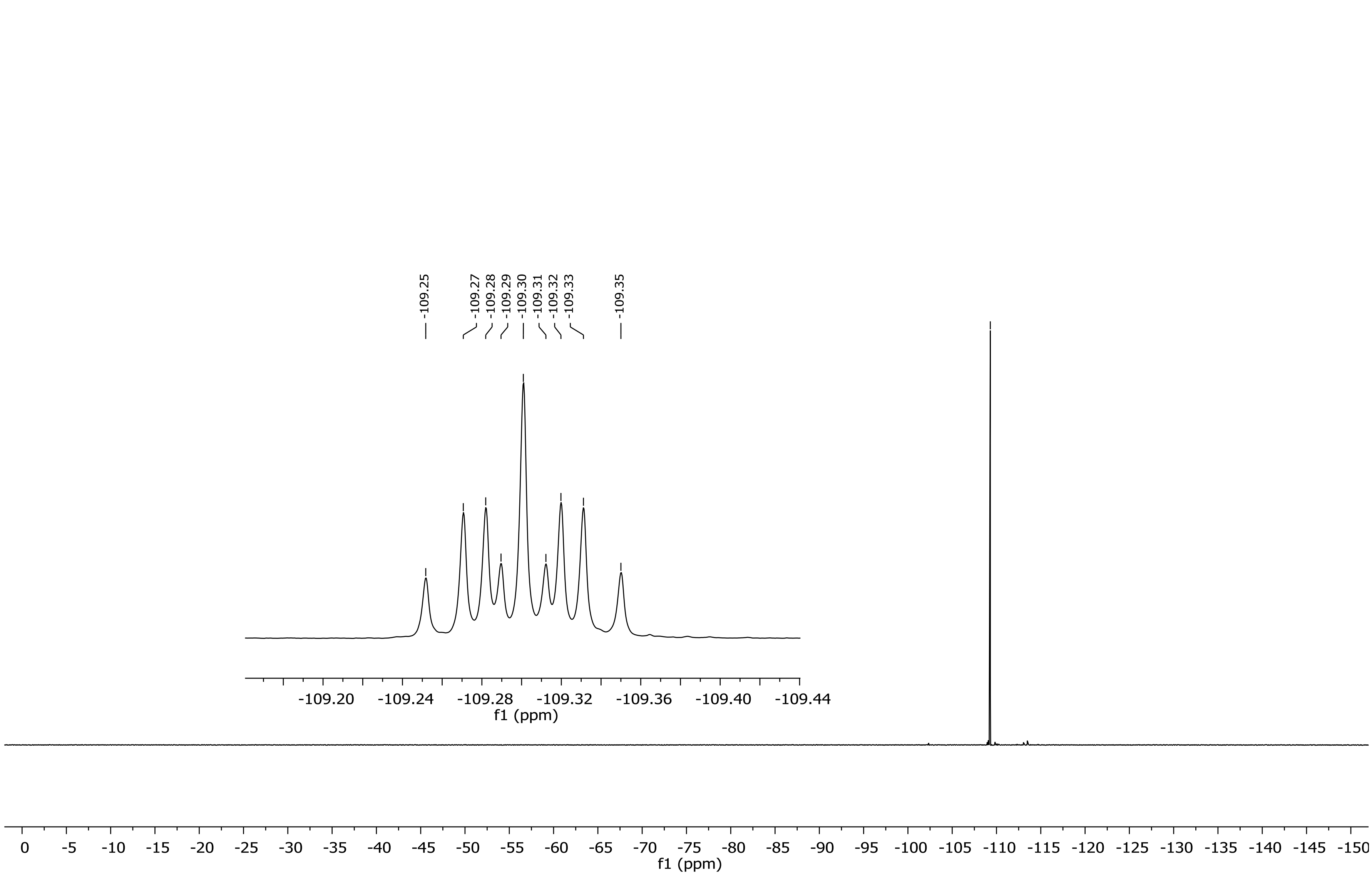
-109.30
-109.25
-109.27
-109.28
-109.29
-109.30
-109.31
-109.32
-109.33
-109.35
-109.20 -109.24 -109.28 -109.32 -109.36 -109.40 -109.44
f1 (ppm)
0 -5 -10 -15 -20 -25 -30 -35 -40 -45 -50 -55 -60 -65 -70 -75 -80 -85 -90 -95 -100 -105 -110 -115 -120 -125 -130 -135 -140 -145 -150
f1 (ppm)

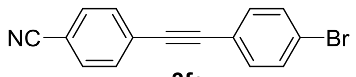
NC
Br

3fg

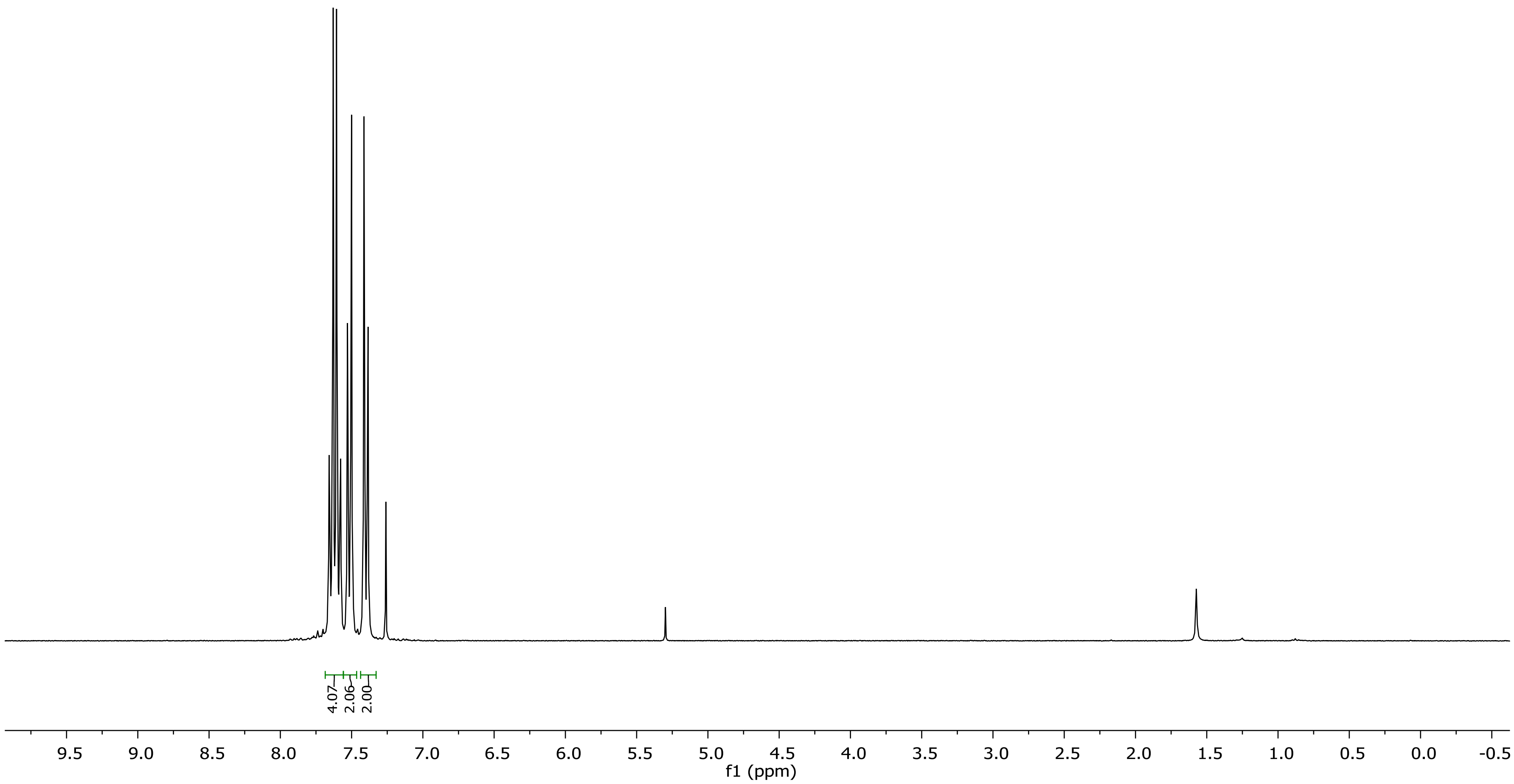
4.07
2.06
2.00
9.5
9.0
8.5
8.0
7.5
7.0
6.5
6.0
5.5
5.0
4.5
4.0
3.5
3.0
2.5
2.0
1.5
1.0
0.5
0.0
-0.5
f1 (ppm)

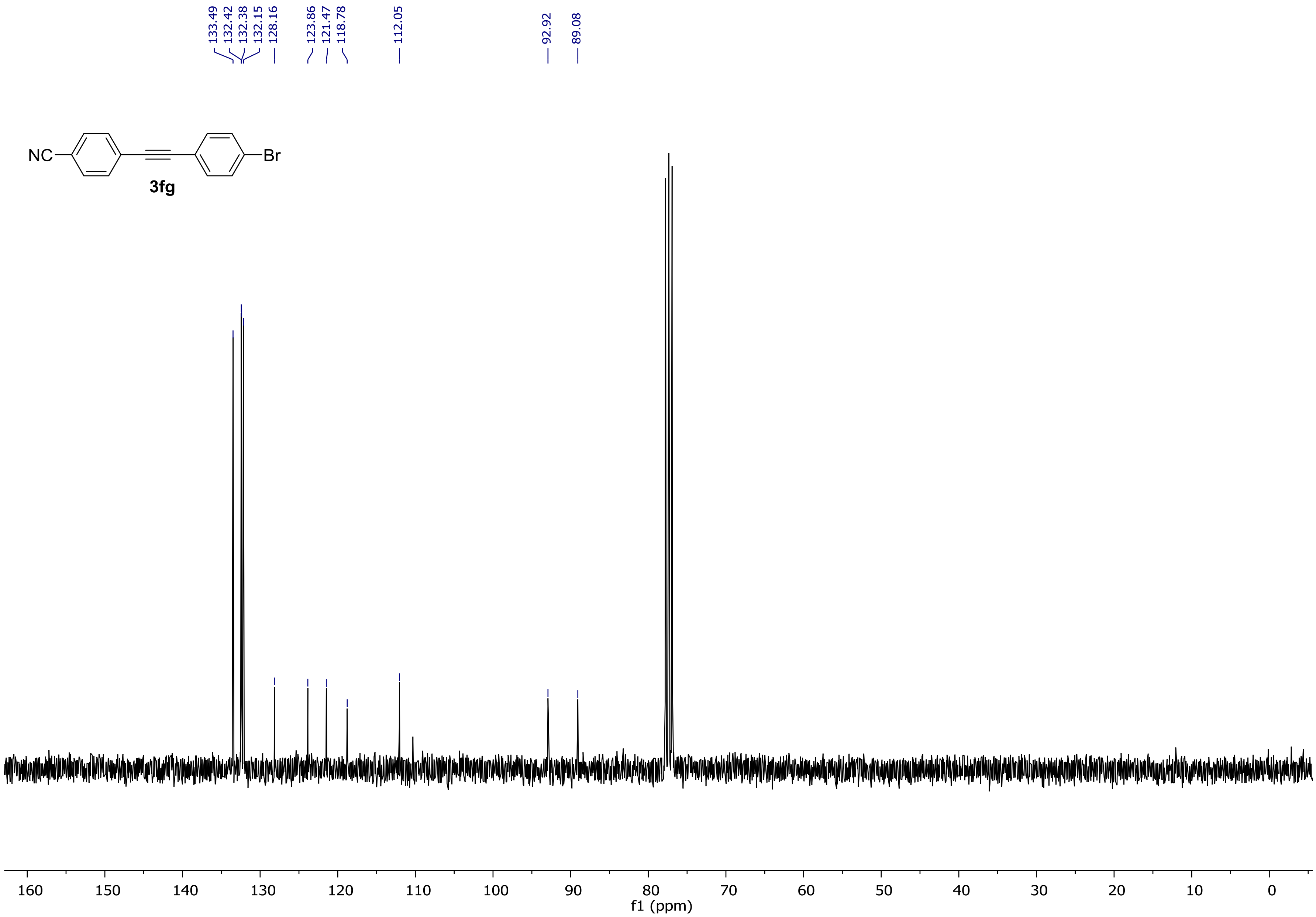
133.49
132.42
132.38
132.15
128.16
123.86
121.47
118.78
112.05
92.92
89.08
NC
Br
3fg
160
150
140
130
120
110
100
90
80
70
60
50
40
30
20
10
0
f1 (ppm)

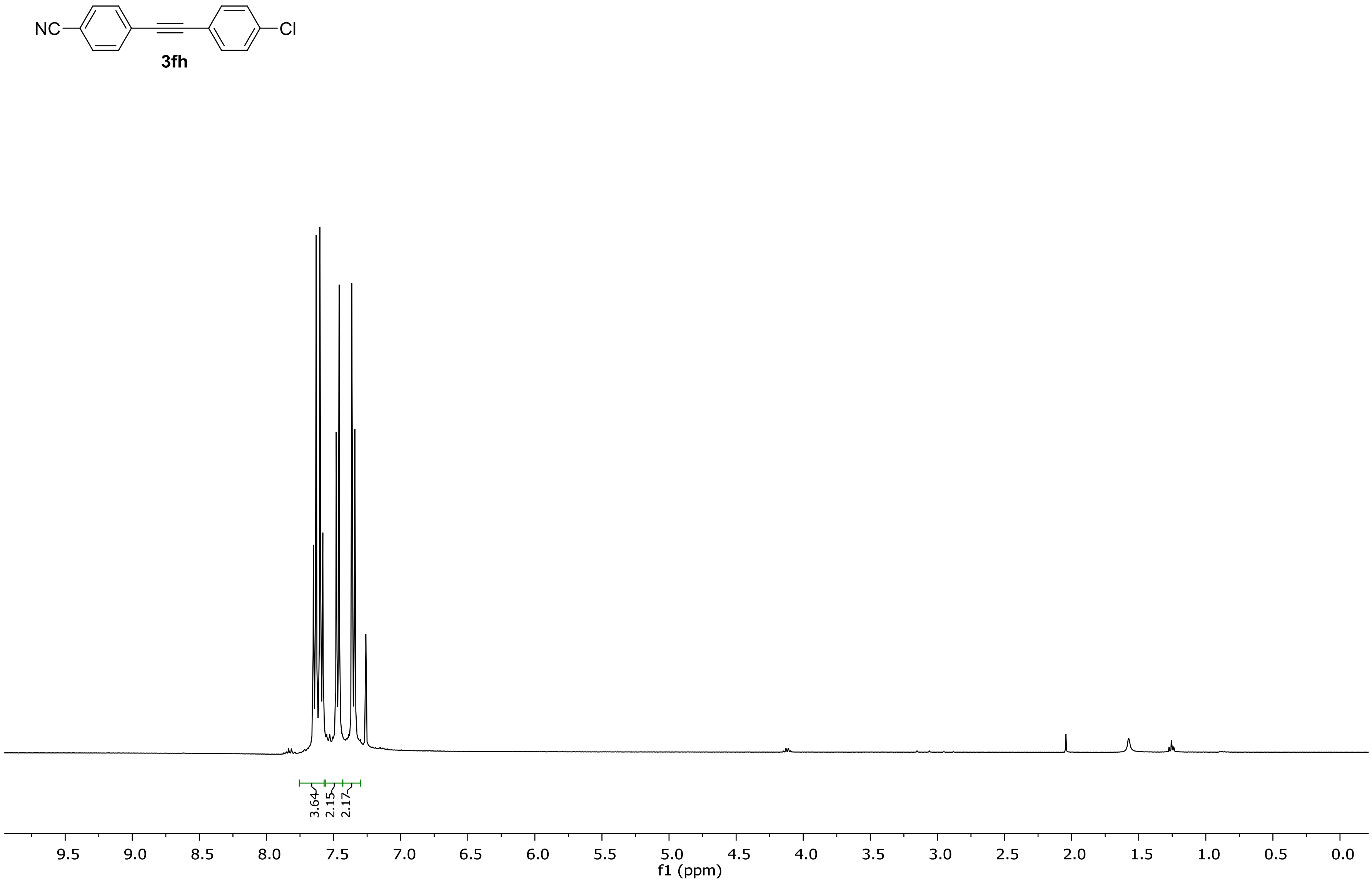
NC
Cl
3fh
3.64
2.15
2.17
9.5
9.0
8.5
8.0
7.5
7.0
6.5
6.0
5.5
5.0
4.5
4.0
3.5
3.0
2.5
2.0
1.5
1.0
0.5
0.0
f1 (ppm)

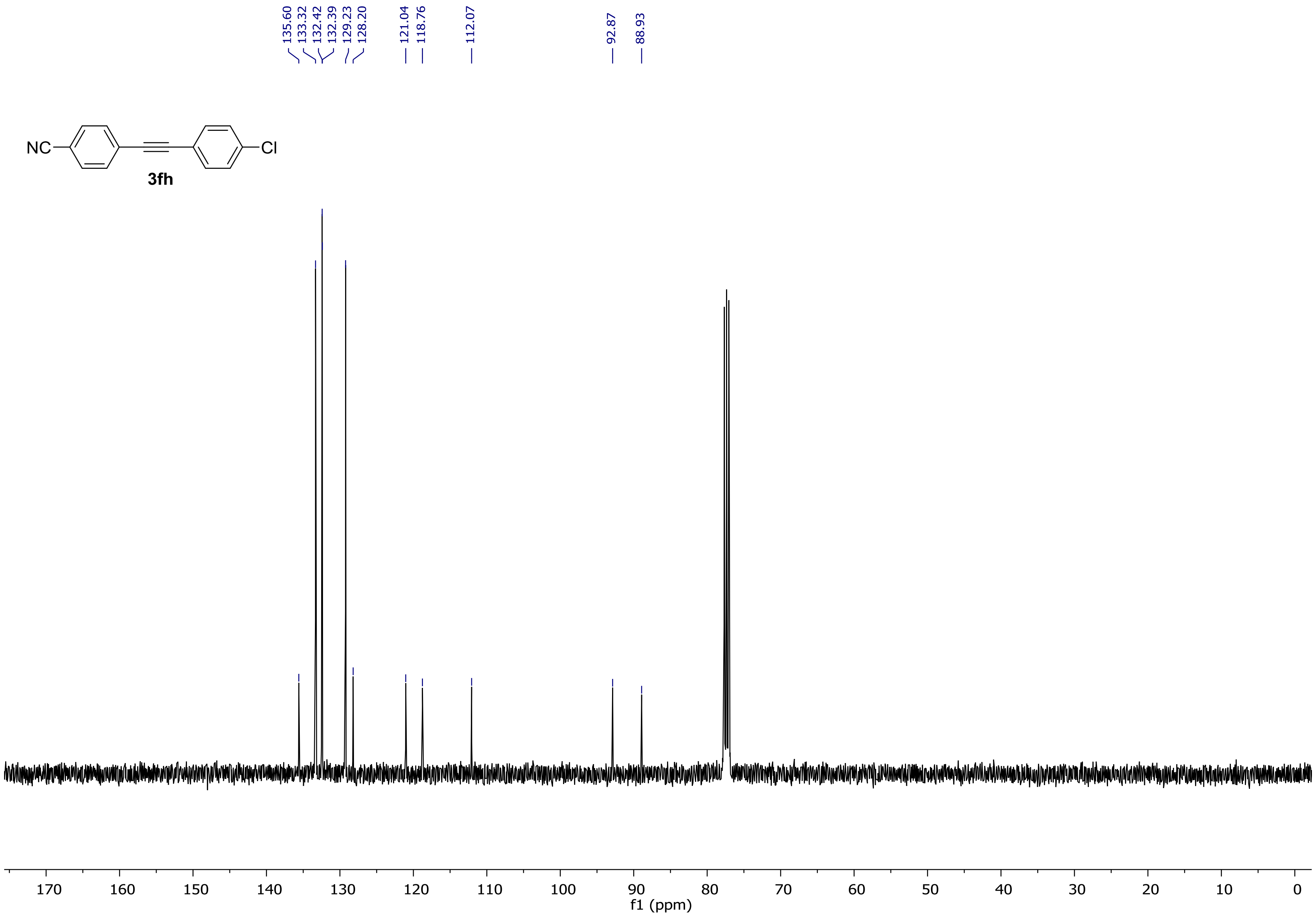
135.60
133.32
132.42
132.39
129.23
128.20
121.04
118.76
112.07
92.87
88.93
NC
Cl
3fh
170
160
150
140
130
120
110
100
90
80
70
60
50
40
30
20
10
0
f1 (ppm)

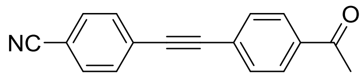

3fi

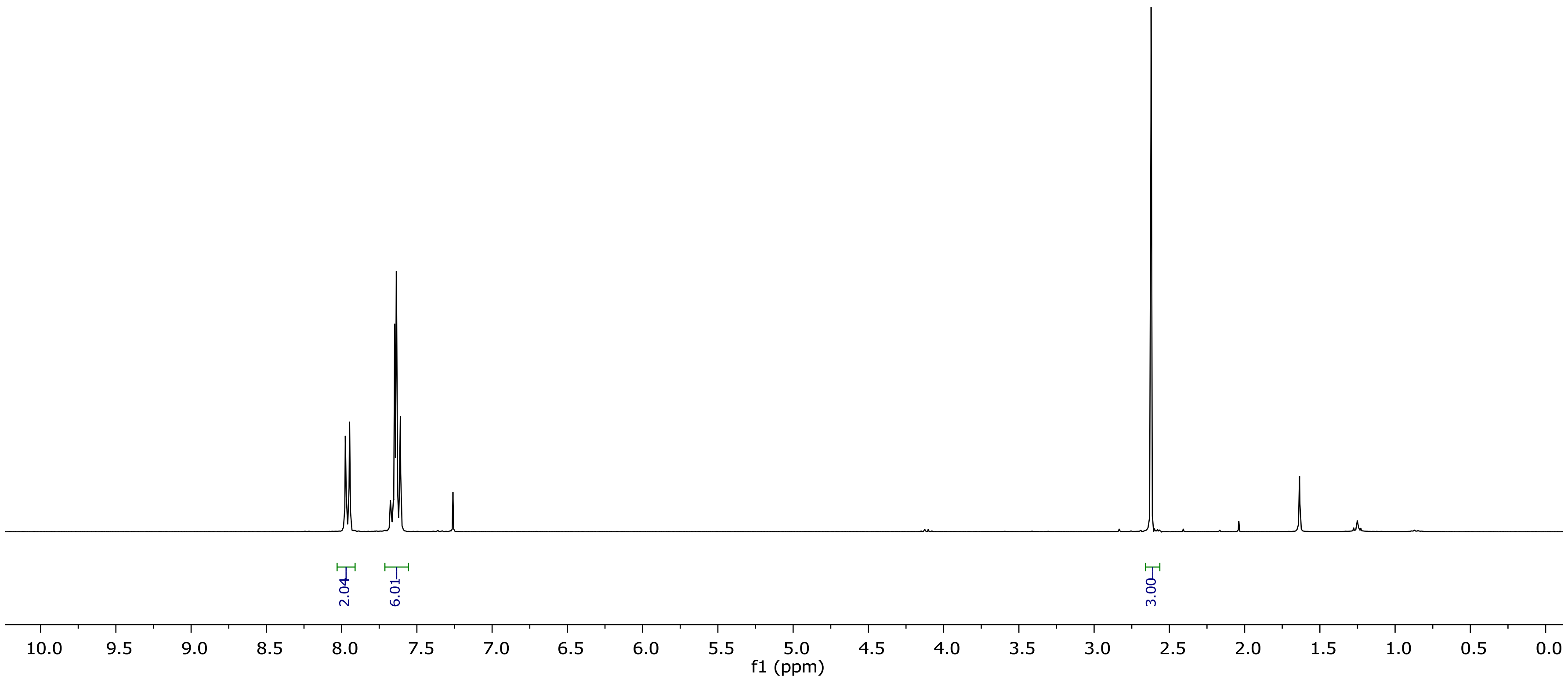
2.04
6.01
3.00
10.0 9.5 9.0 8.5 8.0 7.5 7.0 6.5 6.0 5.5 5.0 4.5 4.0 3.5 3.0 2.5 2.0 1.5 1.0 0.5 0.0
f1 (ppm)

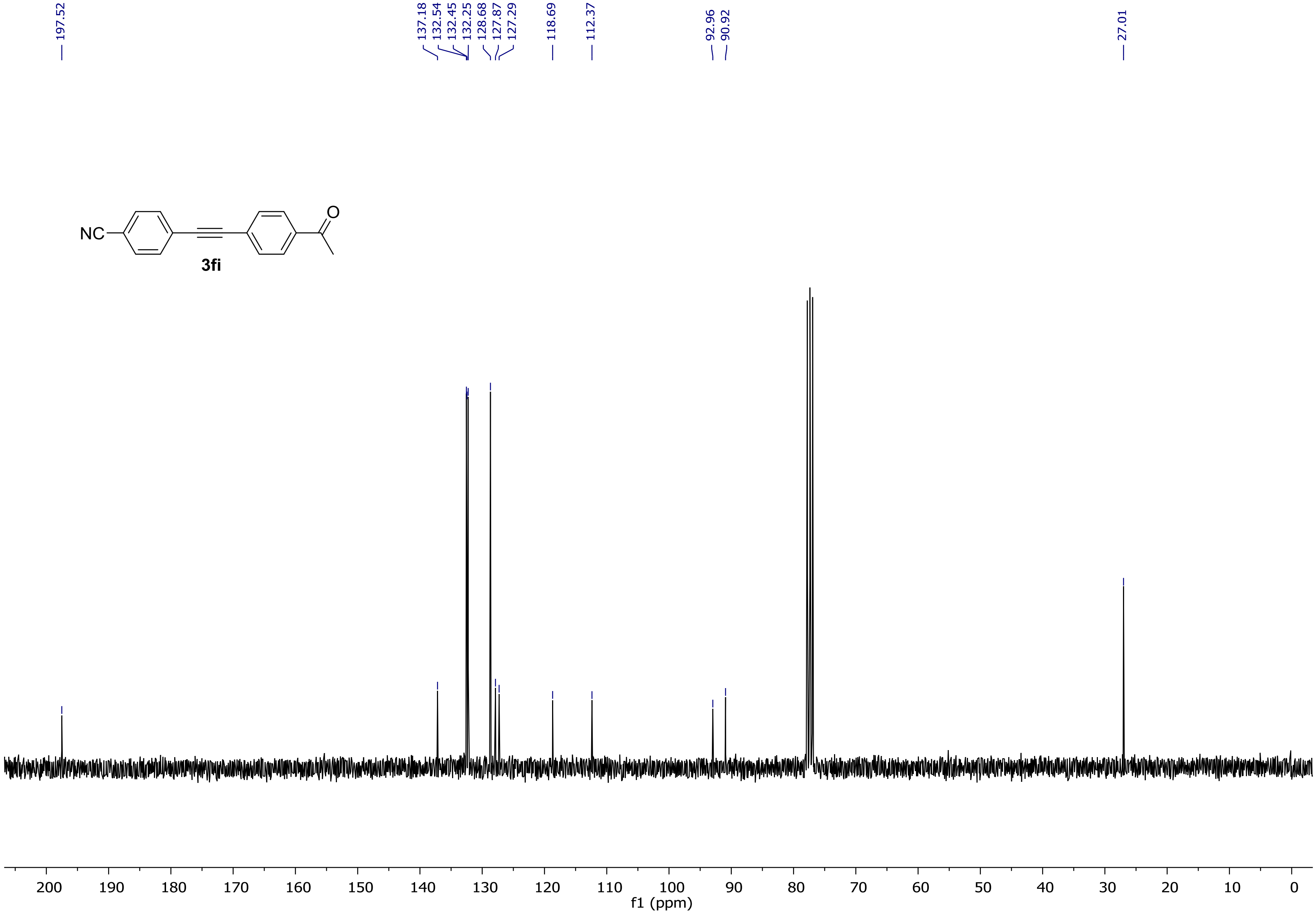
197.52
137.18
132.54
132.45
132.25
128.68
127.87
127.29
118.69
112.37
92.96
90.92
27.01
NC
O
200
190
180
170
160
150
140
130
120
110
100
90
80
70
60
50
40
30
20
10
0
f1 (ppm)

3fi

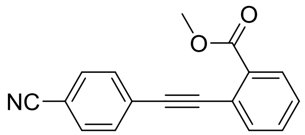

NC
O
O

3fj

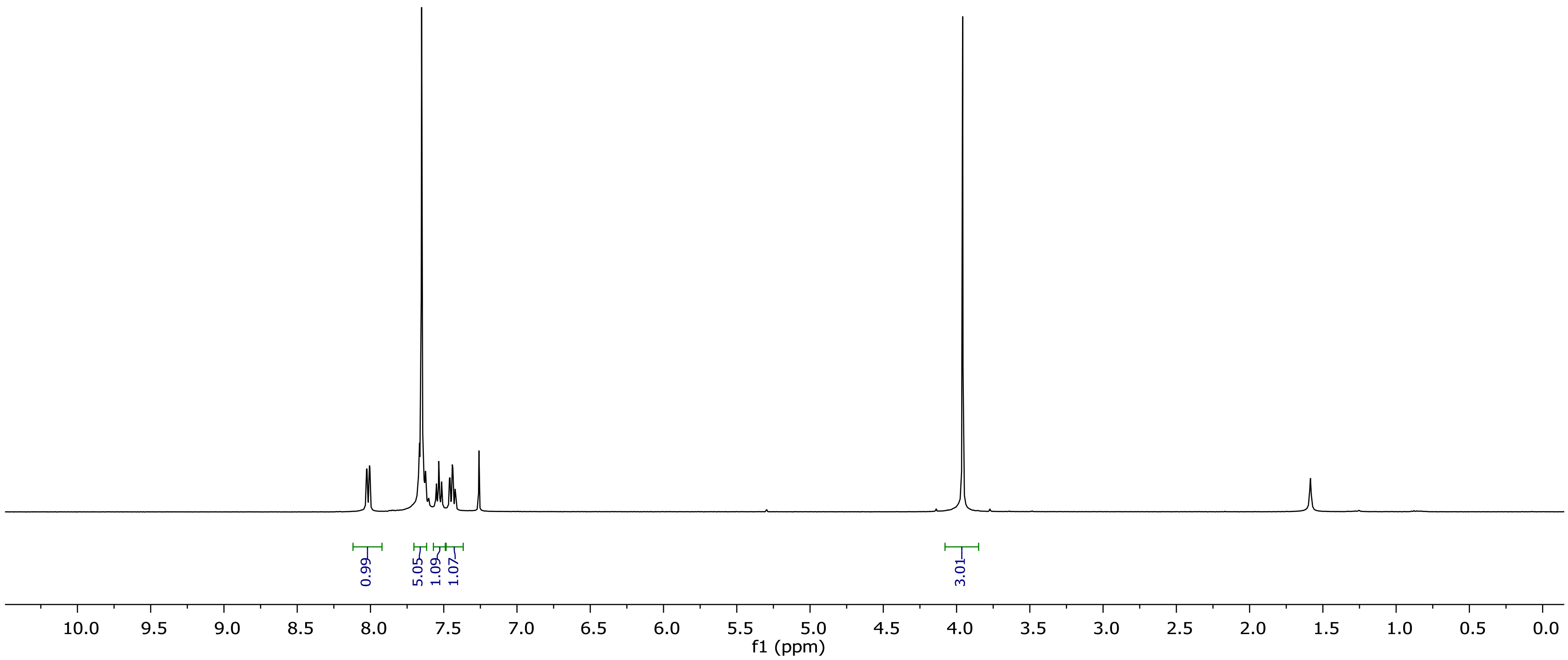

0.99
5.05
1.09
1.07
3.01
10.0
9.5
9.0
8.5
8.0
7.5
7.0
6.5
6.0
5.5
5.0
4.5
4.0
3.5
3.0
2.5
2.0
1.5
1.0
0.5
0.0
f1 (ppm)

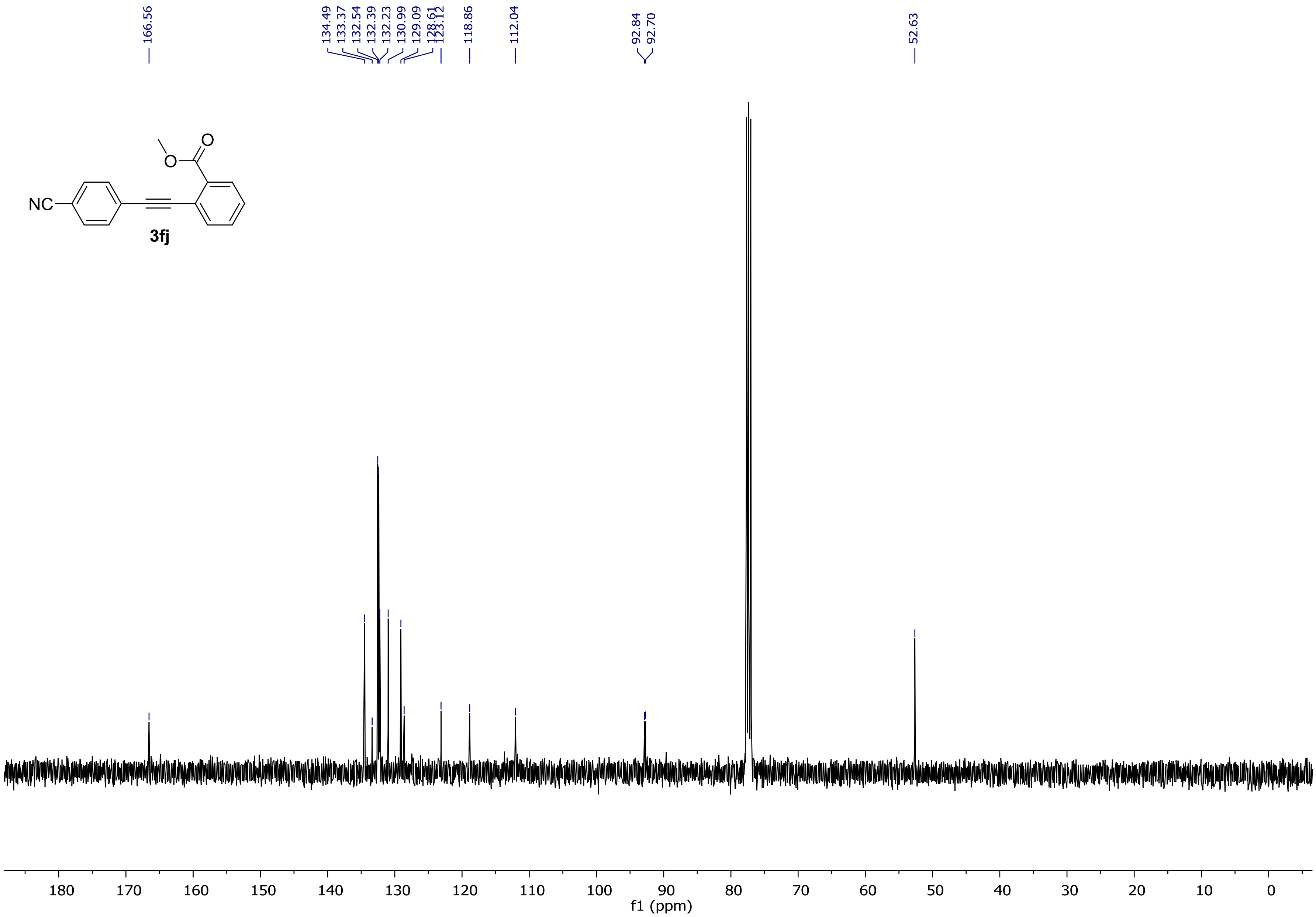
166.56
134.49
133.37
132.54
132.39
132.23
130.99
129.09
128.61
123.12
118.86
112.04
92.84
92.70
52.63
NC
O
O
3fj
f1 (ppm)

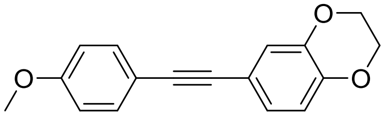

3ck

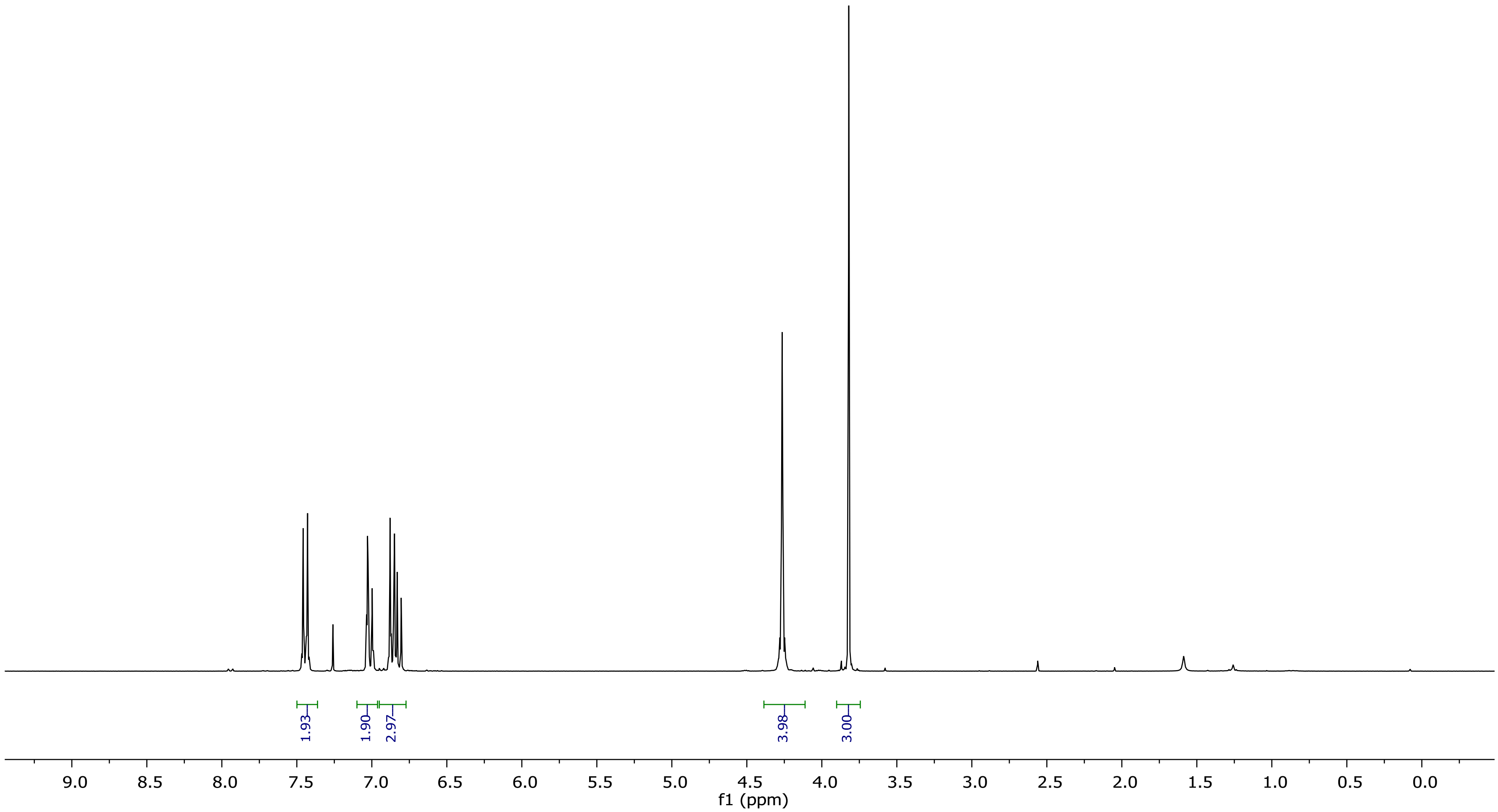

1.93
1.90
2.97
3.98
3.00
9.0
8.5
8.0
7.5
7.0
6.5
6.0
5.5
5.0
4.5
4.0
3.5
3.0
2.5
2.0
1.5
1.0
0.5
0.0
f1 (ppm)

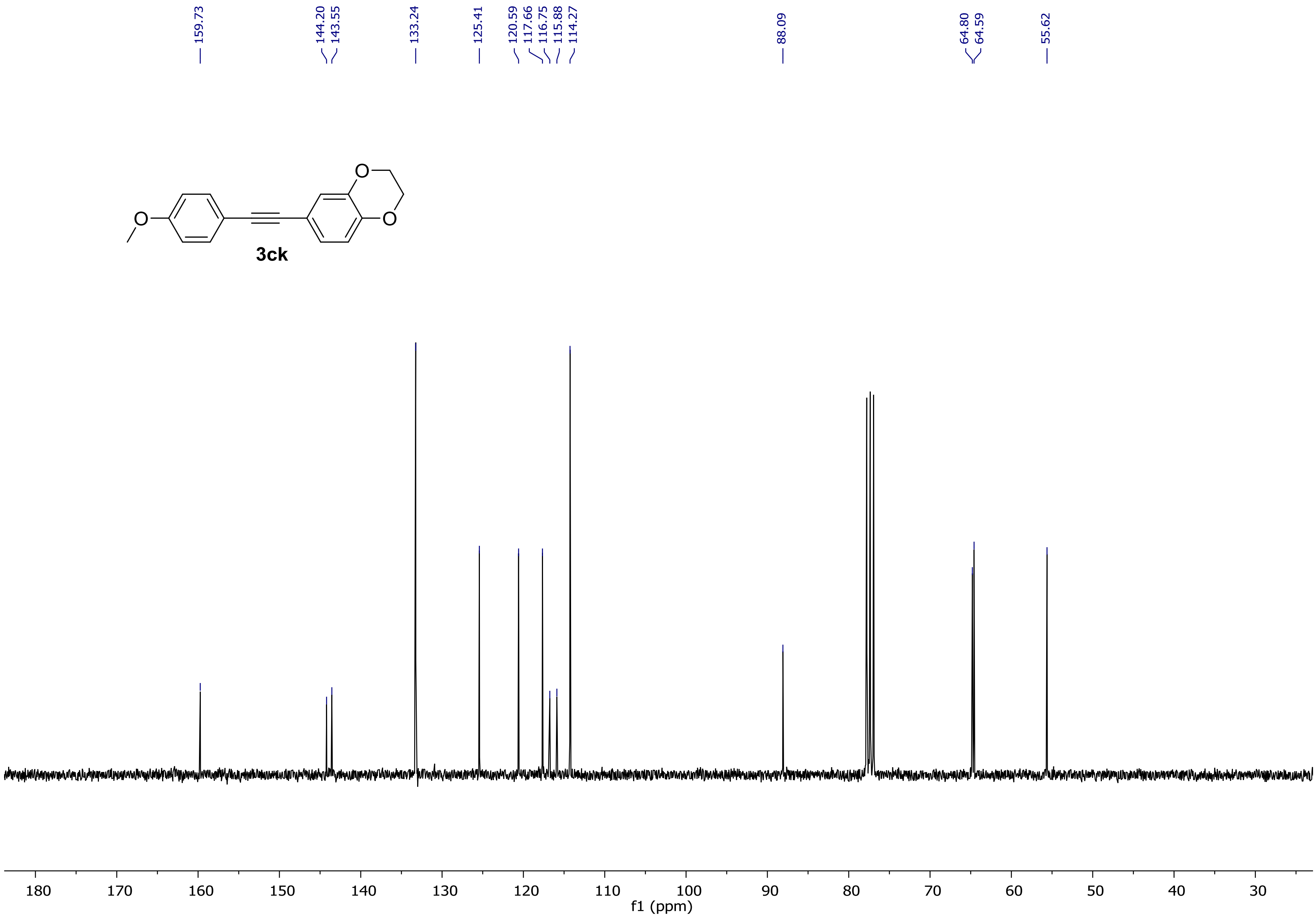

159.73
144.20
143.55
133.24
125.41
120.59
117.66
116.75
115.88
114.27
88.09
64.80
64.59
55.62
3ck
180 170 160 150 140 130 120 110 100 90 80 70 60 50 40 30
f1 (ppm)

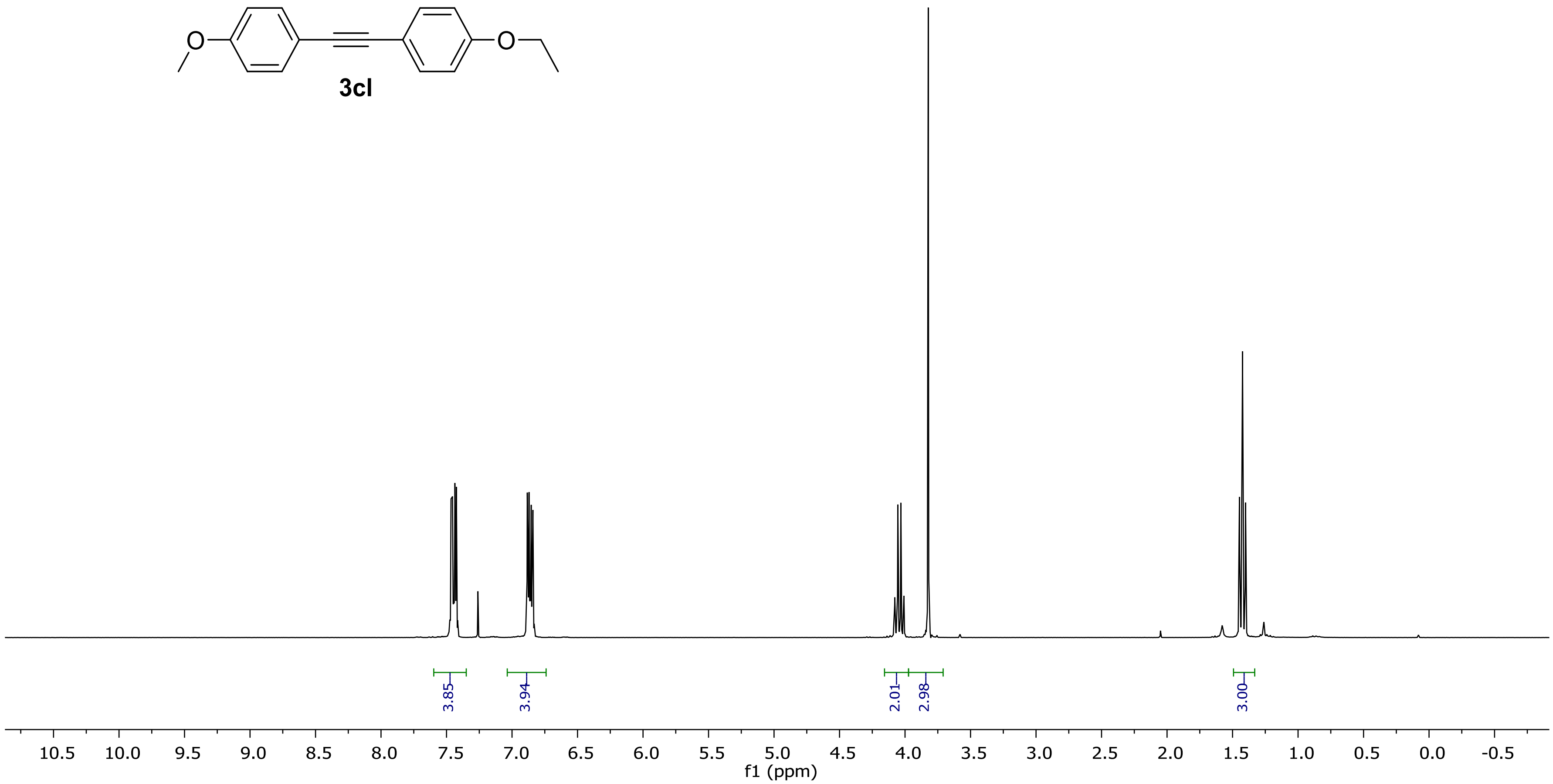

3cl
3.85
3.94
2.01
2.98
3.00
10.5 10.0 9.5 9.0 8.5 8.0 7.5 7.0 6.5 6.0 5.5 5.0 4.5 4.0 3.5 3.0 2.5 2.0 1.5 1.0 0.5 0.0 -0.5
f1 (ppm)

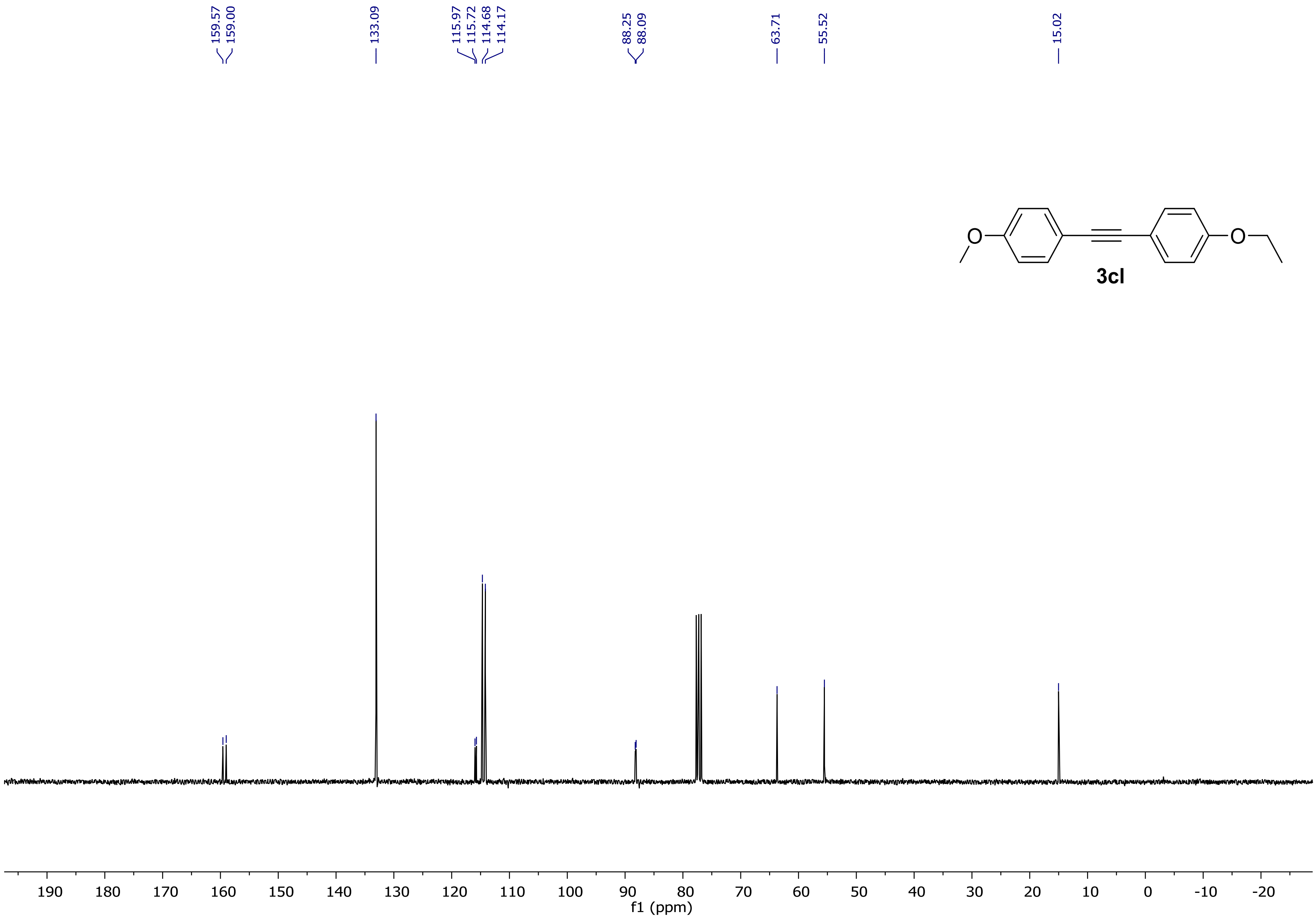
159.57
159.00
133.09
115.97
115.72
114.68
114.17
88.25
88.09
63.71
55.52
15.02
3cl
190 180 170 160 150 140 130 120 110 100 90 80 70 60 50 40 30 20 10 0 -10 -20
f1 (ppm)

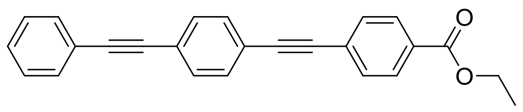

4

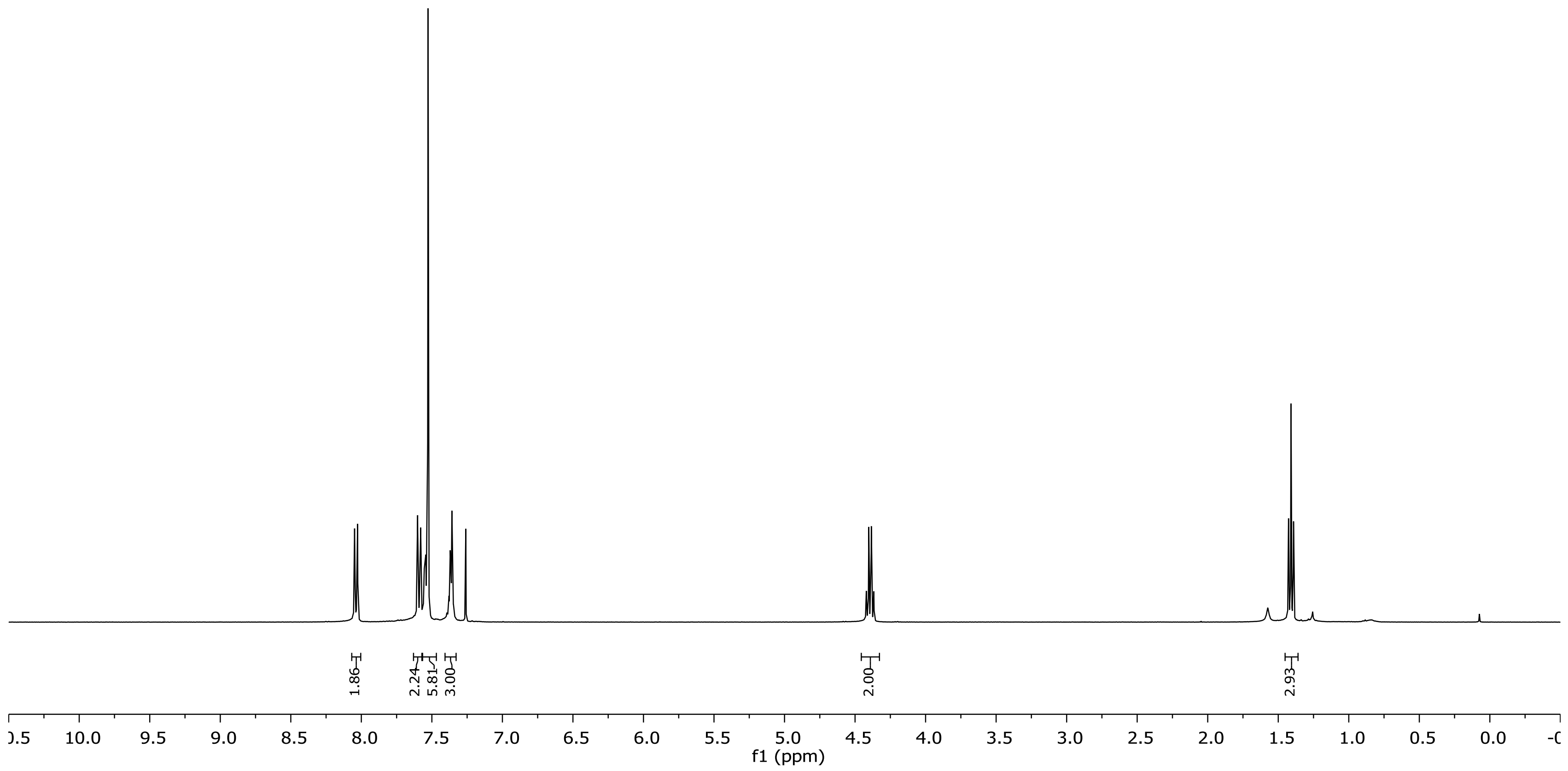

1.86
2.24
5.81
3.00
2.00
2.93
0.5
10.0
9.5
9.0
8.5
8.0
7.5
7.0
6.5
6.0
5.5
5.0
4.5
4.0
3.5
3.0
2.5
2.0
1.5
1.0
0.5
0.0
f1 (ppm)

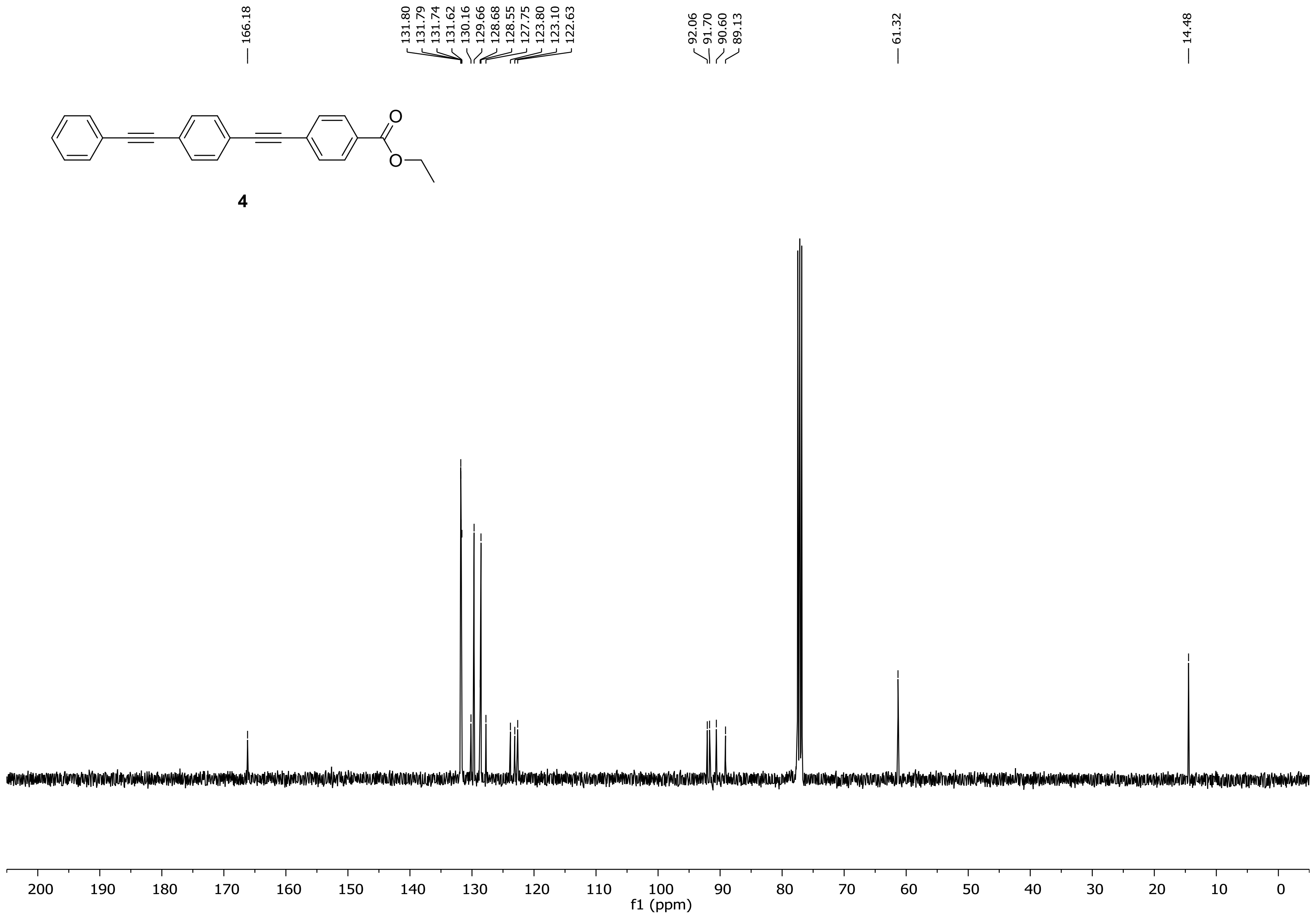
166.18
131.80
131.79
131.74
131.62
130.16
129.66
128.68
128.55
127.75
123.80
123.10
122.63
92.06
91.70
90.60
89.13
61.32
14.48
O
O
4
200 190 180 170 160 150 140 130 120 110 100 90 80 70 60 50 40 30 20 10 0
f1 (ppm)

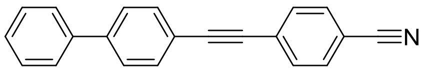

5

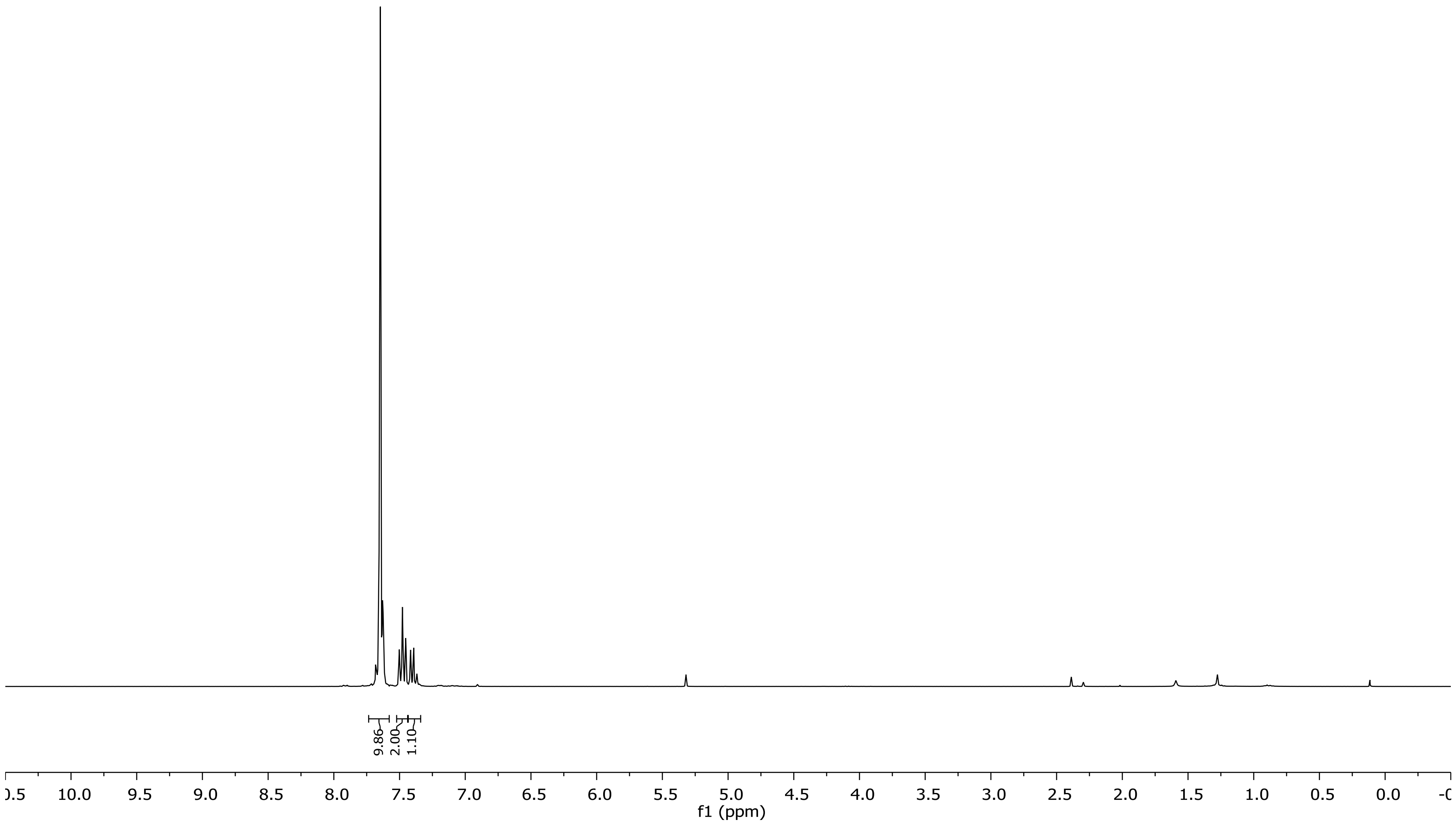

9.86
2.00
1.10
0.5
10.0
9.5
9.0
8.5
8.0
7.5
7.0
6.5
6.0
5.5
5.0
4.5
4.0
3.5
3.0
2.5
2.0
1.5
1.0
0.5
0.0
f1 (ppm)

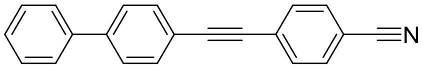

5

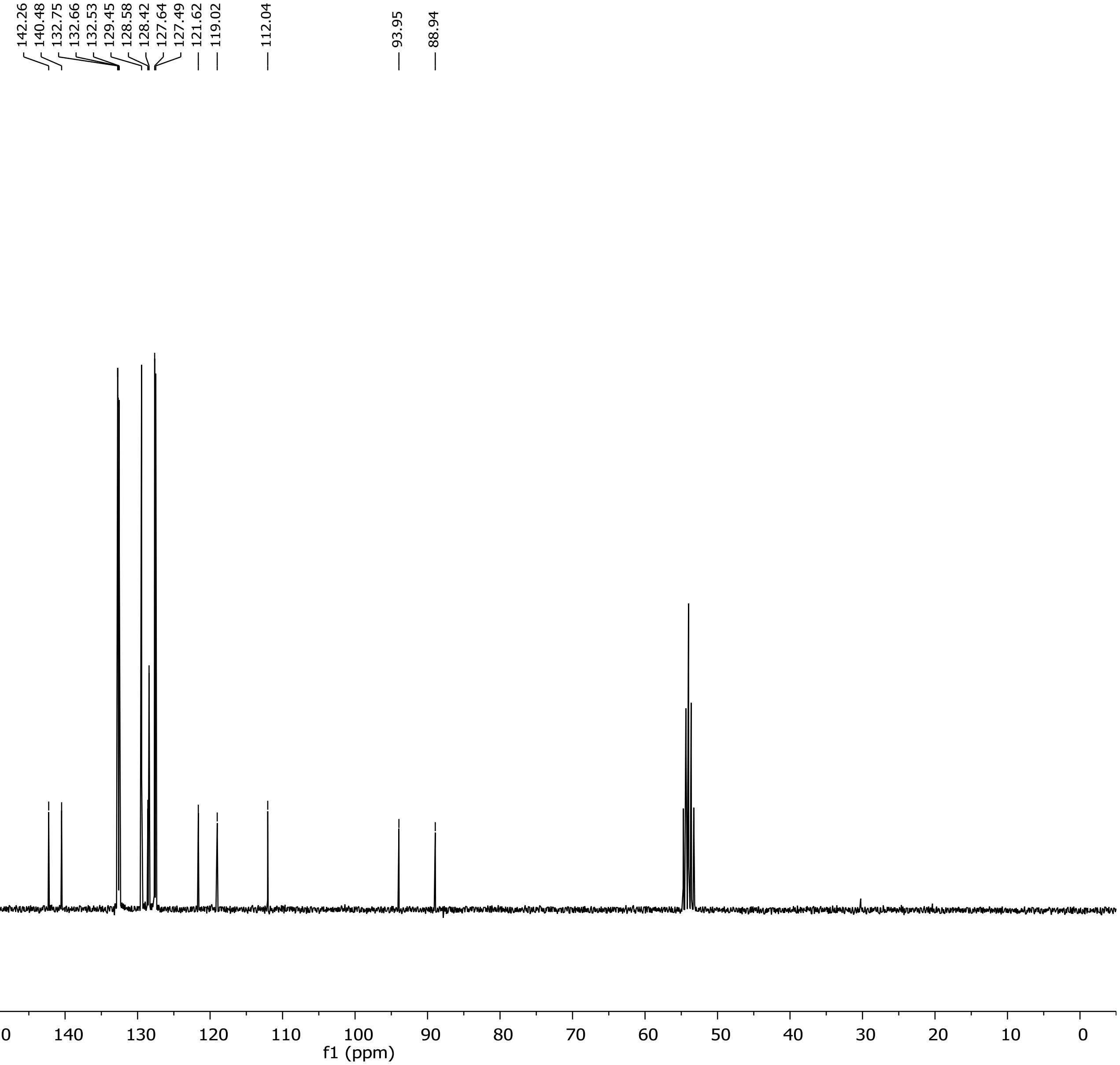

142.26
140.48
132.75
132.66
132.53
129.45
128.58
128.42
127.64
127.49
121.62
119.02
112.04
93.95
88.94
200
190
180
170
160
150
140
130
120
110
100
90
80
70
60
50
40
30
20
10
0
f1 (ppm)
